# Feasibility of the “LvL UP” trial: a pilot sequential multiple assignment randomised trial of an adaptive, holistic mHealth lifestyle coaching intervention

**DOI:** 10.1186/s12966-025-01869-7

**Published:** 2026-01-03

**Authors:** Shenglin Zheng, Oscar Castro, Jacqueline Louise Mair, Ahmad I. Jabir, Sarah Yi Xuan Tan, Akshaye Shenoi, Samarth Negi, Ruth Rachael Mathews, Rachel Woon Sim Koh, Xiaoxi Yan, Bibhas Chakraborty, E. Shyong Tai, Rob M. van Dam, Florian von Wangenheim, Elgar Fleisch, Konstadina Griva, Tobias Kowatsch, Falk Müller-Riemenschneider

**Affiliations:** 1 Saw Swee Hock School of Public Health, National University of Singapore, National University Health System, Tahir Foundation Building, 12 Science Drive 2, , Singapore, #09-01V 117549 Singapore; 2 Future Health Technologies, Singapore-ETH Centre, Campus for Research Excellence and Technological Enterprise (CREATE), Singapore, Singapore; 3 Office of Research, Lee Kong Chian School of Medicine, Nanyang Technological University, Singapore, Singapore; 4 Centre for Quantitative Medicine, Duke-NUS Medical School, Singapore, Singapore; 5 Department of Statistics and Data Science, National University of Singapore, Singapore Singapore,; 6 Department of Biostatistics and Bioinformatics, Duke University, Durham, NC USA; 7 Yong Loo Lin School of Medicine, National University of Singapore, National University Health System, Singapore, Singapore; 8 Department of Exercise and Nutrition Sciences, Milken Institute School of Public Health, George Washington University, Washington, DC USA; 9 Centre for Digital Health Interventions, Department of Management, Technology, and Economics, ETH Zürich, Zürich, Switzerland; 10 Centre for Digital Health Interventions, Institute of Technology Management, University of St. Gallen, St. Gallen, Switzerland; 11 Institute for Implementation Science in Health Care, University of Zürich, Zürich, Switzerland; 12 School of Medicine, University of St. Gallen, St. Gallen, Switzerland; 13 Digital Health Centre, Berlin Institute of Health, Charité – Universitätsmedizin Berlin, Berlin, Germany

**Keywords:** SMART, Adaptive interventions, Digital health, MHealth, Prevention

## Abstract

**Background:**

Mobile Health (mHealth) interventions are promising for addressing the growing burden of noncommunicable diseases and common mental disorders but often focus on single domains and lack adaptability. LvL UP (“Level Up”) is a holistic mHealth lifestyle coaching intervention that integrates physical activity, diet, and emotional regulation. It provides blended coaching support through an app-based conversational agent with adaptive human support. This pilot trial assessed the feasibility of delivering the LvL UP intervention and implementing its adaptive procedures using a sequential multiple assignment randomised trial (SMART) design.

**Methods:**

This eight-week pilot trial was conducted from 29 March to 1 August 2024. We recruited adults in Singapore aged 21–59 at risk of chronic conditions. Participants were randomised 2:1 to the intervention (LvL UP app with a peer supporter–LvL UP Buddy) or comparison (control app with educational resources). After four weeks, non-responders (defined as completing < 6 digital coaching sessions or rated session usefulness < 4/5) were re-randomised 1:1 to continue or receive three additional motivational interviewing (MI)-informed sessions with a human coach; responders remained on their original allocation. Primary outcomes included feasibility indicators: recruitment, LvL UP Buddy enrolment, non-responder rate, trial retention, data completion rate, and intervention engagement. Secondary outcomes measured changes from baseline to eight weeks in mental well-being, psychological distress, physical activity, sleep duration, and fruit and vegetable intake. Six progression criteria were prespecified to guide advancement to a definitive trial.

**Results:**

Of the 458 individuals screened, 394 were eligible, and 123 were enrolled (82 interventions; 41 controls). Most intervention participants (95.1%) were paired with a LvL UP Buddy. Thirty-eight participants (46.3%) were non-responders; of those assigned to MI-informed sessions, 52.6% (10/19) completed all three. Eight-week retention was high (91.5% intervention; 92.7% control), with 12.2% missing data. Positive trends were observed in mental well-being (2.12, 95% CI [-0.58, 4.82]), psychological distress (-0.94 [-2.08, 0.20]), and sleep duration (0.49 h/week [0.17, 0.82]). The study met five of six prespecified progression criteria: recruiting ≥ 60 participants within six weeks, achieving ≥ 75% retention, maintaining ≤ 20% missing data, obtaining a 40–60% non-responder rate, and showing a positive change in ≥ 1 health-related outcome. The digital coaching session adherence fell below the target (39.5% vs. 70%).

**Conclusions:**

LvL UP was feasible to deliver and evaluate using a SMART design. The results provide strong operational guidance and a solid foundation for the refinement and implementation of a fully powered trial.

**Trial registration:**

ClinicalTrials.gov, TRN: NCT06360029, Registration date: 7 April 2024.

**Supplementary Information:**

The online version contains supplementary material available at 10.1186/s12966-025-01869-7.

## Background

Healthy ageing, defined by the World Health Organisation (WHO) as maintaining the functional ability for physical and mental health in older age, is an urgent global priority as populations age rapidly [1, 2]. Although often associated with older adults, healthy ageing begins much earlier in life [3]. Targeting modifiable lifestyle risk factors, such as physical inactivity and unhealthy diets, in younger and middle-aged populations, can help preserve high functional capacity and delay age-related decline [3]. However, sustaining these behaviours is challenging without concurrent mental health support. Poor mental health is associated with physical inactivity or unhealthy dietary habits, creating a reinforcing cycle that increases non-communicable disease (NCD) risk and worsens common health disorders (CMDs) [4–6]. Addressing this population-wide challenge requires scalable and accessible solutions that can promote behaviour change across diverse populations [7–9]. 

Mobile health (mHealth) interventions offer a promising approach to delivering such solutions at scale [10]. Evidence suggests that mHealth interventions can promote healthy behaviours and improve health outcomes, especially in settings with limited access to conventional healthcare [7–9, 11]. However, many mHealth interventions target a single behaviour, which limits their ability to address the complex interconnections between lifestyle behaviours and mental health. Recognising these interdependencies, holistic mHealth interventions that simultaneously target physical activity, diet, and mental health have shown benefits for weight management, lifestyle improvement, and mental well-being [12–15]. 

While holistic mHealth interventions show promise, augmenting them with human support may further improve adherence and outcomes [11, 16, 17]. However, widespread human support is costly and difficult to scale [11, 16]. Conversational agents offer a lower-cost and scalable alternative, although their capacity to fully replicate the nuances of human support remains unclear [18–20]. Adaptive intervention strategies, tailoring support intensity based on individual needs, can optimise both effectiveness and resource allocation [21, 22]. For instance, participants can begin with digital support and escalate to human-delivered coaching only when needed.

Building on these principles, we developed LvL UP (“Level Up”) 3.0, an adaptive, holistic mHealth lifestyle coaching intervention for the prevention of NCDs and CMDs. It includes a smartphone application (LvL UP app) featuring an automated conversational agent and self-management tools, plus peer support from a LvL UP Buddy. Participants who do not meet pre-specified response criteria are escalated to receive additional human coaching. The intervention was developed and refined between 2021 and 2023, informed by systematic reviews, [11, 15, 23] market analyses, [24, 25] user-centred studies, [26, 27] and feasibility studies [28–30]. 

To evaluate LvL UP, we use a sequential multiple assignment randomised trial (SMART) design, which allows participants to progress through multiple stages with more than one randomisation point [22, 31]. While SMART designs have been used in treatment-focused trials, their application in preventive mHealth interventions is limited [32, 33]. Existing SMARTs have primarily focused on stage-specific comparisons instead of viewing the intervention as a continuous process, and often lack control arms [32]. Given the novelty and methodological complexity of applying a SMART design to a holistic mHealth intervention, a pilot trial was necessary to assess the viability of the trial procedures, refine its implementation, and ensure both scalability and methodological robustness before conducting a definitive trial. This is also in line with the Medical Research Council’s guidance on developing and evaluating complex interventions [34]. Therefore, this study aimed to assess the feasibility of delivering the LvL UP intervention and of implementing its adaptive procedures using a SMART design, focusing on recruitment, the non-responder rate, trial retention, data completion rate, and intervention engagement. We also explored preliminary changes in mental health and behavioural outcomes, comparing adaptive strategies with the control arm. Trial findings were evaluated against pre-specified progression criteria to guide decisions on advancing to a definitive trial.

## Methods

### Trial design and procedure

This eight-week SMART pilot trial was conducted over an approximate four-month period in Singapore, with recruitment beginning on 29 March and all data collection, including qualitative interviews, concluding on 1 August 2024. Baseline assessments were conducted in person at the Saw Swee Hock School of Public Health, National University of Singapore (NUS). The four-week mid-intervention survey was completed online. Eight-week follow-up assessments were conducted primarily in person at NUS; participants unable to attend in person completed the same survey online but did not complete physical assessments.

Ethical approval was obtained from the Institutional Review Boards of NUS (NUS-IRB-2023-421), ETH Zürich (EK-2024-N-13-A), and Nanyang Technological University (NTU-IRB-2024-305). All participants provided written informed consent prior to participation. The published protocol is available in Supplementary material 1.

The trial was prospectively registered on ClinicalTrials.gov (NCT06360029) on 7 April 2024. Reporting follows the CONSORT extension for randomised pilot and feasibility trials (Supplementary material 2) [35].

Participants were randomised in a 2:1 ratio to either the intervention or the control arm. After four weeks, non-responders (defined as completing fewer than six digital coaching sessions or rating session usefulness below four out of five) in the intervention arm were re-randomised (1:1) to either continue the intervention (Group B) or receive additional human support (Group C). “Responders” continued without change (Group A), while control participants remained on the control app (Group D).

**Fig. 1:**
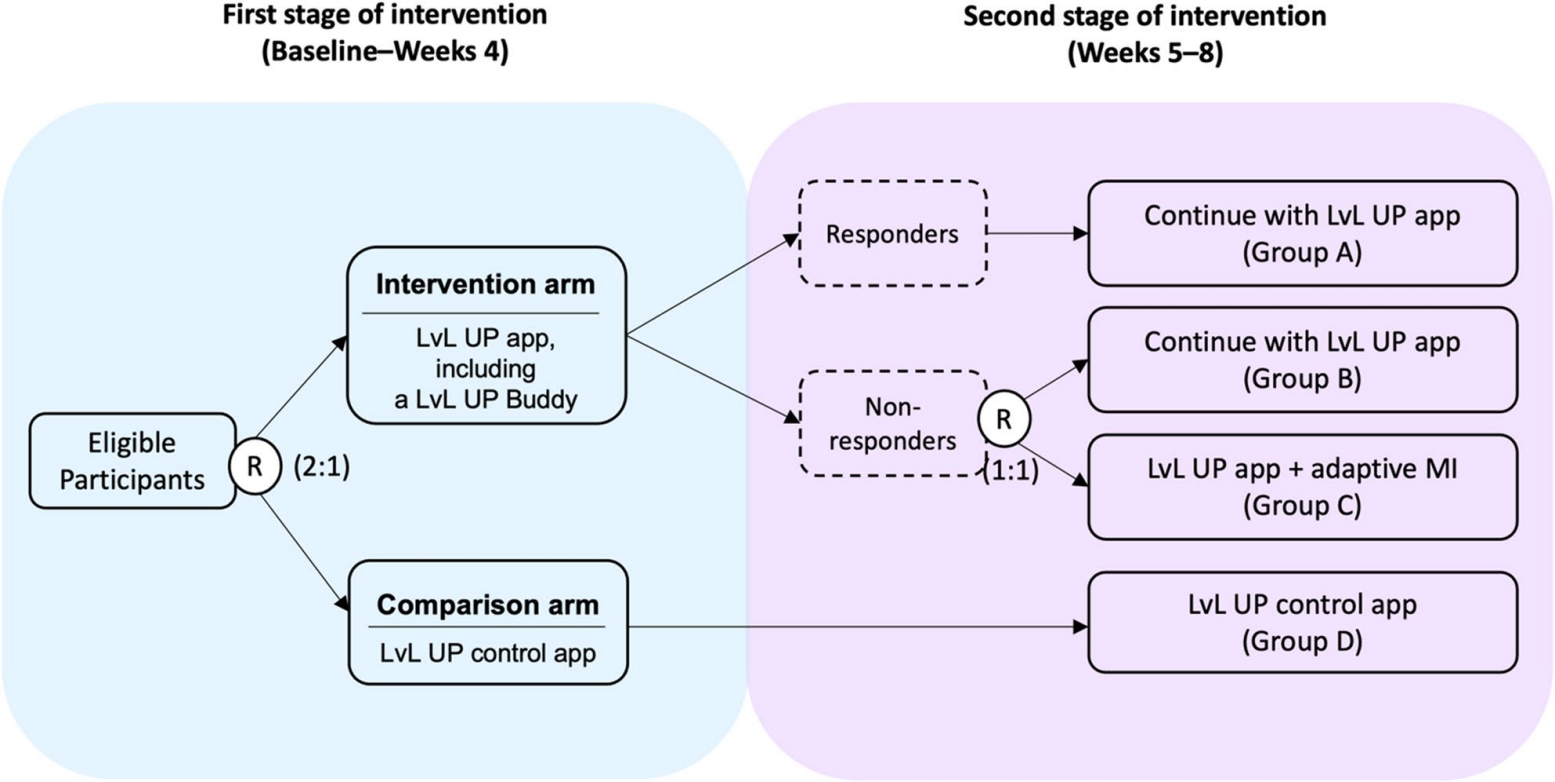
Sequential Multiple Assignment Randomised Trial (SMART) design with a control arm for LvL UP. R: randomisation; Adaptive MI: three weekly motivational interviewing-informed sessions with human coaches; Non-responders were defined as those completing < 6 digital coaching sessions or rating session usefulness <4/5

Two adaptive intervention strategies were embedded within this trial design (Fig. 1): Strategy #1 (Group A and Group B) used only the LvL UP app with a LvL UP Buddy (a nominated peer who provided support) for eight weeks, regardless of response status; Strategy #2 (Group A and Group C) involved escalating to additional human support for non-responders in the second stage.

Participants received up to SGD 250 in grocery vouchers: SGD 90 (baseline assessment), SGD 20 (four-week survey), SGD 110 (follow-up assessment), and SGD 30 (qualitative interview).

### Participants

Participants were recruited via social media (Telegram, Facebook, Instagram), university email lists, community outreach through a recruitment agency, and referrals. Interested individuals completed an online screening survey and were invited to attend baseline assessments if eligible.

Eligibility criteria were as follows: aged 21 to 59 years; Singapore citizens or permanent residents; residing in Singapore during the study; English proficiency; smartphone ownership (Apple iOS ≥ 12.4 or Android ≥ 8.0) with internet access; and at risk of NCDs or CMDs. Risk was assessed using a composite score: Patient Health Questionnaire-4 scores ≥ 3 [36] or at least two of the following: (1) insufficient physical activity (< 150 minutes of moderate-to-vigorous physical activity (MVPA)/week); (2) unhealthy diet (< 2 servings of fruit/vegetables or > 1 serving of sugary beverages/fast food daily); (3) family history of diagnosed physical or mental health conditions; or (4) Body Mass Index ≥ 23 kg/m² [37]. Exclusion criteria included a current diagnosis of chronic disease, pregnancy, use of medications affecting blood pressure or glucose metabolism, or participation in the study as a LvL UP Buddy.

### Intervention arm

#### First stage of intervention (weeks 1–4): LvL UP app with LvL UP Buddy

**Fig. 2:**
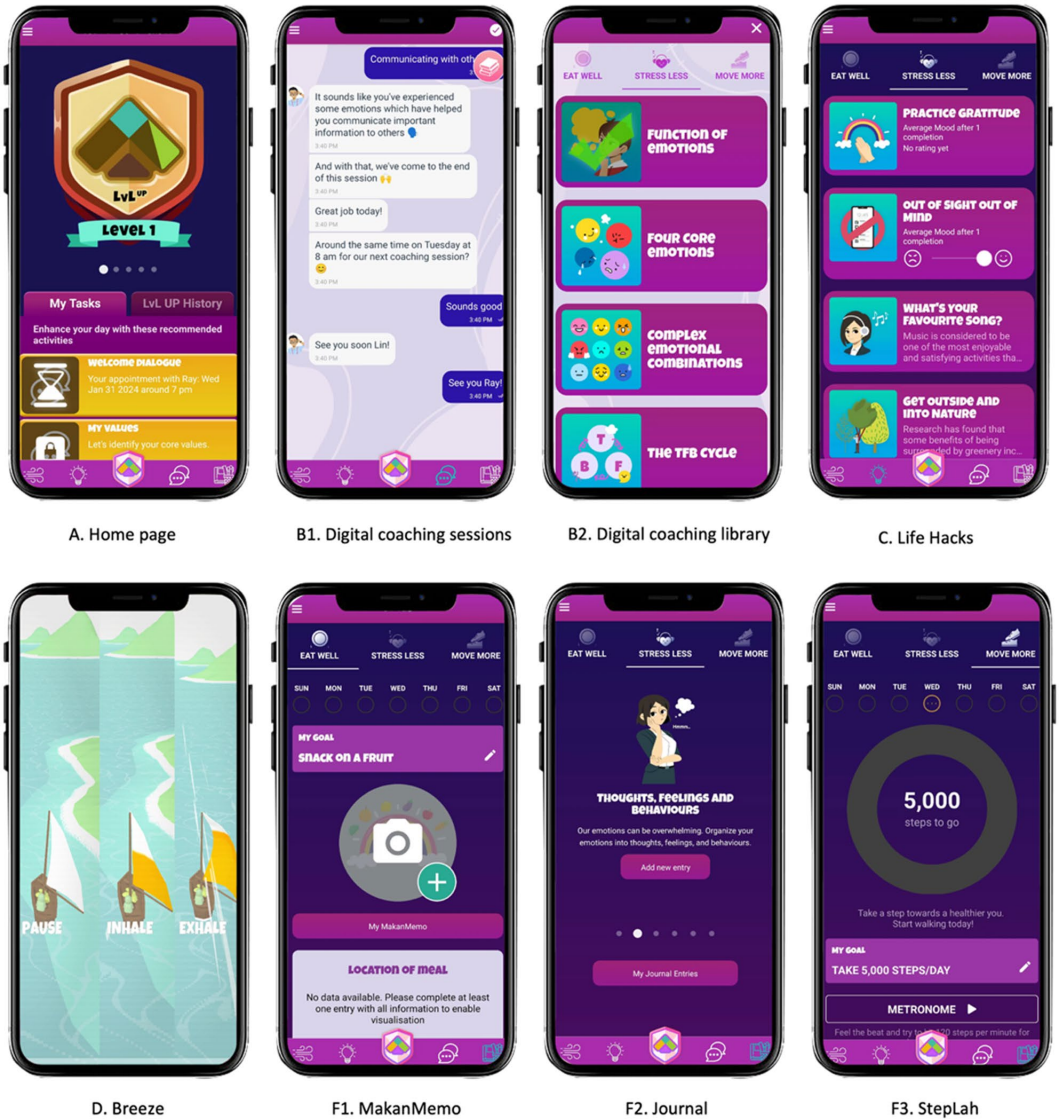
Screenshots captured from the LvL UP app (version 3.0). B1–B2: Digital coaching sessions delivered by the rule-based conversational agent. F1–F3: Self-regulation tools

Participants in the intervention arm downloaded the LvL UP app (version 3.0), which promotes physical activity, healthy diet, and emotional regulation through four core components (Fig. 2):

1. Digital coaching sessions: 30 brief coaching sessions (5–8 min each) covering health literacy and psychoeducation via a rule-based conversational agent.
2. Life Hacks: 48 actionable, trackable health tips for habit formation.
3. Breeze: A breathing training game with biofeedback for stress reduction.
4. Self-regulation tools: “MakanMemo” food diary, Journaling, and “StepLah” step tracker for behaviour monitoring and self-reflection.

Participants were encouraged to complete 12 digital coaching sessions and use at least one additional component daily. Engagement was supported through “My Tasks”, in-app virtual rewards, push notifications, WhatsApp reminders, and weekly emails.

Intervention participants were asked to nominate a LvL UP Buddy for peer support [38]. Buddies received weekly WhatsApp prompts to encourage participants, share healthy tips, and co-engage in healthy activities. Buddies were reimbursed up to SGD 70 for completing the four- and eight-week surveys, as well as for participating in qualitativeinterviews.

#### Second stage of intervention (weeks 5–8): motivational interviewing (MI)-informed coaching for non-responders

Non-responders re-randomised to Group C received three weekly WhatsApp-based sessions with trained human coaches (RRM and RK), both of whom had psychology backgrounds and MI training. Each session (30–40 minutes) followed core MI strategies (including open-ended questions, reflections, affirmations, and summaries), and was structured around engagement, focusing, evoking, and planning [39]. Coaches documented the strategies used and received weekly feedback from a certified MI practitioner (KG).

### Control arm

Participants in the control arm (Group D) received public health information on physical activity, diet, and mental health via WhatsApp (Supplementary material 3), and used a control app with an identical interface but no active intervention components. The control app only collected mood data using the International Positive and Negative Affect Schedule Short-Form every 2–3 days [40]. 

### Outcomes

#### Feasibility

Feasibility was assessed across six indicators:

5. Recruitment: Channel-specific enrolment-to-eligibility ratios and the proportion of participants recruited within six weeks.
6. LvL UP Buddy uptake: The proportion of intervention participants successfully paired with a LvL UP Buddy.
7. Non-responder rate: The proportion of non-responders vs. responders at week five.
8. Trial retention: The proportion of participants completing the eight-week follow-up.
9. Data completion rate: The percentage of missing data for each outcome at follow-up.
10. Intervention engagement: The number of active app days (days with at least one completed task), app component usage, and MI-informed session attendance.

Additional implementation outcomes included usability and user satisfaction. Usability was assessed using the System Usability Scale (SUS; scores 0–100, where > 70 is acceptable, 50–70 is marginally acceptable, and < 50 is unacceptable) [41]. User satisfaction was assessed using the Net Promoter Score (NPS; scores −100 to + 100), calculated as the percentage of promoters (scores 9–10) minus detractors (scores 0–6) [42]. Both measures were collected via the four-week online survey.

#### Mental health and lifestyle outcomes

Mental well-being was measured using the Warwick-Edinburgh Mental Well-being Scale (WEMWBS; 14 items, scores 14–70), with higher scores indicating better mental well-being [43]. Psychological distress was measured using the Kessler Psychological Distress Scale (K6; 6 items, scores 0–24), where higher scores reflect greater distress [44]. 

MVPA (minutes/week) was measured using the International Physical Activity Questionnaire–Long Form [45]. Diet assessment included daily intake of vegetables and fruits using a modified Food Frequency Questionnaire [46]. Sleep duration (hours/day) was measured using one item from the Pittsburgh Sleep Quality Index [47]. 

### Pre-specified progression criteria

Progression criteria were developed in collaboration with the study’s Scientific Advisory Board. Advancement to a definitive trial was supported if the following targets were met: 1) ≥ 50% of target recruitment within six weeks; 2) a 40–60% non-responder rate; 3) ≥ 75% trial retention; 4) ≤ 20% missing data for the mental well-being outcome; 5) ≥ 70% of intervention participants completing 12 digital coaching sessions; 6) a positive directional change in at least one health-related outcome. If any criterion was not met, the protocol would be revised prior to the definitive trial.

### Assessment of harms

Participants were encouraged to report adverse events via an open-ended question in the follow-up assessment. No serious harm was anticipated.

### Sample size and randomisation

A precision-based approach was used for sample size estimation [48]. Assuming a 50% non-responder rate, 5% Type I error, and 30% margin of error, a minimum of 97 participants was required. The final sample size was set at 120 to account for an expected 18% attrition [49].

Randomisation sequences were generated in Stata by the first author (SZ). Simple randomisation was used at baseline, and block randomisation (block size = 4) was applied for re-randomisation at week five. To blind the assessors, participants received sealed packages with study IDs assigned based on arrival order after all baseline assessments. The staff distributing the packages were also blinded to group allocation and did not participate in data collection.

### Data analysis

Of the 123 enrolled participants, one was excluded for age ineligibility, leaving 122 for analysis. Baseline characteristics are presented as frequencies (percentages) for categorical variables and as means ± standard deviations (SDs) or medians (Interquartile ranges [IQR], Q1–Q3) for continuous variables.

Preliminary effectiveness analyses followed the intention-to-treat principle. Generalised estimating equations (GEE) with robust variance estimation and an exchangeable correlation structure were used to estimate changes from baseline across two adaptive intervention strategies versus the control arm. To account for the complex allocation design, a weight-and-replicate method was applied: observations in Group A were replicated and inverse probability weights were assigned: 1.5 for Group A, 3 for Groups B, C, and D [50]. The interaction terms between condition and time were used to test for differential changes in outcomes.

## Results

### Recruitment and allocation

Recruitment and screening took place between March 29 and May 10, 2024, during which 458 individuals were screened; 394 (86.0%) were eligible, and 123 were enrolled. Participants were primarily recruited from Telegram (75/123, 61.0%), word-of-mouth (17/123, 13.8%), community outreach (18/123, 14.6%), and email (11/123, 8.9%). Although smaller in scale, email had the highest conversion rate (11/17, 64.7%), followed by Telegram (75/177, 42.4%) and word-of-mouth (17/58, 29.3%).

**Fig. 3:**
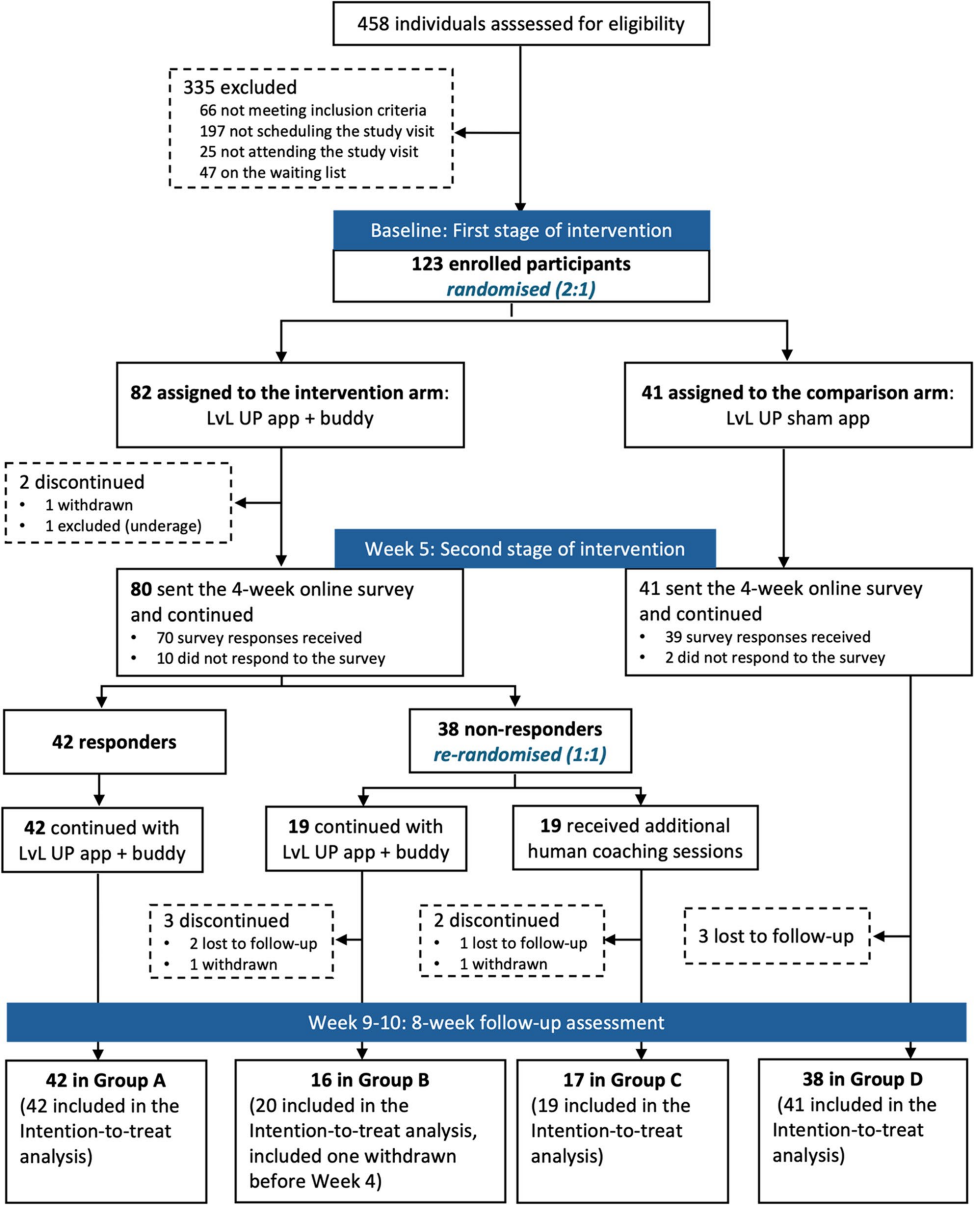
Participant flow chart

At baseline, 82 participants were randomised to the intervention arm and 41 to the control arm (Fig. 3). Most intervention participants (78/82, 95.1%) were successfully paired with a LvL UP Buddy. At week five, 38 non-responders (46.3%) were re-randomised to either continue (Group B; *n* = 19) or receive additional human support (Group C; *n* = 19). Responders continued without change (Group A; *n* = 42). Control participants remined in Group D throughout the study.

### Retention and data completion

At the eight-week follow-up, retention was 91.5% (75/82, intervention) and 92.7% (38/41, control). Of the seven intervention arm dropouts, one was excluded post-randomisation for being underage, four were from Group B, and two were from Group C. The overall dropout rate was 8.1%. Missing data rates were 8.1% (10/123) for questionnaire-based outcomes and 12.2% (15/123) for physical assessments. Among LvL UP Buddies, 70.5% (55/78) completed both four- and eight-week surveys.

### Baseline characteristics

Table 1 summarises the baseline demographics. The mean age of participants was 35.3 years (SD = 10.3), and 62.6% were female. Most participants were Chinese (92%), employed (75.4%), and college-educated (62.6%). Over half (57.1%) reported household monthly income above SGD 6,000. Regarding digital health experience, 51.6% were current users of health-related digital tools, and 59.0% wore fitness trackers.

**Table 1:** Participant characteristics at baseline

	Total (*n* = 122)	Group A (*n* = 42)	Group B (*n* = 20)	Group C (*n* = 19)	Group D (*n* = 41)
Age (Mean+-SD)	35.31 ± 10.26	38.02 ± 11.23	31.00 ± 8.71	35.14 ± 12.16	34.73 ± 8.34
Sex					
Female	76 (62.3)	25 (59.5)	11 (55.0)	17 (89.5)	23 (56.1)
Male	46 (37.7)	17 (40.5)	9 (45.0)	2 (10.5)	18 (43.9)
Ethnicity					
Chinese	112 (91.8)	38 (90.5)	18 (90.0)	19 (100.0)	37 (90.2)
Indian	7 (5.7)	1 (2.4)	2 (10.0)	0 (0.0)	4 (9.8)
Malay	1 (0.8)	1 (2.4)	0 (0.0)	0 (0.0)	0 (0.0)
Others	2 (1.6)	2 (4.8)	0 (0.0)	0 (0.0)	0 (0.0)
Education					
Secondary/Post secondary	45 (36.9)	20 (47.6)	6 (30.0)	7 (36.8)	12 (29.3)
Bachelor	55 (45.1)	14 (33.3)	9 (45.0)	9 (47.4)	23 (56.1)
Postgraduate	22 (18.0)	8 (19.1)	5 (25.0)	3 (15.8)	6 (14.6)
Employment					
Students/Not employed	30 (24.6)	10 (23.8)	9 (45.0)	4 (21.1)	7 (17.1)
Employed	92 (75.4)	32 (76.2)	11 (55.0)	15 (79.0)	34 (82.9)
Marital status					
Currently married	38 (31.1)	16 (38.1)	4 (20.0)	4 (21.1)	14 (34.2)
Single/Separated/Divorced	84 (68.9)	26 (61.9)	16 (80.0)	15 (79.0)	27 (65.9)
Monthly household income					
< SGD 6000	48 (42.9)	20 (51.3)	2 (11.1)	8 (53.3)	18 (45.0)
SGD 6000-9999	33 (29.5)	12 (30.8)	7 (38.9)	2 (13.3)	12 (30.0)
SGD10000 and above	31 (27.7)	7 (18.0)	9 (50.0)	5 (33.3)	10 (25.0)
Wearable use					
Not currently using	50 (41.0)	17 (40.5)	10 (50.0)	4 (21.1)	19 (46.3)
Currently using	72 (59.0)	25 (59.5)	10 (50.0)	15 (79.0)	22 (53.7)
Health Program Use					
Not currently using	59 (48.4)	16 (38.1)	14 (70.0)	8 (42.1)	21 (51.2)
Currently using	63 (51.6)	26 (61.9)	6 (30.0)	11 (57.9)	20 (48.8)

Group A: Intervention arm responders who used the LvL UP app with a LvL UP Buddy for 8 weeks

Group B: Intervention arm non-responders who used the LvL UP app with a LvL UP Buddy for 8 weeks

Group C: Intervention arm non-responders who used the LvL UP app with a LvL UP Buddy during the first stage (weeks 1–4) and received additional human coaching sessions during the second stage (weeks 5–8) of the intervention

Group D: Control arm participants. e Of the 123 enrolled participants, one was excluded for being underage

Baseline differences were observed across intervention subgroups, reflecting self-selection into responder and non-responder trajectories. Group A tended to be older. Group C had a higher proportion of female participants (Table 1), and reported lower baseline mental well-being and higher psychological distress than Group B (Table 2).

**Table 2:** Mental and behavioural outcomes at baseline and eight-week follow-up assessments

Outcomes	Group A		Group B		Group C		Group D	
	Baseline (*n* = 42)	Eight-week (*n* = 42)	Baseline (*n* = 20)	Eight-week (*n* = 16)	Baseline (*n* = 19)	Eight-week (*n* = 17)	Baseline (*n* = 41)	Eight-week (*n* = 38)
Mental well-being (score)	47.55 ± 8.13	50.19 ± 7.91	47.70 ± 8.45	48.94 ± 10.46	45.37 ± 7.60	44.94 ± 5.54	47.63 ± 8.26	48.29 ± 7.53
Psychological distress (score)	6.05 ± 4.39	4.79 ± 3.57	6.35 ± 3.88	5.88 ± 4.53	7.05 ± 3.98	6.47 ± 3.50	4.85 ± 3.91	4.66 ± 3.95
Sleep duration (hours/day)	6.63 ± 1.27	6.93 ± 1.14	6.45 ± 0.81	6.66 ± 0.77	6.42 ± 1.07	6.51 ± 1.06	6.71 ± 1.09	6.53 ± 0.85
MVPA (minutes/week)	60.00 (0–120.00)	97.50 (0–340.00)	37.50 (0–295.00)	60.00 (0–290.00)	150.00 (0–360.00)	60.00 (0–280.00)	20.00 (0–180.00)	75.00 (0–, 345.00)
Fruit intake (servings/day)	0.53 (0.23–2.00)	0.56 (0.31–1.44)	0.25 (0.08–0.94)	1.09 (0.30–4.75)	0.80 (0.37–, 4.50)	0.86 (0.53–4.68)	0.86 (0.43–2.03)	0.73 (0.36–2.58)
Vegetable intake (servings/day)	4.82 (0.86–5.86)	3.92 (0.86–5.50)	0.80 (0.40–5.00)	0.68 (0.34–2.07)	4.86 (0.86–5.36)	2.64 (0.86–4.94)	3.00 (0.86–5.21)	2.22 (0.71–5.14)

Group A: Intervention arm responders who used the LvL UP app with a LvL UP Buddy for 8 weeks

Group B: Intervention arm non-responders who used the LvL UP app with a LvL UP Buddy for 8 weeks

Group C: Intervention arm non-responders who used the LvL UP app with a LvL UP Buddy during the first stage (weeks 1–4) and received three additional motivational interviewing-informed sessions with human coaches during the second stage (weeks 5–8) of the intervention

Group D: Control arm participants. MVPA: moderate-to-vigorous physical activity. Data presented in the table as: mean ± SD, or median (Q1–Q3)

### Intervention engagement

**Fig. 4:**
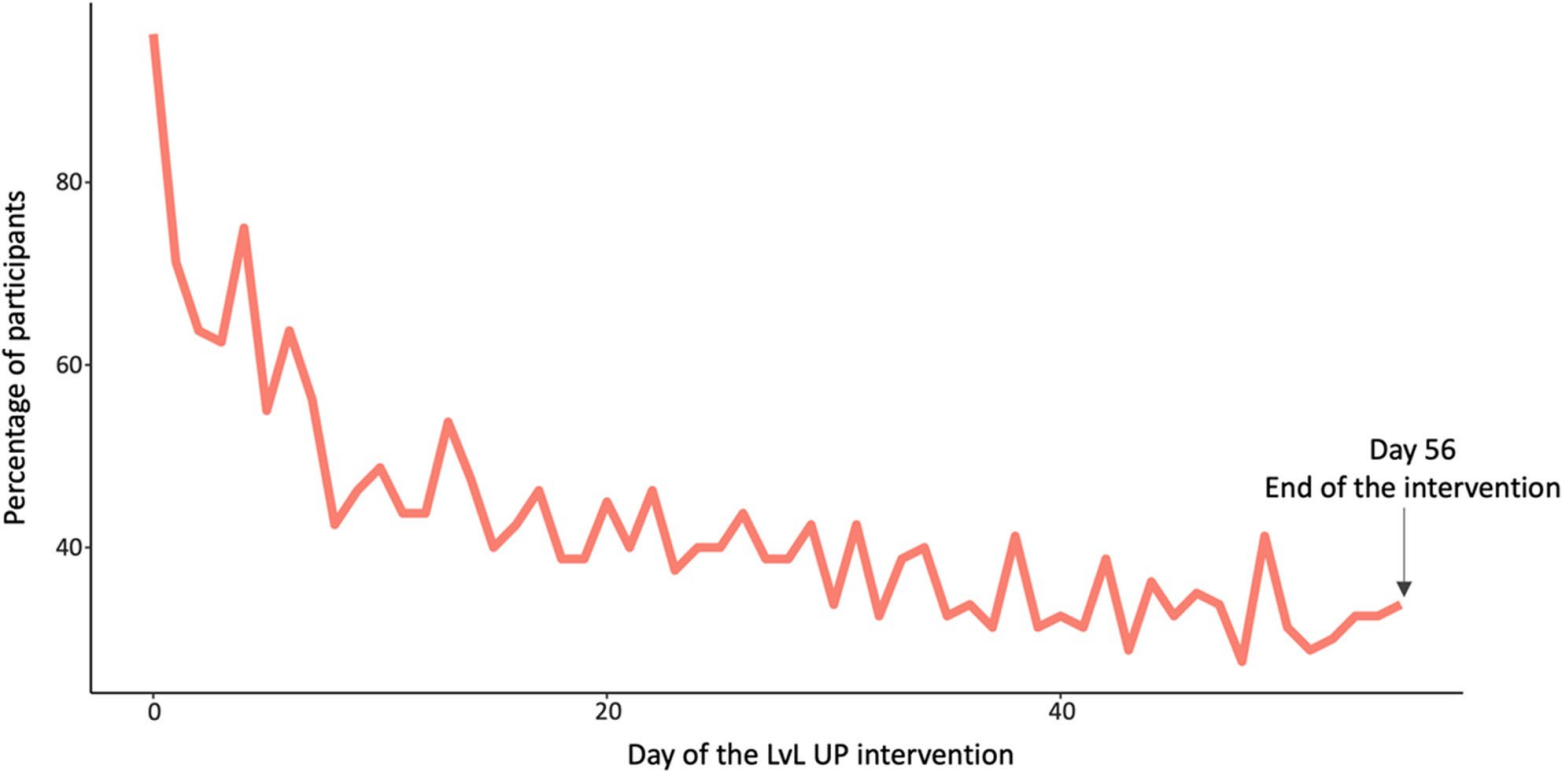
Percentage of participants using the app at least once per day during the intervention period

Figure 4 shows that daily active use dropped approximately 60% during the first 10 days. By day 28 (four weeks), 40% of participants remained active; by day 56 (eight weeks), this decreased to around 30%. On average, participants used the LvL UP app on 24 out of 56 days (SD = 17).

For digital coaching, 39.5% (32/81) completed all 12 sessions as intended, and 70.4% (57/81) completed at least six. Digital coaching was the most frequently used feature (median 11, IQR 4–13), followed by Life Hacks (median 7, IQR 1–30), MakanMemo (median 7, IQR 1–20), Breeze (median 2, IQR 0–11), and Journaling (median 1, IQR 0–4). Technical issues affected data collection for the StepLah.

Among non-responders in Group C, 78.9% (15/19) completed at least one MI-informed coaching session, and 52.6% (10/19) completed all three.

### App usability and satisfaction

The average SUS score for the LvL UP app (*n* = 70) was marginally acceptable at 60.50 (SD = 16.90). The NPS was + 15.6, with 46.7% promoters and 31.1% detractors.

### Change from baseline

GEE analyses showed favourable trends for Strategy #1 (Groups A + B) compared with the control arm (Group D) in mental well-being and psychological distress (Table 3). Both intervention strategies were associated with improvements in sleep duration. Due to the pilot nature of the trial, no adjustments were made for baseline covariates.

**Table 3:** Differences in change from baseline of mental health and behavioural outcomes: two adaptive intervention strategies vs. control arm

Outcomes	Strategy #1 vs. control	Strategy #2 vs. control
	Coefficient (95% CI)	Coefficient (95% CI)
Mental well-being (score)	2.12 (−0.58, 4.82)	0.65 (−1.83, 3.14)
Psychological distress (score)	−0.94 (−2.08, 0.20)	−0.72 (−1.84, 0.40)
Sleep duration (hours/day)	0.49 (0.17, 0.82)	0.40 (0.03, 0.76)
MVPA (minutes/week)	44.50 (−42.9, 131.93)	−16.14 (−121.19, 88.92)
Fruit intake (servings/day)	0.23 (−1.04, 1.50)	−0.22 (−1.48, 1.05)
Vegetable intake (servings/day)	0.01 (−1.63, 1.66)	0.02 (−1.74, 1.79)

Strategy #1: Participants received the LvL UP app, including a LvL UP Buddy, regardless of their response status (Group A + B)

Strategy #2: Participants received the LvL UP app, including a LvL UP Buddy at the first stage of intervention, and received three additional motivational interviewing-informed sessions with human coaches if they were non-responders after 4 weeks (Group A + C). Control: Group D. MVPA: moderate-to-vigorous physical activity

### Progression criteria for proceeding to a definitive trial

As summarised in Table 4, five of the six progression criteria were met; only digital coaching session adherence fell below the target (39.5% vs. target of 70%). No adverse events were reported.

**Table 4:** Progression criteria for proceeding to a definitive trial

Description of the criteria	Target threshold	Observed value	Met
Recruitment during six weeks	≥ 50% target sample	100%	**✓**
Trial retention at 8-week follow-up	≥ 75%	91.9%	**✓**
Non-responder rate	40–60%	46.3%	**✓**
Missing data on primary outcome	≤ 20%	8.1%	**✓**
Adherence to digital coaching sessions	≥ 70%	39.5%	✗
Positive directional change	At least one behavioural or mental health outcome	At least three reported in this study	**✓**

## Discussion

This pilot trial showed the feasibility of delivering and evaluating LvL UP using a SMART design. Key feasibility indicators, including rapid recruitment, high buddy uptake, strong retention, low rates of missing data, and an acceptable non-responder rate, support the intervention's operational viability. Positive trends were observed in mental health and sleep duration, particularly within Strategy #1. Five of the six pre-specified progression criteria were achieved, with the exception of digital coaching adherence. These findings provide valuable guidance for future definitive trials and contribute to the emerging evidence base on adaptive, holistic mHealth interventions.

Recruitment, often a major barrier in mHealth interventions, was completed within six weeks [51–53]. Broad inclusion criteria allowed over 85% of screened individuals to qualify, while an automated portal streamlined screening and scheduling and reduced administrative burden [54]. Consistent with prior findings, targeted email outreach, though smaller in scale, had the highest conversion rates [55]. A tiered reimbursement structure may have further incentivised timely enrolment. Telegram recruitment generated over 80 sign-ups within six hours, which reached a segment of the population highly motivated to engage with research. While this facilitated rapid enrolment, it may also have selected for participants with higher motivation. Future implementation should prioritise community-based recruitment to ensure more representative participation.

For participant retention, the rate exceeded 90% with low rates of missing data, which is higher than that of other mHealth interventions [49, 51, 56–58]. This is noteworthy given the complexity of the intervention. Structured retention strategies likely drove this performance, including flexible survey completion windows, multi-channel reminders, direct communication, and standardised protocols (e.g., staff rehearsal, manuals, and real-time data monitoring and validation) [56, 59]. These practices highlight how structured, participant-centred logistics can improve short-term trial retention and data completion rates.

In SMART designs, non-responder rates between 20% and 80% are considered optimal [58]. Our observed rate of 46.3% was consistent with the sample size assumption. In this pilot, participants were classified as non-responders based on digital coaching session completion and usefulness ratings. While this approach was practical for pilot testing as the data were readily accessible, it was an imperfect proxy for actual intervention response. As a result, some participants engaged consistently without showing measurable change, whereas others used the app infrequently yet still reported improvements in well-being. For the definitive trial, we will refine the non-responder definition to use interim assessments of mental well-being, ensuring that adaptive decisions are outcome-based and aligned with the intervention’s primary outcome.

App engagement followed a typical mHealth pattern, with a steep early decline in daily use followed by a gradual decrease over time [60, 61]. Despite incorporating engagement strategies such as gamification, push notifications, and storytelling, digital coaching adherence remained below the target. Features requiring more active user input, such as Breeze and other self-regulation tools, were used less frequently. This pattern suggests that user engagement may be higher with features that provide more external structure and guidance compared with those relying primarily on user initiative [62]. 

In addition, minimal onboarding guidance (e.g., reliance on printed handouts), lack of clarity regarding expected engagement levels, and several technical issues (e.g., slow chatbot responses, app crashes, and step-tracker synchronisation errors) likely contributed to navigation difficulties and usability friction [23]. Such challenges are common in early-stage mHealth interventions and are associated with disengagement [23, 27, 63, 64]. In our pilot, these issues largely reflected prototype-level constraints, including system complexity, early-stage technical stability, and limited time and resources for real-time troubleshooting and usability optimisation [23]. The next iteration of the LvL UP app will therefore prioritise improvements to chatbot responsiveness, system infrastructure, and in-app guidance to support more consistent, multi-feature engagement.

Our results showed that MI-informed session adherence exceeded that of digital coaching. This aligns with established literature showing greater user engagement with human-delivered support [65–67]. This finding underscores the primary role of the app as the first-line component of the adaptive intervention. It identifies participants for whom low-intensity digital strategies are insufficient, allowing for the targeted and timely escalation to a more intensive, human-delivered approach. Higher adherence to MI-informed sessions also reflects appropriate escalation rather than limited utility of the app. For future development, the goal is not to replace human interaction but to optimise the adaptive pathway so that limited human resources are directed toward individuals who are most likely to benefit.

Exploratory analyses showed improvements in mental health and sleep outcomes among intervention participants. These estimates were consistent with prior studies on the synergistic effects of holistic mHealth interventions [15]. Interestingly, Strategy #1 showed stronger trends than Strategy #2, possibly due to differences in gender composition between Groups B and C, or insufficient intensity of the MI-informed sessions [68–70]. As this was a pilot trial, these findings are preliminary and should be interpreted with caution.

LvL UP contributes to the evolving digital health landscape by operationalising a holistic mHealth coaching model within an adaptive SMART framework. A key strength was the end-to-end implementation of the SMART design, including timely escalation from digital to human-delivered support. The use of a control app that mirrored the intervention interface helped minimise expectation bias and strengthen internal validity. Additionally, the application of a validated, precision-based sample size calculation ensured efficient use of resources while providing a strong foundation for a future definitive trial. Beyond informing the next phase, these findings have broader implications for developing scalable, person-centred mHealth interventions aligned with public health goals for preventive care.

Based on the findings of this pilot, several practical lessons can guide future implementation of adaptive mHealth interventions. Efficient recruitment can be improved by streamlining eligibility assessment using automated online screening tools while ensuring alignment with the target population. Moving beyond research-oriented channels towards community-based recruitment will also be important for achieving a more representative sample. To address the high attrition that is common in digital health, trial retention can be strengthened through structured, participant-centred operational strategies such as flexible survey windows, multi-channel reminders, and proactive data-quality monitoring. Sustained engagement will depend on prioritising technical stability and usability before introducing more complex interactive features. Finally, future implementations should carefully consider appropriate decision points and tailoring variables that balance participant needs with operational feasibility to optimise adaptive intervention delivery.

### Limitations

This trial has several limitations. The sample was relatively young, highly educated, and primarily recruited from a Telegram research group, which may limit the generalisability of the findings, as it reflects early adopters who are highly motivated to engage in digital health research. As a result, the engagement and retention rates observed here may represent a “best-case” scenario rather than what might be expected in broader community settings. While ethically approved, the incentive structure may have also inflated short-term enrolment and retention. Finally, the use of sealed opaque envelopes for allocation concealment carries a small risk of sequential errors or unintentional unblinding, although strict procedures were followed to minimise this.

## Conclusions

This pilot trial demonstrated the feasibility of implementing LvL UP 3.0, a holistic mHealth lifestyle coaching intervention with adaptive support, within a SMART design. While further refinements are needed to improve digital engagement, the findings provide strong operational guidance and a foundation for a larger-scale trial to evaluate long-term effectiveness and cost-effectiveness.

## Supplementary Information

Supplementary Material 1.

Supplementary Material 2.

Supplementary Material 3.

Supplementary Material 4.

## Data Availability

The datasets generated and/or analysed during the current study are not publicly available due to privacy or other restrictions but are available from the corresponding author on reasonable request.

## Abbreviations

NCDs: Non-Communicable Diseases
CMDs: Common Mental Disorders
mHealth: Mobile Health
WHO: World Health Organization
SMART: Sequential Multiple Assignment Randomised Trial
CONSORT: Consolidated Standards of Reporting Trials
NUS: National University of Singapore
MI: Motivational Interviewing
WEMWBS: Warwick-Edinburgh Mental Well-Being Scale
K6: 6-Item Kessler Psychological Distress Scale
MVPA: Moderate-to-Vigorous Intensity Physical Activity
SUS: System Usability Scale
NPS: Net Promoter Score
SD: Standard Deviation
IQR: Interquartile Range
GEE: Generalised Estimating Equations
