# Supplementary material for: Feasibility of the “LvL UP” trial: a pilot sequential multiple assignment randomised trial of an adaptive, holistic mHealth lifestyle coaching intervention
Source: Int J Behav Nutr Phys Act. 2026 Jan 3;23:16. doi: 10.1186/s12966-025-01869-7 (PMC12931026; doi:10.1186/s12966-025-01869-7)
Supplement: Supplementary file 2 — Supplementary Material 2. [file 12966_2025_1869_MOESM2_ESM.pdf]

Contemporary Clinical Trials 150 (2025) 107833

Contents lists available at ScienceDirect

Contemporary Clinical Trials

journal homepage: www.elsevier.com/locate/conclintrial

The LvL UP trial: Protocol for a sequential, multiple assignment, randomised controlled trial to assess the effectiveness of a blended mobile lifestyle intervention

Oscar Castro [a,*,1], Jacqueline Louise Mair [a,b,1], Shenglin Zheng [a,b], Sarah Yi Xuan Tan [a,b], Ahmad Ishqi Jabir [a], Xiaoxi Yan [c], Bibhas Chakraborty [c,d,e], E Shyong Tai [a,b,f], Rob M. van Dam [b,g], Florian von Wangenheim [a,h], Elgar Fleisch [a,i,h], Konstadina Griva [j,2], Tobias Kowatsch [a,k,l,h,2], Falk Müller-Riemenschneider [b,m,2]

[a] Future Health Technologies, Singapore-ETH Centre, Campus for Research Excellence and Technological Enterprise (CREATE), Singapore
[b] Saw Swee Hock School of Public Health, National University of Singapore, Singapore
[c] Centre for Quantitative Medicine, Duke-NUS Medical School, Singapore
[d] Department of Statistics and Data Science, National University of Singapore, Singapore
[e] Department of Biostatistics and Bioinformatics, Duke University, Durham, NC, USA
[f] Yong Loo Lin School of Medicine, National University of Singapore, Singapore
[g] Department of Exercise and Nutrition Sciences, Milken Institute School of Public Health, George Washington University, Washington, DC, USA
[h] Centre for Digital Health Interventions, Department of Management, Technology, and Economics, ETH Zurich, Zurich, Switzerland
[i] Centre for Digital Health Interventions, Institute of Technology Management, University of St. Gallen, St. Gallen, Switzerland
[j] Office of Research, Lee Kong Chian School of Medicine, Nanyang Technological University, Singapore
[k] Centre for Digital Health Interventions, Institute for Implementation Science in Health Care, University of Zurich, Zurich, Switzerland
[l] Centre for Digital Health Interventions, School of Medicine, University of St. Gallen, St. Gallen, Switzerland
[m] Digital Health Center, Berlin Institute of Health, Charité-Universitätsmedizin Berlin, Berlin, Germany

ARTICLE INFO

Keywords:
Digital health
Stepped care
Depression
Diabetes
Mental health
Health behaviour change
Implementation science

ABSTRACT

Background: Blended mobile health (mHealth) interventions – combining self-guided and human support components – could play a major role in preventing non-communicable diseases (NCDs) and common mental disorders (CMDs). This protocol describes a sequential, multiple assignment, randomised trial aimed at (i) evaluating the effectiveness and cost-effectiveness of LvL UP, an mHealth lifestyle intervention for the prevention of NCDs and CMDs, and (ii) establishing the optimal blended approach in LvL UP that balances effective personalised lifestyle support with scalability.
Methods: LvL UP is a 6-month mHealth holistic intervention targeting physical activity, diet, and emotional regulation. In this trial, young and middle-aged Singaporean adults at risk of developing NCDs or CMDs will be randomly allocated to one of two initial conditions ('LvL UP' or 'comparison'). After 4 weeks, participants categorised as non-responders from the LvL UP group will be re-randomised into second-stage conditions: (i) continuing with the initial intervention (LvL UP) or (ii) additional motivational interviewing (MI) support sessions by trained health coaches (LvL UP + adaptive MI). The primary outcome is mental well-being. Secondary outcomes include anthropometric measurements, resting blood pressure, blood metabolic profile, health status, and health behaviours (physical activity, diet). Outcomes will be measured at baseline, 6 months (post-intervention), and 12 months (follow-up).
Discussion: In addition to evaluating the effectiveness of LvL UP, the proposed study design will contribute to increasing evidence on how to introduce human support in mHealth interventions to maximise their effectiveness while remaining scalable.
Trial registration: The LvL UP Pilot trial was prospectively registered with ClinicalTrials.gov (NCT06360029).

* Corresponding author.
E-mail address: oscar.castro@sec.ethz.ch (O. Castro).
[1] These authors share first authorship.
[2] These authors share senior authorship.

https://doi.org/10.1016/j.cct.2025.107833
Received 2 September 2024; Received in revised form 8 January 2025; Accepted 31 January 2025
Available online 1 February 2025
1551-7144/© 2025 The Authors. Published by Elsevier Inc. This is an open access article under the CC BY-NC license (http://creativecommons.org/licenses/by-nc/4.0/).

1. Background

1.1. Background and rationale

Non-communicable diseases (NCDs), such as cardiovascular disease, diabetes, or cancer, and common mental disorders (CMDs), such as depression or anxiety, represent the primary causes of death and disability worldwide, causing major health and financial burdens [1–3]. Lifestyle behaviours, including physical activity, diet, tobacco smoking, alcohol consumption, sleep, stress and emotional regulation, are important modifiable risk factors associated with the prevention and management of both NCDs [4] and CMDs [5]. Yet, successful lifestyle behaviour change and maintenance is often challenging and only implemented by a fraction of those in need.

In recent years, the growing popularity of smartphones and wearable devices has catalysed the development of new mobile health (mHealth) interventions designed to help people manage their health across a range of different lifestyle domains [6–8]. MHealth interventions can be broadly categorised into self-guided (i.e., delivered remotely with no human involvement) or blended interventions (i.e., those that combine self-guided components with human support).

Self-guided mHealth interventions hold promise for the promotion of lifestyle behaviour at the population level, with the potential to reach more individuals than ever before, but they also face challenges such as poor adherence [9–11], and evidence for their ability to achieve significant change remains inconclusive [12,13]. Blended interventions sit between self-guided and face-to-face approaches, intending to retain the positive aspects associated with both while mitigating the disadvantages [14]. Human support is often demanded by users of mHealth interventions [15,16] and it has been shown to increase effectiveness and engagement over purely self-guided interventions [17–19]. However, introducing human support impacts the intervention's scalability and cost-effectiveness.

Existing blended mHealth interventions usually engage highly trained and specialised professionals to deliver support (e.g., psychologists or certified nutritionists). In addition, many blended mHealth interventions assist in a one-size-fits-all manner, offering support for all users regardless of their progress throughout the intervention [20]. By contrast, different reviews highlight that individual user response to mHealth interventions varies drastically [21–23]. This heterogeneity makes stepped-care strategies particularly interesting in the context of mHealth interventions. Stepped care is an adaptive approach that saves resources by initiating the intervention with minimal support (i.e., by first providing a relatively low-cost or low-burden intervention) and stepping up (i.e., offering more expensive or intensive intervention component) only to those who show signs of suboptimal response [24].

In summary, while blended interventions are a promising mHealth approach that combines the strengths of both self-guided and face-to-face interventions, they are typically implemented in a way that neglects intervention's scalability and costs. Evidence is lacking on whether less resource-intensive (e.g., leveraging on non-specialised coaches targeting multiple domains) and personalised (i.e., adaptive) blended mHealth interventions can promote enhanced outcomes and retention, while ensuring a rational resource allocation.

LvL UP is a mHealth intervention aimed at preventing NCDs and CMDs in multi-ethnic Southeast Asian populations [25]. The intervention has been developed to facilitate the inclusion of an adaptive blended component for 'non-responders' (e.g., users that show no improvements in the outcomes of interest or are at risk of disengaging). A formal evaluation of LvL UP's effectiveness and cost-effectiveness is pending, including the implementation of the adaptive blended component.

1.2. Objectives

Primary objective: To compare the effects of LvL UP versus a comparison condition for both primary (mental well-being) and secondary outcomes. Using the subgroup letters in Fig. 1, this objective will involve the following comparison: (A + B) versus D. We hypothese that the change from baseline to 6 months for both primary (mental well-being) and secondary outcomes will be superior for participants allocated to the LvL UP condition.

Secondary objective I: To compare the effects of a version of LvL UP which incorporates an adaptive blended component consisting of motivational interviewing (MI) support delivered to non-responders (LvL UP + adaptive MI) versus a comparison condition for both primary (mental well-being) and secondary outcomes. This objective will involve the following comparison: (A + C) versus D. We hypothese that the change from baseline to 6 months for both primary (mental well-being) and secondary outcomes will be superior for participants allocated to the LvL UP + adaptive MI condition.

Secondary objective II: To compare the effects of LvL UP versus LvL UP + adaptive MI conditions for both primary (mental well-being) and secondary outcomes. This objective will involve the following comparison: (A + B) versus (A + C). We hypothesise that the change from baseline to 6 months for both primary (mental well-being) and

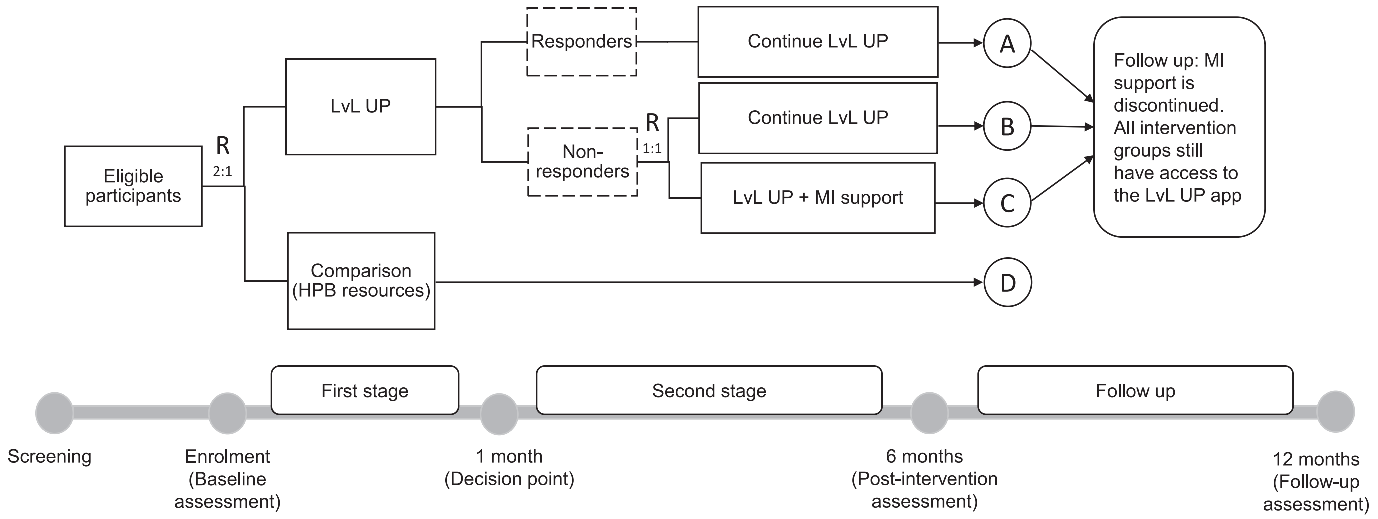

Fig. 1. Sequential Multiple Assignment Randomisation Trial (SMART) design for LvL UP. Note: letters A-D represent four possible pathways throughout the trial for eligible participants; R = Randomisation ratio; MI = Motivational Interviewing; HPB = Health Promotion Board.

secondary outcomes will be superior for participants allocated to the LvL UP + adaptive MI condition.

Exploratory objectives: This study also has four exploratory objectives. The first is to identify the most cost-effective intervention condition from the healthcare and societal perspectives. The second is to explore time-varying and baseline moderators on intervention outcomes. The third is to investigate maintenance by assessing the intervention outcomes at follow-up (12 months after baseline). The fourth is to conduct a theory-driven process evaluation informed by the UK's Medical Research Council guidelines [26], exploring mechanisms of action (e.g., self-efficacy), context, and implementation.

1.3. *Trial design*

The research design is a Sequential Multiple Assignment Randomised Trial (SMART) [27,28]. This design (Fig. 1) will allow us to test an adaptive intervention that starts with the LvL UP app and then introduces MI support for 'non-responders' (e.g., those that show no improvements or are at risk of disengaging), as well as to compare the results of both intervention approaches with the comparison group [29].

Regarding the tailoring variable(s) (i.e., the variables used to determine how intervention delivery should be adapted based on user characteristics or response to initial treatment [30]), we will use the LvL UP pilot trial results (see below) to inform the tailoring variable(s) for the main trial, with a focus on the observed responders/non-responders' ratio for different variables. We will consider engagement and app evaluation variables (e.g., number of app components completed over the first 4 weeks, net promoter score), preliminary intervention effects (e.g., initial positive response), or a combination of the two.

1.3.1. *LvL UP pilot trial*

The research plan includes a pilot before the main LvL UP trial to help estimate the intervention's effect size and level of engagement, responders/non-responders' ratio for different tailoring variables, attrition, percentage of missing data, recruitment capabilities, and the overall feasibility of the research protocol (i.e., are there any changes required for the main trial?). This pilot will replicate the main LvL UP trial, with the only differences being reduced duration (8 weeks) and assessment points (i.e., no follow-up measure will be included).

As an exploratory tailoring variable for the LvL UP pilot trial, we will use the number of conversational agent-delivered coaching sessions completed and their average coaching session ratings at week 4 to determine non-response. However, the above tailoring variable might be refined for the main trial based on the pilot trial results regarding the responders/non-responders' ratio.

2. Methods

Ethical approval was obtained from the National University of Singapore (NUS-IRB-2023-421), ETH Zurich (EK-2024-N-13-A), and Nanyang Technological University (NTU-IRB-2024-305) Institutional Review Boards. The LvL UP pilot trial was prospectively registered with ClinicalTrials.gov (NCT06360029) on 7 April 2024. The main LvL UP trial will also be registered at ClinicalTrials.gov before enrolling the first participant. The present protocol was written according to the Standard Protocol Items: Recommendations for Interventional Trials (SPIRIT) [31,32]. A completed SPIRIT checklist is available as an online supplementary material (file 1).

2.1. *Study setting*

The LvL UP intervention has been developed to target a multi-ethnic Asian population (largely Chinese, Indian, and Malay) of young and middle-aged adults at risk of developing NCDs or CMDs in Singapore.

2.2. *Eligibility criteria*

Participants will be eligible if they are: (i) aged 21 to 59 years, (ii) Singapore citizens or permanent residents, (iii) planning to reside in Singapore during the study, (iv) proficient in English, (v) owners of a smartphone (minimum requirements: iOS version 12.4 and Android version 8) with internet access, (vi) able to provide informed consent, and (viii) identified as 'at risk' of developing NCDs and/or CMDs. Participants with a pre-existing condition (e.g., diagnosed depression or diabetes) and/or pregnant will be excluded. Last, participants will not be able to join the study if they are taking part in another lifestyle intervention study.

To identify those 'at risk' of developing NCDs or CMDs, an eligibility survey will assess five different risk factors (physical inactivity, unhealthy diet, poor mental well-being, family history of health conditions, and being overweight or obese) and generate a composite risk factor score ranging from 0 (lowest risk) to 6 (highest risk). These risk factors were selected based on evidence and public health guidelines [33–35] for the prevention of NCDs and/or CMDs, as well as considering existing screening tools in Singapore [36]. Table 1 includes the scoring for each risk factor, together with decision rules to be eligible for inclusion in the trial. It is worth noting that the present composite risk factor score is exploratory and may be revised based on the LvL UP pilot trial results (e.g., by changing the scoring or focusing on one specific risk factor).

2.3. *Intervention and comparison*

2.3.1. *Intervention: LvL UP app*

LvL UP is a holistic mHealth intervention that aims to support young and middle-aged adults in preventing the onset of NCDs and CMDs (i.e., primary prevention). The LvL UP app's first version (LvL UP 1.0) was developed throughout 2021–2023 [25]. Systematic literature reviews [10,37], market analyses [38,39], and user-centred studies [16,40] were conducted to lay the groundwork for its development.

Table 1
Definitions and scoring of risk factors used for eligibility screening.

Factor	Categories	Definitions	Score*
Diet	Optimal	Modified Short Food Frequency Questionnaire's score = 2	0
	Suboptimal	Modified Short Food Frequency Questionnaire's score < 2	1
Physical activity	Optimal	Meeting physical activity guidelines based on a self-reported item	0
	Suboptimal	Not meeting physical activity guidelines based on a self-reported item	1
Mental health	Optimal	Patient Health Questionnaire-4's (PHQ-4) score < 3 for either the anxiety or depression subscales	0
	Suboptimal	Patient Health Questionnaire-4's (PHQ-4) score ≥ 3 for either the anxiety or depression subscales	2
BMI	Optimal	Body mass index <23	0
	Suboptimal	Body mass index ≥23	1
Family history (health conditions)	Optimal	Not having a close relative (parent, sibling and/or child) with a diagnosed health or mental health condition	0
	Suboptimal	Having a close relative (parent, sibling and/or child) with a diagnosed health or mental health condition	1

* Eligible participants are those that score ≥ 2. Participants deemed 'suboptimal' in the mental health domain receive a higher risk score. They will be automatically deemed eligible to compensate for the higher prevalence of physical health-related factors in the screening tool.

The LvL UP 1.0 app has been tested in a feasibility trial to assess the technical viability of, and user satisfaction with, the intervention. Results from the feasibility trial (to be published in a separate paper) highlighted that LvL UP is technically viable, with most users agreeing the app was enjoyable and provided useful and comprehensive information. However, the feasibility trial and our formative studies also highlighted the need to improve certain aspects of the intervention (e.g., usability, user interface and design, shortening the dialogue length and offering more varied answer options) and add further components (i.e., self-regulation tools, human support). These findings have been used to iteratively refine the intervention in two stages: a second version (LvL UP 2.0) that is currently being used in an randomised controlled trial targeting Asian women with a history of gestational diabetes [41] and featured the inclusion of self-regulation tools; and a third version (LvL UP 3.0) that will be used in the present study featuring the LvL UP 2.0 version plus the addition of human support components (LvL UP buddy and adaptive MI).

Briefly, the current LvL UP 3.0 version (referred throughout the paper as LvL UP) includes four key lifestyle intervention components centred around the three core pillars, Move More (physical activity), Eat Well (healthy nutrition), and Stress Less (emotional regulation and mental well-being), as follows: (i) conversational agent-delivered health literacy and psychoeducational coaching sessions, (ii) daily "Life Hacks" (healthy habit suggestions), (iii) self-regulation Tools including a step-based activity tracker, a food diary, and journal and (iv) gamified slow-paced breathing training (Breeze) [42]. These components are delivered using an innovative engagement approach that combines storytelling, MI, just-in-time adaptive notifications, and gamification. As part of the LvL UP app onboarding, participants are asked to nominate a 'LvL UP Buddy' (e.g., a friend, family member, or spouse) to provide additional support online (e.g., WhatsApp messenger) and/or in real life. Screenshots of the LvL UP app are available as an online supplementary material (File 2).

LvL UP is intended to be used intensively for 6 months (24 weeks), with the completion of weekly conversational agent-delivered coaching sessions, daily use of Tools and "Life Hacks", and regular provision of social support by LvL UP Buddies. However, after this intensive intervention phase, the app features and conversational agent-delivered coaching sessions could be used indefinitely.

2.3.2. Adaptive intervention: MI-informed support

Since LvL UP's content is framed around MI – a collaborative, goal-oriented style of communication about change to strengthen user's own motivation to improve their behaviour [43] – we considered that an adaptive intervention component consisting of additional MI-informed sessions with human coaches could complement the digital content of LvL UP. This will provide an opportunity to implement MI strategies in a more comprehensive manner, particularly for those who do not respond to the initial intervention and might need additional support.

The MI-informed sessions for non-responders will consist of six sessions (two biweekly and four monthly) delivered via WhatsApp messenger, lasting between 30 and 40 min. Trained research staff will deliver the sessions aimed at evoking and strengthening motivation and confidence for the lifestyle pillars in LvL UP. More specifically, the content of support will include MI-based strategies and strategic use of communication skills (open-ended questions, reflections, affirmations and summaries) as guided by participants' readiness levels and the flow across MI tasks (engagement; focusing; evoking; planning) [43]. The research staff will receive MI training (2-day course on MI fundamentals) and coaching sessions / role playing from certified MI trainers.

2.3.3. Comparison (usual care)

Participants randomised to the comparison condition will receive standard lifestyle resources (available as supplementary material 3). This includes physical activity, diet and mental well-being content extracted from existing public domain resources developed by the Health Promotion Board (HPB). Established in 2001, the HPB is a government organisation under the Ministry of Health committed to promoting healthy living in Singapore. HPB-developed resources were selected as the comparator because they are the 'go-to', nation-wide health resources in Singapore which cover LvL UP's pillars.

2.4. Procedures and participant flow

The flow of participants through the study is described in Fig. 1. Individuals interested in participating in the study will complete an online eligibility survey to determine eligibility. Eligible participants will be asked to book an appointment for a first in-person study visit, where they will sign the participant consent form in the presence of a witness and undergo the baseline assessments. Both outcome assessors and data analysts will be blinded to the group allocation. Other members of the research team as well as trial participants will not be blinded to the group allocation. Once the trial is completed, the study team will disseminate the results via publications, conferences, progress reports to funding bodies, and registration in recognised public registries.

2.4.1. Group allocation

Participants will be given computer-generated (Stata/SE 16.0) random allocations to one of the two initial conditions (LvL UP or comparison) following consent and baseline assessment. The computer-generated allocations will be performed by a member of the research team who will not take part in the outcome assessments nor participant enrolment. Randomisation will follow a 2:1 ratio favouring the LvL UP group to increase the statistical power for the secondary objectives while ensuring adequate statistical power for the primary objective. At four weeks (decision point), participants from the LvL UP group will be classified as responders or non-responders based on pre-specified criteria. Non-responder participants will be re-randomised with equal probability (1:1) to one of the two second-stage conditions: (i) continuing with the initial intervention (LvL UP) or (ii) additional MI-informed sessions (LvL UP + adaptive MI). At the end of month 6, MI-informed and Buddy support will be discontinued but all participants from the LvL UP group will be able to use the app for another 6 months (maintenance phase).

2.4.2. Recruitment

Participants will be recruited through multiple channels, including social media (e.g., Facebook, X, Instagram), flyers, community groups, snowballing, and research registries. Participants will be compensated for the multiple assessments, with the possibility of a total of SG$300 (≈US$221) in compensation.

2.5. Measures

2.5.1. Eligibility survey

The eligibility survey to be used during screening will include a set of questions on (i) mental health status via the Patient Health Questionnaire-4 [44], (ii) nutritional intake via a Modified 2-item Food Frequency Questionnaire, (iii) physical activity via a single-item question [45], (iv) Body Mass Index (BMI) via self-reported weight and height, and (v) family history of health conditions. In addition, participants will be asked about their medication use, whether they have been diagnosed with NCDs and/or CMDs (e.g., diabetes or depression), and other items as per the selection criteria.

2.5.2. Trial outcomes

Eligible participants will be assessed on-site at baseline and after 6 months (post-intervention / primary endpoint). In addition, a subset of measures will be carried out remotely (online) during months 1 and 3 (intermediate assessments) and during months 9 and 12 (follow-up assessments). Each of the measures and their specific timing is detailed in Table 2. Further details on the specific instruments are available as an

online supplementary material (4). Considering LvL UP is a holistic intervention targeting body and mind, we decided to define mental well-being (assessed with the Warwick-Edinburgh Mental Well-being Scale) as the primary outcome. The literature indicates mental well-being is influenced by all three domains targeted by LvL UP: physical activity [46], diet [47,48] and emotional regulation / mental health [49].

2.5.3. Process evaluation

A process evaluation will be conducted in line with the UK's Medical Research Council Guidance on process evaluations for complex interventions [26]. Methods will entail a mixture of qualitative and quantitative approaches, including surveys, interviews, direct observation, and app-based passively collected data. The aim of the process evaluation is to explore implementation, mechanisms of action, and contextual factors that may affect implementation and intervention outcomes. A separate protocol will describe the process evaluation in detail.

2.5.4. Health economic evaluation

The primary outcome measure for the cost-effectiveness analysis will be the EuroQol 5 Dimension 5 Level (EQ-5D-5L) questionnaire [50], from both the healthcare and societal perspectives. This includes intervention costs (i.e., staff and materials cost), participant costs (i.e., the value of the participant's time to take part in the intervention), work productivity [51] and healthcare utilisation (including hospitalisation, outpatient care, and primary care) [52]. We will follow the UK's National Institute for Health and Care Excellence guidance for economic evaluations of public health interventions [53] and will report the outcomes of the evaluation in line with the Consolidated Health Economic Evaluation Reporting Standards (CHEERS) statement [54].

2.6. Statistical analyses

All statistical analyses will be performed using the R statistical software and follow the intention-to-treat principle. The primary outcome measure for the trial is the mental well-being rating (via the Warwick-Edinburgh Mental Wellbeing Scale) from baseline to 6 months (primary endpoint). We are also interested in the outcome measures change from baseline to 12 months for the exploratory analyses.

2.6.1. Primary and secondary aim analyses

To assess the effects of LvL UP (A + B in Fig. 1) versus comparison (D) and LvL UP + adaptive MI (A + C) versus comparison (D), Generalized Estimating Equations (GEE) with robust variance estimations will be used. The weight and replicate method [55] will be used in the GEE models to simultaneously estimate the embedded conditions (A + B) and (A + C) in the models. Inverse probability weighting will be used to account for the over or under-representation of the outcomes due to the randomisation scheme, and observations will be replicated as they are used in multiple comparisons. For example, responders to stage 1 intervention (A) will be replicated. The GEE models will include the trial condition (A + B, A + C, D), time (at baseline and 6 months), and the condition-by-time interaction. The condition-by-time interaction effect will determine if the change from baseline to 6 months for LvL UP and

Table 2
The schedule of enrolment, interventions, and assessments.

	Enrolment	Random-isation (Baseline)*	1-month	3-month	6-month*	9-month	12-month
**Enrolment**							
Eligibility screening	X						
Informed consent	X						
Allocate for stage 1 (all)		X					
Reallocate for stage 2 (non-responders)			X				
**Intervention**							
A, B (LvL UP)		X	X	X	X	X	X
C (LvL UP + MI)			X	X	X	X	X
D (comparison)		X	X	X	X	X	X
**Assessment (eligibility)**							
Mental health (Patient Health Questionnaire-4)	X						
Physical activity (single self-reported item)	X						
Anthropometry (self-reported BMI)	X						
Health conditions	X						
Medication use	X						
Diet (Modified 2-item Food Frequency Questionnaire)	X						
**Assessment (main trial)**							
Mental well-being (Warwick-Edinburgh Mental Well-being Scale-14)		X	X	X	X	X	X
Generic well-being (WHO-5)		X	X	X	X	X	X
Mental health - depression (Patient Health Questionnaire-9)		X	X	X	X	X	X
Mental health - stress (Kessler Psychological Distress Scale)		X	X	X	X	X	X
Self-reported health		X		X	X	X	X
Change in health status		X		X	X	X	X
Sociodemographic characteristics		X					
Use of digital health technologies		X			X	X	X
Diet (Diet screener)		X	X	X	X	X	X
Quality of life (EuroQol 5 Dimension 5 Level)		X		X	X	X	X
Physical activity (International Physical Activity Questionnaire-long)		X	X	X	X	X	X
Physical activity (steps – continuously monitored)		X	X	X	X	X	X
Sleep (Pittsburgh Sleep Quality Index)		X			X		X
Smoking & alcohol consumption		X			X		X
Healthcare utilisation		X		X	X	X	X
Work productivity		X		X	X	X	X
Medication use					X		X
Anthropometry (self-reported BMI)			X	X		X	X
Anthropometry (BMI, waist circumference, hip circumference, body composition)		X			X		
Resting blood pressure		X			X		
Blood metabolic profile		X			X		

* Assessments will be carried out in-person. The rest of the assessments will be conducted via online surveys.

LvL UP + adaptive MI differ from the comparison condition. Sensitivity analyses will be conducted by adjusting the models according to the baseline covariates identified (via bivariate and multivariate models) that affect the probabilities of missing data and response (stage 1).

To determine whether the adaptive MI component for non-responders to LvL UP is effective, the same GEE models will be used, as it also allows for the comparison of (A + B) versus (A + C).

2.6.2. Exploratory aims analyses

The first exploratory aim is to identify the most cost-effective among the three trial conditions embedded in the trial from healthcare and societal perspectives. We will compare the costs and outcomes of the three intervention conditions – LvL UP (A + B in Fig. 1) versus comparison (D) and LvL UP + adaptive MI (A + C) versus comparison (D) – using the net benefit regression framework [56]. The results of this analysis will be expressed as an incremental net benefit for each group. Regression analysis will be employed to adjust for potential confounders, and clustered standard errors will be calculated using sandwich variance estimators [57] to account for the correlated outcomes. Uncertainty in cost-effectiveness findings within the study timeframe will be characterised using a cost-effectiveness acceptability curve and a 95 % confidence interval. Additionally, subgroup analyses, such as by sex, socio-economic group, and multimorbidity, will be explored. The second exploratory aim is to explore baseline moderators that impact the intervention outcomes at stages 1 and 2. This will be done with Q-learning [58], which allows for the investigation of the intervention allocation options with the baseline and time-varying moderators in combination with the embedded tailoring variable. The third exploratory aim is to investigate if the effect is sustained for 12 months after the baseline. We will again use the GEE models with the weight and replicate method, to compare the change in outcomes from baseline to 12 months. The fourth exploratory aim will follow the UK's Medical Research Council guidelines for process evaluation [26], where more descriptive statistics will be done apart from the aforementioned modelling.

2.6.3. Pilot trial analyses

The recruitment rate, attrition, and adherence rates at stages 1 and 2 by trial conditions, and the response rate at stage 1 will be calculated. Attrition and adherence will be associated with the baseline variables and compared across conditions. GEE models with the weight and replicate method will also be fitted to estimate the intervention effect sizes. These values will then be used to inform if the sample size for the main trial is sufficient and if the operational aspects of the trial need to be adjusted.

2.6.4. Missing data

We will inspect the missing data patterns by comparing the baseline variables between participants with and without missing data within each randomised group. Baseline variables found to be related to the missingness will be further evaluated for their relationship with the outcomes using participants with completed data at each follow-up. These covariates may be included when modelling the main effects in the main analyses. Multiple imputations using time-ordered nested conditional imputation models [59] will be used if the assumption that the missingness follows a random mechanism is valid [60]. Sensitivity analyses will then compare results from models using complete case data and data with multiple imputations, and with and without covariates adjustments.

2.6.5. Sample size

Power calculations were based on methods described by Oetting et al. [61]. They were powered to compare the baseline to 6 months change in mental well-being between LvL UP (A + B) versus the comparison condition (primary objective). We assume the effect size (Cohen's d) for well-being to be 0.3 and the responder rate to stage 1 intervention (LvL UP) to be 50 %. The effect size was estimated by aggregating data from five meta-analyses on mHealth interventions and their reported impact on participant's mental well-being [62–66]. To obtain a marginal power of at least 80 % with a two-tailed Type I error rate of 5 % for each outcome, 458 participants will be required; we further buffer for 73 % retention following a recent meta-analysis estimate for digital health interventions lasting >8 weeks [67], rounding the number for a final sample size of 650. Therefore, 217 participants will be randomised to the comparison condition, and 433 participants will be randomised to start with stage 1 intervention (LvL UP), of which we estimate about 217 participants (50 % non-responders) will be re-randomised to either continue with LvL UP or receive MI at stage 2 (LvL UP + adaptive MI). The above sample size calculations for the main trial might be refined based on the pilot trial results.

To formalise the sample size calculations for the pilot trial, the precision-based approach by Yan et al. [68] was used to ensure the estimated outcomes for LvL UP (A + B) and LvL UP + adaptive MI (A + C) are controlled within a certain precision (i.e., the margin of error as a proportion of the outcome's standard deviation). A total of 97 participants will be required, assuming a 50 % responder rate to stage 1 intervention, a two-tailed Type I error rate of 5 % and a precision of 30 %. Taking an 82 % retention estimate for digital health interventions lasting ≤8 weeks [67] and rounding up the number, 120 participants will be recruited. Therefore, 40 and 80 participants will be randomly assigned to the comparison condition and stage 1 intervention (LvL UP), respectively. In addition, an estimated 40 participants in stage 1 intervention will be re-randomised to continue with LvL UP or have the MI at stage 2. Further details on the sample size calculations are available as an online supplementary material (5).

3. Discussion

Promoting a healthy lifestyle is vital for reducing the high incidence of NCDs and CMDs globally [69–71]. Blended mHealth interventions represent a promising strategy for promoting lifestyle behaviours as they can potentially retain the positive aspects of both self-guided and 'traditional' face-to-face (live) approaches. However, implementing current blended mHealth interventions often results in a significant burden and cost, impeding scalability and widespread uptake at the population level.

The LvL UP Trial will take a significant step toward substantiating two critical aspects related to the preservation of resources in blended mHealth interventions. First, it will ascertain whether using a more affordable and scalable alternative to highly specialised and trained supporters (i.e., leveraging on non-specialised coaches with basic MI training addressing different health domains) amplifies LvL UP's intervention effects. Second, it will provide evidence for a more efficient adaptive approach that introduces resource use only when necessary, mirroring how clinicians typically make decisions (i.e., sequentially, after considering a response to the initial intervention).

Apart from testing a more scalable approach to providing human support, by adding a comparison group we will also be able to assess LvL UP's overall effectiveness and cost-effectiveness. Adding a control condition is becoming increasingly popular in SMART designs as it enables to answer multiple research questions, both related to the implementation of specific intervention components as well as the overall trial effectiveness [72–75]. While re-randomising participants into different subgroups certainly increases the target sample size, the study design requires fewer participants overall than if two separate trials were conducted (i.e., optimisation and evaluation).

There are some limitations to the current study that need to be considered. First, although the LvL UP Trial will evaluate intervention response at multiple time points, it will not address whether additional step-ups (e.g., engaging experts or domain-specific coaches) could further optimise the intervention outcomes. Second, constrained by time and funding resources, we cannot assess the intervention's long-term

effects (e.g., 24-months post-intervention initiation). Despite these limitations, the present study will provide foundational evidence on which to build a cost-effective, adaptive intervention strategy for population-level healthy lifestyle promotion.

4. Conclusions

This article described the rationale, aims and design of a study that will be conducted with young and middle-aged adults to inform the implementation and evaluate the effects of a mHealth holistic lifestyle intervention to prevent NCDs and CMDs. The study design and methodology presented here may help guide future intervention developers.

Funding

This project was conducted as part of the Future Health Technologies program (Campus for Research Excellence and Technological Enterprise), which is jointly funded by the National Research Foundation, Prime Minister's Office, Singapore and ETH Zurich, Switzerland.

CRediT authorship contribution statement

Oscar Castro: Writing – review & editing, Writing – original draft, Visualization, Project administration, Methodology, Conceptualization. **Jacqueline Louise Mair:** Writing – review & editing, Project administration, Methodology, Conceptualization. **Shenglin Zheng:** Writing – review & editing, Visualization, Methodology. **Sarah Yi Xuan Tan:** Writing – review & editing, Methodology. **Ahmad Ishqi Jabir:** Writing – review & editing, Methodology. **Xiaoxi Yan:** Writing – review & editing, Methodology. **Bibhas Chakraborty:** Writing – review & editing, Methodology. **E Shyong Tai:** Writing – review & editing, Methodology. **Rob M. van Dam:** Writing – review & editing, Methodology. **Florian von Wangenheim:** Writing – review & editing, Methodology, Funding acquisition. **Elgar Fleisch:** Writing – review & editing, Methodology, Funding acquisition. **Konstadina Griva:** Writing – review & editing, Methodology. **Tobias Kowatsch:** Writing – review & editing, Supervision, Project administration, Methodology. **Falk Müller-Riemenschneider:** Writing – review & editing, Supervision, Project administration, Methodology.

Declaration of competing interest

OC, JM, FvW, EF and TK are affiliated with the Centre for Digital Health Interventions (CDHI), a joint initiative of the Institute for Implementation Science in Health Care at the University of Zurich, the Department of Management, Technology, and Economics at ETH Zurich and the School of Medicine and Institute of Technology Management at the University of St Gallen. CDHI is funded in part by the Swiss health insurer CSS, the Swiss digital health investor MTIP, and the Austrian healthcare provider Mavie Next (UNIQA). EF is and TK was co-founder of Pathmate Technologies, a university spin-off company that creates and delivers digital clinical pathways. However, Pathmate Technologies, CSS, MTIP and Mavie Next were not involved in the design, interpretation, and analysis during the study, or in writing the paper. The remaining authors declare that the research was conducted in the absence of any commercial or financial relationships that could be construed as a potential conflict of interest.

Acknowledgements

The authors would like to thank Akshaye Shenoi, Samarth Negi, Prabhakaran Santhanam, Chang Siang Lim and Zühlke Engineering Pte Ltd. for contributing to the LvL UP app development.

Appendix A. Supplementary data

Supplementary data to this article can be found online at https://doi.org/10.1016/j.cct.2025.107833.

Data availability

No data was used for the research described in the article.

References

[1] R. Martinez, P. Lloyd-Sherlock, P. Soliz, S. Ebrahim, E. Vega, P. Ordunez, et al., Trends in premature avertable mortality from non-communicable diseases for 195 countries and territories, 1990–2017: a population-based study, Lancet Glob. Health 8 (4) (2020) e511-e23.
[2] J. Rehm, K.D. Shield, Global burden of disease and the impact of mental and addictive disorders, Curr. Psychiatry Rep. 21 (2019) 1–7.
[3] D.J. Stein, C. Benjet, O. Gureje, C. Lund, K.M. Scott, V. Poznyak, et al., Integrating mental health with other non-communicable diseases, Bmj (2019) 364.
[4] V. Bellou, L. Belbasis, I. Tzoulaki, E. Evangelou, Risk factors for type 2 diabetes mellitus: an exposure-wide umbrella review of meta-analyses, PLoS One 13 (3) (2018) e0194127.
[5] K.W. Choi, M.B. Stein, K.M. Nishimi, T. Ge, J.R. Coleman, C.-Y. Chen, et al., An exposure-wide and Mendelian randomization approach to identifying modifiable factors for the prevention of depression, Am. J. Psychiatry 177 (10) (2020) 944–954.
[6] L.C. Brewer, K.L. Fortuna, C. Jones, R. Walker, S.N. Hayes, C.A. Patten, et al., Back to the future: achieving health equity through health informatics and digital health, JMIR Mhealth Uhealth 8 (1) (2020) e14512.
[7] N.C. Jacobson, T. Kowatsch, L.A. Marsch, Digital Therapeutics for Mental Health and Addiction: The State of the Science and Vision for the Future, 2022.
[8] R. Shan, S. Sarkar, S.S. Martin, Digital health technology and mobile devices for the management of diabetes mellitus: state of the art, Diabetologia 62 (6) (2019) 877–887.
[9] D. Guertler, C. Vandelanotte, M. Kirwan, M.J. Duncan, Engagement and nonusage attrition with a free physical activity promotion program: the case of 10,000 steps Australia, J. Med. Internet Res. 17 (7) (2015) e176.
[10] R. Jakob, S. Harperink, A.M. Rudolf, E. Fleisch, S. Haug, J.L. Mair, et al., Factors influencing adherence to mHealth apps for prevention or management of noncommunicable diseases: systematic review, J. Med. Internet Res. 24 (5) (2022) e35371.
[11] M. Karekla, O. Kasinopoulos, D.D. Neto, D.D. Ebert, T. Van Daele, T. Nordgreen, et al., Best practices and recommendations for digital interventions to improve engagement and adherence in chronic illness sufferers, Eur. Psychol. 24 (1) (2019) 49–67.
[12] A. Horn, S. Jírů-Hillmann, J. Widmann, F.A. Montellano, J. Salmen, R. Pryss, et al., Systematic review on the effectiveness of mobile health applications on mental health of breast cancer survivors, J. Cancer Surviv. 1-17 (2023).
[13] A. Romeo, S. Edney, R. Plotnikoff, R. Curtis, J. Ryan, I. Sanders, et al., Can smartphone apps increase physical activity? Systematic review and meta-analysis, J. Med. Internet Res. 21 (3) (2019) e12053.
[14] O.C. Keller, A.J. Budney, C.A. Struble, G.W. Teepe, Blending Digital Therapeutics within the Healthcare System. Digital Therapeutics for Mental Health and Addiction, Elsevier, 2023, pp. 45–64.
[15] S.M. Ang, J. Chen, J.H. Liew, J. Johal, Y.Y. Dan, M. Allman-Farinelli, et al., Efficacy of interventions that incorporate mobile apps in facilitating weight loss and health behavior change in the Asian population: systematic review and meta-analysis, J. Med. Internet Res. 23 (11) (2021) e28185.
[16] J.L. Mair, O. Castro, A. Salamanca-Sanabria, B.F. Frese, F. Von Wangenheim, E. S. Tai, et al., Exploring the potential of mobile health interventions to address behavioural risk factors for the prevention of non-communicable diseases in Asian populations: a qualitative study, BMC Public Health 23 (1) (2023) 753.
[17] J. Antoun, H. Itani, N. Alarab, A. Elsehmawy, The effectiveness of combining nonmobile interventions with the use of smartphone apps with various features for weight loss: systematic review and meta-analysis, JMIR Mhealth Uhealth 10 (4) (2022) e35479.
[18] L. Laranjo, D. Ding, B. Heleno, B. Kocaballi, J.C. Quiroz, H.L. Tong, et al., Do smartphone applications and activity trackers increase physical activity in adults? Systematic review, meta-analysis and metaregression, Br. J. Sports Med. 55 (8) (2021) 422–432.
[19] S. Muralidharan, H. Ranjani, R.M. Anjana, Y. Gupta, S. Ambekar, V. Koppikar, et al., Change in cardiometabolic risk factors among Asian Indian adults recruited in a mHealth-based diabetes prevention trial, Digital Health 7 (2021), 20552076211039032.
[20] V. Hohberg, R. Fuchs, M. Gerber, D. Künzler, S. Paganini, O. Faude, Blended care interventions to promote physical activity: a systematic review of randomized controlled trials, Sports Med. Open 8 (1) (2022) 100.
[21] S.B. Goldberg, S.U. Lam, O. Simonsson, J. Torous, S. Sun, Mobile phone-based interventions for mental health: a systematic meta-review of 14 meta-analyses of randomized controlled trials, PLOS Digital Health 1 (1) (2022) e0000002.
[22] S. O'connor, P. Hanlon, C.A. O'donnell, S. Garcia, J. Glanville, F.S. Mair, Understanding factors affecting patient and public engagement and recruitment to

digital health interventions: a systematic review of qualitative studies, BMC Med. Inform. Decis. Mak. 16 (2016) 1–15.
[23] Y. Wang, H. Xue, Y. Huang, L. Huang, D. Zhang, A systematic review of application and effectiveness of mHealth interventions for obesity and diabetes treatment and self-management, Adv. Nutr. 8 (3) (2017) 449–462.
[24] P. Bower, S. Gilbody, Stepped care in psychological therapies: access, effectiveness and efficiency: narrative literature review, Br. J. Psychiatry 186 (1) (2005) 11–17.
[25] O. Castro, J.L. Mair, A. Salamanca-Sanabria, A. Alattas, R. Keller, S. Zheng, et al., Development of "LvL UP 1.0": a smartphone-based, conversational agent-delivered holistic lifestyle intervention for the prevention of non-communicable diseases and common mental disorders, Front. Digital Health 5 (2023) 1039171.
[26] G.F. Moore, S. Audrey, M. Barker, L. Bond, C. Bonell, W. Hardeman, et al., Process evaluation of complex interventions: Medical Research Council guidance, Bmj (2015) 350.
[27] D. Almirall, I. Nahum-Shani, N.E. Sherwood, S.A. Murphy, Introduction to SMART designs for the development of adaptive interventions: with application to weight loss research, Transl. Behav. Med. 4 (3) (2014) 260–274.
[28] L.M. Collins, I. Nahum-Shani, D. Almirall, Optimization of behavioral dynamic treatment regimens based on the sequential, multiple assignment, randomized trial (SMART), Clin. Trials 11 (4) (2014) 426–434.
[29] X. Yan, D.B. Matchar, N. Sivapragasam, J.P. Ansah, A. Goel, B. Chakraborty, Sequential multiple assignment randomized trial (SMART) to identify optimal sequences of telemedicine interventions for improving initiation of insulin therapy: a simulation study, BMC Med. Res. Methodol. 21 (2021) 1–11.
[30] D. Almirall, S.N. Compton, M. Gunlicks-Stoessel, N. Duan, S.A. Murphy, Designing a pilot sequential multiple assignment randomized trial for developing an adaptive treatment strategy, Stat. Med. 31 (17) (2012) 1887–1902.
[31] N.J. Butcher, A. Monsour, E.J. Mew, A.-W. Chan, D. Moher, E. Mayo-Wilson, et al., Guidelines for reporting outcomes in trial protocols: the SPIRIT-outcomes 2022 extension, Jama 328 (23) (2022) 2345–2356.
[32] A.-W. Chan, J.M. Tetzlaff, P.C. Gøtzsche, D.G. Altman, H. Mann, J.A. Berlin, et al., SPIRIT 2013 Explanation and elaboration: guidance for protocols of clinical trials, Bmj (2013) 346.
[33] A. Budreviciute, S. Damiati, D.K. Sabir, K. Onder, P. Schuller-Goetzburg, G. Plakys, et al., Management and prevention strategies for non-communicable diseases (NCDs) and their risk factors, Front. Public Health 8 (2020) 574111.
[34] W. Isaranuwatchai, Y. Teerawattananon, R.A. Archer, A. Luz, M. Sharma, W. Rattanavipapong, et al., Prevention of non-communicable disease: best buys, wasted buys, and contestable buys, Bmj (2020) 368.
[35] S.-Y. Kim, J.-H. Park, M.Y. Lee, K.-S. Oh, D.-W. Shin, Y.-C. Shin, Physical activity and the prevention of depression: a cohort study, Gen. Hosp. Psychiatry 60 (2019) 90–97.
[36] Y.Z. Khow, T.L.Y. Lim, J.S.P. Ng, J. Wu, C.S. Tan, K.S. Chia, et al., Behavioral impact of national health campaigns on healthy lifestyle practices among young adults in Singapore: a cross-sectional study, BMC Public Health 21 (2021) 1–11.
[37] J.L. Mair, A. Salamanca-Sanabria, M. Augsburger, B.F. Frese, S. Abend, R. Jakob, et al., Effective behavior change techniques in digital health interventions for the prevention or management of noncommunicable diseases: an umbrella review, Ann. Behav. Med. 57 (10) (2023) 817–835.
[38] R. Keller, S. Hartmann, G.W. Teepe, K.-M. Lohse, A. Alattas, L. Tudor Car, et al., Digital behavior change interventions for the prevention and management of type 2 diabetes: systematic market analysis, J. Med. Internet Res. 24 (1) (2022) e33348.
[39] A. Salamanca-Sanabria, O. Castro, A. Alattas, G.W. Teepe, K. Leidenberger, E. Fleisch, et al., Top-funded companies offering digital health interventions fo the prevention and treatment of depression: a systematic market analysis, JMIR Preprints (2022) 40754.
[40] A. Salamanca-Sanabria, A.I. Jabir, X. Lin, A. Alattas, A.B. Kocaballi, J. Lee, et al., Exploring the perceptions of mHealth interventions for the prevention of common mental disorders in university students in Singapore: qualitative study, J. Med. Internet Res. 25 (2023) e44542.
[41] A. Salamanca-Sanabria, S.J. Liew, J. Mair, M. De Iorio, Y.D.Y. Ling, M.T. Tint, et al., A holistic lifestyle mobile health intervention for the prevention of type 2 diabetes and common mental disorders in Asian women with a history of gestational diabetes: a randomised control trial with 3-year follow-up protocol, Trials 25 (1) (2024) 443.
[42] Y.X. Lukic, G.W. Teepe, E. Fleisch, T. Kowatsch, Breathing as an input modality in a gameful breathing training app (breeze 2): development and evaluation study, JMIR Serious Games 10 (3) (2022) e39186.
[43] W.R. Miller, S. Rollnick, Motivational Interviewing: Helping People, Guilford press, change, 2012.
[44] J. Stanhope, Patient health questionnaire-4, Occup. Med. 66 (9) (2016) 760–761.
[45] A.E. Bauman, J.A. Richards, Understanding of the single-item physical activity question for population surveillance, J. Phys. Act. Health 19 (10) (2022) 681–686.
[46] D.X. Marquez, S. Aguiñaga, P.M. Vásquez, D.E. Conroy, K.I. Erickson, C. Hillman, et al., A systematic review of physical activity and quality of life and well-being, Transl. Behav. Med. 10 (5) (2020) 1098–1109.
[47] J. Firth, J.E. Gangwisch, A. Borsini, R.E. Wootton, E.A. Mayer, Food and mood: how do diet and nutrition affect mental wellbeing? Bmj 369 (2020).
[48] M. Grajek, K. Krupa-Kotara, A. Białek-Dratwa, K. Sobczyk, M. Grot, O. Kowalski, et al., Nutrition and mental health: a review of current knowledge about the impact of diet on mental health, Front. Nutr. 9 (2022) 943998.
[49] N. Extremera, N. Sánchez-Álvarez, L. Rey, Pathways between ability emotional intelligence and subjective well-being: bridging links through cognitive emotion regulation strategies, Sustainability 12 (5) (2020) 2111.
[50] M. Herdman, C. Gudex, A. Lloyd, M. Janssen, P. Kind, D. Parkin, et al., Development and preliminary testing of the new five-level version of EQ-5D (EQ-5D-5L), Qual. Life Res. 20 (2011) 1727–1736.
[51] K. Hubens, M. Krol, J. Coast, M.F. Drummond, W.B. Brouwer, C.A. Uyl-de Groot, et al., Measurement instruments of productivity loss of paid and unpaid work: a systematic review and assessment of suitability for health economic evaluations from a societal perspective, Value Health 24 (11) (2021) 1686–1699.
[52] J.W. Neo, X. Guo, E. Abdin, J.A. Vaingankar, S.A. Chong, M. Subramaniam, et al., Excess costs of depression among a population-based older adults in Singapore, 2023.
[53] Health NIf, Excellence C, NICE health technology evaluations: The manual, 2022.
[54] D. Husereau, M. Drummond, F. Augustovski, E. de Bekker-Grob, A.H. Briggs, C. Carswell, et al., Consolidated health economic evaluation reporting standards 2022 (CHEERS 2022) statement: updated reporting guidance for health economic evaluations, MDM Policy Pract. 7 (1) (2022), 23814683211061097.
[55] I. Nahum-Shani, M. Qian, D. Almirall, W.E. Pelham, B. Gnagy, G.A. Fabiano, et al., Experimental design and primary data analysis methods for comparing adaptive interventions, Psychol. Methods 17 (4) (2012) 457.
[56] J.S. Hoch, A.H. Briggs, A.R. Willan, Something old, something new, something borrowed, something blue: a framework for the marriage of health econometrics and cost-effectiveness analysis, Health Econ. 11 (5) (2002) 415–430.
[57] P. Diggle, Analysis of Longitudinal Data, Oxford university press, 2002.
[58] I. Nahum-Shani, M. Qian, D. Almirall, W.E. Pelham, B. Gnagy, G.A. Fabiano, et al., Q-learning: a data analysis method for constructing adaptive interventions, Psychol. Methods 17 (4) (2012) 478.
[59] S.M. Shortreed, E. Laber, T. Scott Stroup, J. Pineau, S.A. Murphy, A multiple imputation strategy for sequential multiple assignment randomized trials, Stat. Med. 33 (24) (2014) 4202–4214.
[60] J.C. Jakobsen, C. Gluud, J. Wetterslev, P. Winkel, When and how should multiple imputation be used for handling missing data in randomised clinical trials–a practical guide with flowcharts, BMC Med. Res. Methodol. 17 (2017) 1–10.
[61] A.I. Oetting, J.A. Levy, Statistical Methodology for a SMART Design in the Development of Adaptive Treatment Strategies. Causality and Psychopathology, Oxford University Press, 2011.
[62] B. Chen, T. Yang, L. Xiao, C. Xu, C. Zhu, Effects of mobile mindfulness meditation on the mental health of university students: systematic review and meta-analysis, J. Med. Internet Res. 25 (2023) e39128.
[63] M. Eisenstadt, S. Liverpool, E. Infanti, R.M. Ciuvat, C. Carlsson, Mobile apps that promote emotion regulation, positive mental health, and well-being in the general population: systematic review and meta-analysis, JMIR Mental Health 8 (11) (2021) e31170.
[64] M. Ferrari, S. Allan, C. Arnold, D. Eleftheriadis, M. Alvarez-Jimenez, A. Gumley, et al., Digital interventions for psychological well-being in university students: systematic review and meta-analysis, J. Med. Internet Res. 24 (9) (2022) e39686.
[65] É. Gál, S. Ștefan, I.A. Cristea, The efficacy of mindfulness meditation apps in enhancing users' well-being and mental health related outcomes: a meta-analysis of randomized controlled trials, J. Affect. Disord. 279 (2021) 131–142.
[66] J. Groot, A. MacLellan, M. Butler, E. Todor, M. Zulfiqar, T. Thackrah, et al., The effectiveness of fully automated digital interventions in promoting mental well-being in the general population: systematic review and meta-analysis, JMIR Mental Health 10 (1) (2023) e44658.
[67] A.I. Jabir, X. Lin, L. Martinengo, G. Sharp, Y.-L. Theng, Car L. Tudor, Attrition in conversational agent–delivered mental health interventions: systematic review and meta-analysis, J. Med. Internet Res. 26 (2024) e48168.
[68] X. Yan, P. Ghosh, B. Chakraborty, Sample size calculation based on precision for pilot sequential multiple assignment randomized trial (SMART), Biom. J. 63 (2) (2021) 247–271.
[69] C.P. Benziger, G.A. Roth, A.E. Moran, The global burden of disease study and the preventable burden of NCD, Glob. Heart 11 (4) (2016) 393–397.
[70] A. O'Neil, F.N. Jacka, S.E. Quirk, F. Cocker, C.B. Taylor, B. Oldenburg, et al., A shared framework for the common mental disorders and non-communicable disease: key considerations for disease prevention and control, BMC Psychiatry 15 (2015) 1–6.
[71] S.A. Peters, X. Wang, T.-H. Lam, H.C. Kim, S. Ho, T. Ninomiya, et al., Clustering of risk factors and the risk of incident cardiovascular disease in Asian and Caucasian populations: results from the Asia Pacific cohort studies collaboration, BMJ Open 8 (3) (2018) e019335.
[72] B.D.B. Gonze, R.D.C. Padovani, M.D.S. Simoes, V. Lauria, N.L. Proença, E. F. Sperandio, et al., Use of a smartphone app to increase physical activity levels in insufficiently active adults: feasibility sequential multiple assignment randomized trial (SMART), JMIR Res. Protoc. 9 (10) (2020) e14322.
[73] MdS Morais Pereira Simoes, Gonze B. de Barros, N. Leite Proença, V. Tonon Lauria, V. Demarchi Silva Terra, Padovani R. da Costa, et al., Use of a smartphone app combined with gamification to increase the level of physical activity of adults and older adults: protocol of a sequential multiple assignment randomized trial, Trials 20 (2019) 1–8.
[74] M.E. Patrick, G.R. Lyden, N. Morrell, C.J. Mehus, M. Gunlicks-Stoessel, C.M. Lee, et al., Main outcomes of M-bridge: a sequential multiple assignment randomized trial (SMART) for developing an adaptive preventive intervention for college drinking, J. Consult. Clin. Psychol. 89 (7) (2021) 601.
[75] G. Wyatt, R. Lehto, P. Guha-Niyogi, S. Brewer, D. Victorson, T. Pace, et al., Reflexology and meditative practices for symptom management among people with cancer: results from a sequential multiple assignment randomized trial, Res. Nurs. Health 44 (5) (2021) 796–810.
