# Supplementary material for: Feasibility of the “LvL UP” trial: a pilot sequential multiple assignment randomised trial of an adaptive, holistic mHealth lifestyle coaching intervention
Source: Int J Behav Nutr Phys Act. 2026 Jan 3;23:16. doi: 10.1186/s12966-025-01869-7 (PMC12931026; doi:10.1186/s12966-025-01869-7)
Supplement: Supplementary file 3 — Supplementary Material 3. [file 12966_2025_1869_MOESM3_ESM.pdf]

SINGAPORE PHYSICAL ACTIVITY GUIDELINES (SPAG)

START MOVING, MAKE EVERY MOVE COUNT

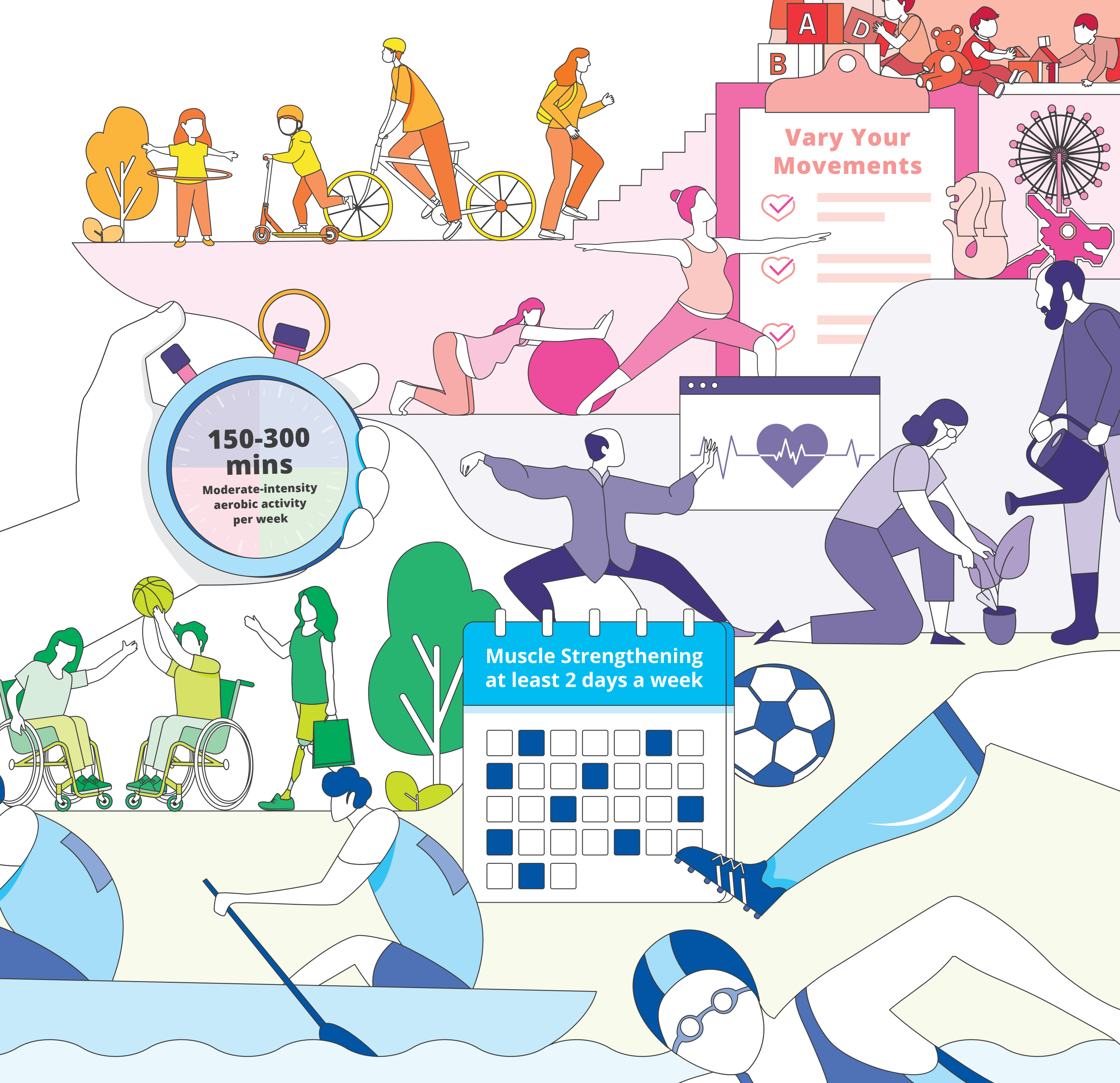

Revised Edition 2022

CONTENTS

1. ACKNOWLEDGEMENTS 1

2. FOREWORD 2

3. BACKGROUND AND OBJECTIVES 3

4. EXECUTIVE SUMMARY 6

5. INTRODUCTION TO THE BENEFITS OF PHYSICAL ACTIVITY 7

6. EXERCISE SAFETY 13

7. PHYSICAL HEALTH STATUS AND ACTIVITY LEVELS IN SINGAPORE 14

8. GUIDELINES BY SUB POPULATION 17
   - PRESCHOOL, SCHOOL CHILDREN AND YOUTHS
   - ADULTS
   - PREGNANT AND POSTPARTUM WOMEN
   - PERSONS WITH DISABILITIES
   - OLDER ADULTS

9. GLOSSARY OF TERMS 45

10. APPENDIX 49

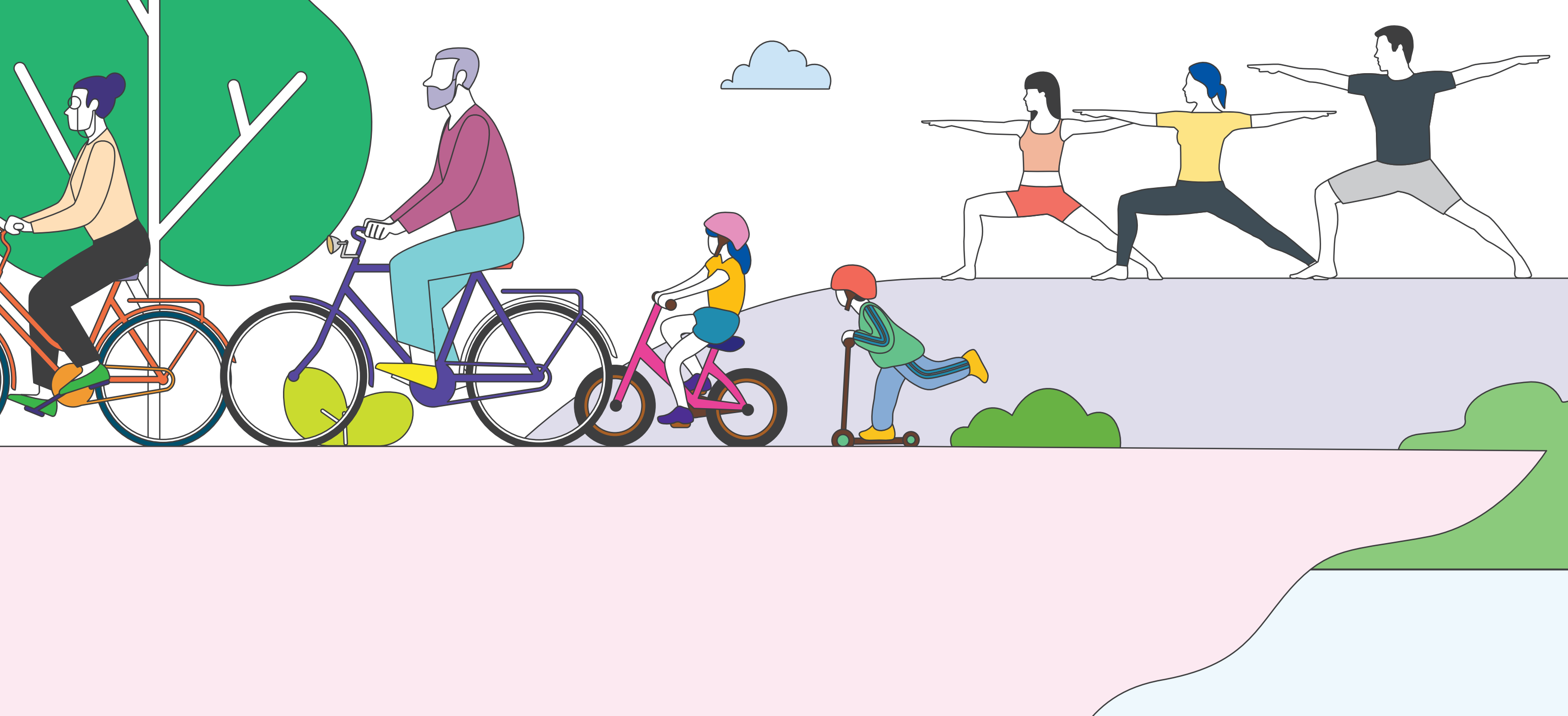

ACKNOWLEDGEMENTS

Health Promotion Board and Sport Singapore would like to acknowledge the support and contributions of the following individuals and organisations in the development of the Singapore Physical Activity Guidelines 2022.

We would first like to thank the team who spearheaded the development of these guidelines - Dr Chiang Hock Woon (DCE, Sport Singapore) and Mr Koh Peng Keng (COO, Health Promotion Board), who chaired the working group; Dr Aaron Sim, Ms Amy Chong, Mr Kenneth McGeough, Mr Kenneth Ng, Ms Lee Eer Ling, Mr Muhammad Amir Hamzah Bin Mohamed Pauz, and Dr Shannon Chia, who served as members of the secretariat.

We would also like to thank the members of the expert advisory committee for the development of the guidelines. They include, Dr Alexander Gorny (Singapore Armed Forces, Head Medical Officer of Centre of Excellence for Soldier Performance), Dr Benedict Tan, Head, SingHealth Duke-NUS Sport & Exercise Medicine Centre, Dr Benny Loo (KK Women's and Children's Hospital, Consultant at Sport and Exercise Medicine Service), Ms Goy Soon Lan (Republic Polytechnic, Director of School of Sports, Health & Leisure), Dr Jason Lee (National University of Singapore, Co-Director of Human Potential Translational Research Programme), Dr Jeyakumar Henry (A*STAR, Director of Clinical Nutrition Research Centre), Dr Joanne Yoong (National University of Singapore, Associate Professor at Behavioural and Implementation Science Interventions Center), Mr Jonathan Tan (Ministry of Education, Deputy Director of Physical, Sports and Outdoor Education Branch), Dr Koh Koon Teck (National Institute of Education, Head of Physical Education and Sports Science), Dr Lim Wee Shiong (Tan Tock Seng Hospital, Senior Consultant of Department of Geriatric Medicine), Dr Lucy Quek (National Institute of Early Childhood Development, Dean of Curriculum & Programmes), Dr Marcus Lee (Singapore Sports Institute, Head of Sports Science & Medical Centre), Mr Michael Lim (ReMark Group, Assistant Director, Health & Wellness), Dr Mueller-Riemenschneider Falk (Saw Swee Hock School of Public Health, Programme Leader of Physical Activity and Nutrition Determinants in Asia), Dr Nikki Lim-Ashworth (Institute of Mental Health, Senior Clinical Psychologist and Research Fellow of Department of Child and Adolescent Psychiatry), Ms Pearly Seah (People's Association, Director of Youth & Sports Division), Mr Steven Tay (Ministry of Education, Deputy Director of Physical, Sports & Outdoor Education Branch), and Dr Yeo Tee Joo (National University Heart Centre, Director of Cardiac Rehabilitation Unit).

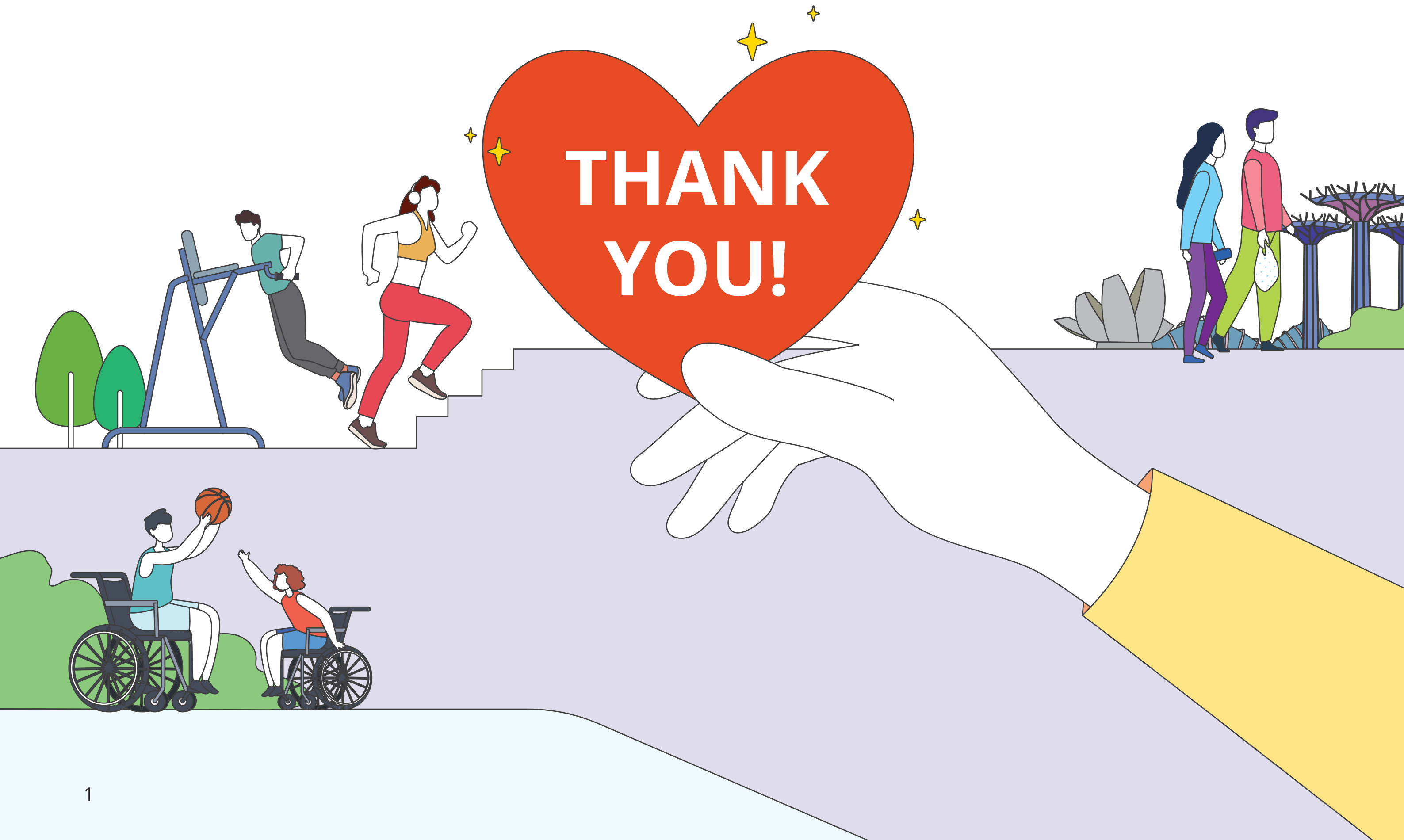

FOREWORD

Regular physical activity offers multifarious benefits to all ages. It impacts the physical health in many ways, including reducing the risk of chronic diseases as well as building stronger bones and muscles for functional fitness. It also helps improve one's mental and emotional well-being by reducing stress and anxiety.

Since the national physical activity guidelines for adults and for children and youth were published in 2011 and 2013 respectively, new evidence has progressively emerged in the last decade and has been adopted by the World Health Organisation (WHO) through the publication of the *WHO Guidelines on physical activity*, *sedentary behaviour and sleep for children under 5 years of age* in 2019 and the *WHO Guidelines on physical activity and sedentary behaviour* in 2020.

To support Singaporeans in keeping active in ways that are relevant to them, the Singapore Physical Activity Guidelines (SPAG) 2022 adopts the WHO guidelines and sets out recommendations on physical activity and reducing sedentary behaviour for different population segments, cutting across life stages – from children to adults and older adults. Through these recommendations, we hope to make active lifestyles attainable for everyone.

We would like to extend our appreciation to the SPAG advisory committee for developing the guidelines with us. These guidelines serve as a national reference for many different stakeholders – medical professionals, educators, employers, fitness professionals and community leaders – who play a part in promoting physical activity among our population. The guidelines are also meant for the general community; we hope they will leverage the recommendations to equip themselves with the knowledge and apply the simple, practical tips to embark on a more active lifestyle.

By offering a useful guide to all Singaporeans to live better through sport and physical activity, we will pave the way for a more active and healthier nation.

Lim Teck Yin,
CEO, Sport Singapore

Koh Peng Keng,
COO, Health Promotion Board

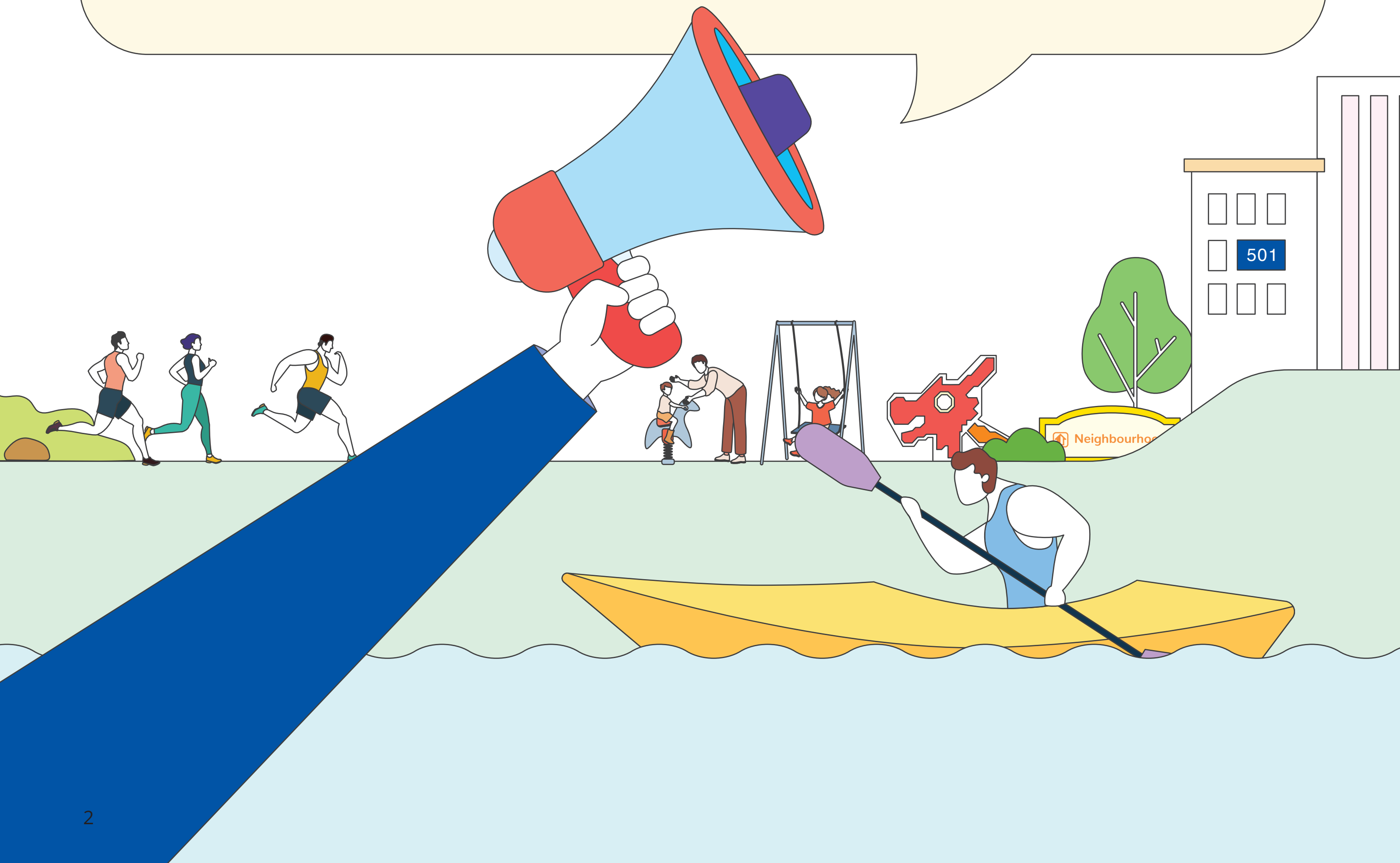

SPAG OBJECTIVES

Increase awareness of the health benefits associated with meeting the recommendations, such as better physical and mental health and social cohesion

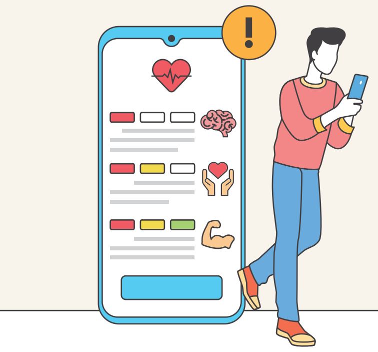

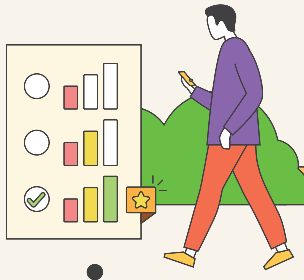

Provide recommendations on achieving sufficient levels of physical activity and reduction of sedentary behaviour suitable for different sub-populations

Inspire Singaporeans to take first steps to own their health through tips and examples of real-world scenarios

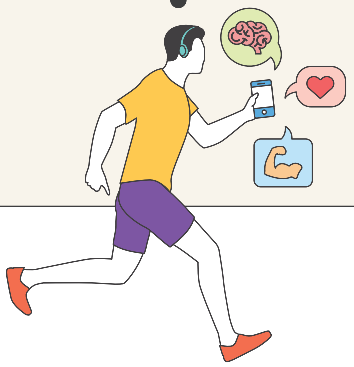

3-STAGE JOURNEY OF SPAG

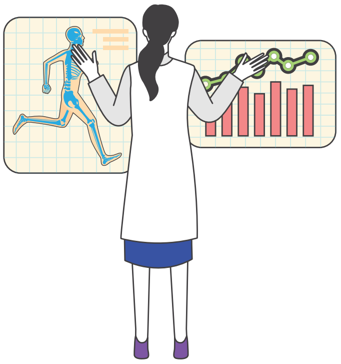

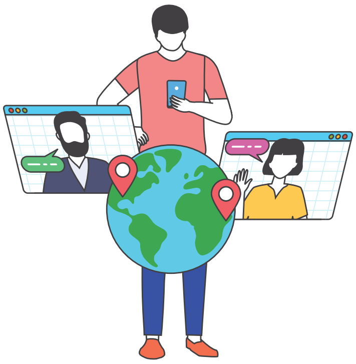

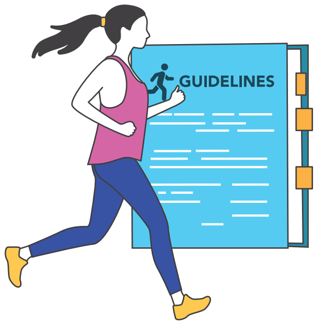

ADOPT

Adopted best practices and scientific evidence from World Health Organisation (WHO) 2020 Guidelines on Physical Activity & Sedentary Behaviour

ADAPT

Consulted local and international physical activity and public health experts to adapt global recommendations and contextualise them for the Singapore population

ACT

- Published a set of guidelines useful to all Singaporeans, including the general community
- Developed action plans to sustain efforts for communicating recommendations and encourage Singaporeans to lead an active lifestyle

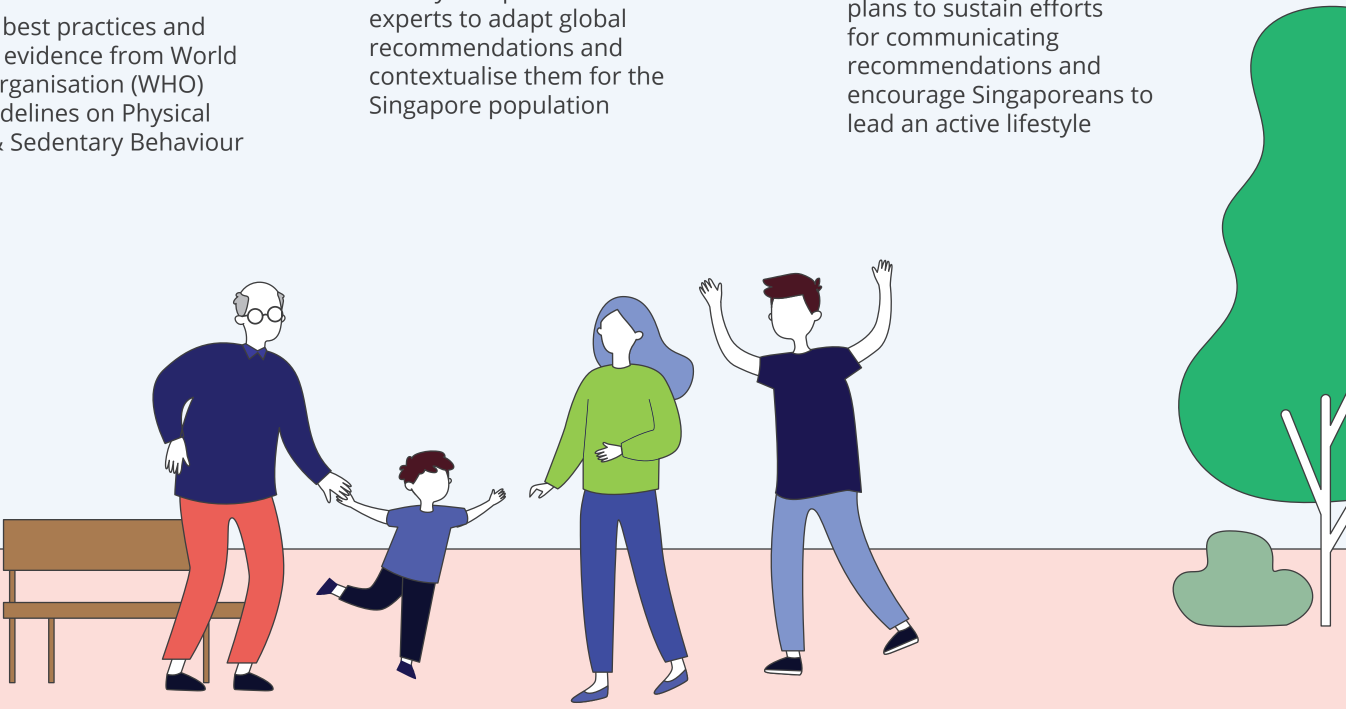

FACTORS CONTRIBUTING TO THE NEED FOR UPDATES IN SINGAPORE'S GUIDELINES

EVOLVING TRENDS AND EMERGING EVIDENCE IN PHYSICAL ACTIVITY

Lifestyle trends and physical activity research has continued to evolve over the years

- ✔ Previous guidelines were launched in 2011 for adults and in 2013 for children and adolescents
- ✔ It is timely and strategic to provide new recommendations and best practices to keep up with emerging evidence and meet current lifestyle trends
- ✔ New set of guidelines is expanded to be inclusive of segments such as pregnant and post-partum women, individuals with chronic diseases and individuals with disabilities
- ✔ There is also an added emphasis on recommendations to break up and reduce sedentary behaviour

ALIGNMENT WITH WHO'S PLANS

The WHO has set targets to tackle global physical inactivity

- ✔ Launch of the Global Action Plan on Physical Activity (GAPPA) in 2018, with the vision to have more active people and a healthier world by reducing physical inactivity
- ✔ Updates to Singapore's guidelines can contribute to achieving the shared goals and vision of GAPPA

SINGAPORE PHYSICAL ACTIVITY GUIDELINES AS A KEY TOOL FOR PROMOTING PUBLIC HEALTH

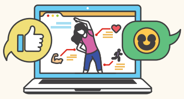

IMPROVE PUBLIC AWARENESS

Educate the general local community on physical activity guidelines and provide practical tips to build towards an active lifestyle.

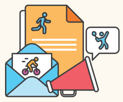

CALL TO ACTIONS

Influence policy actions or implementation of programmes that can support or promote a culture of active living.

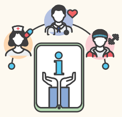

SUPPORT HEALTHCARE PROFESSIONALS

Inform and support local health and fitness professionals with evidence-based recommendations.

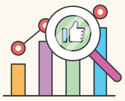

SET STANDARDIZED BENCHMARKS

Standardize benchmarks for monitoring progress of the nation towards meeting physical activity recommendations.

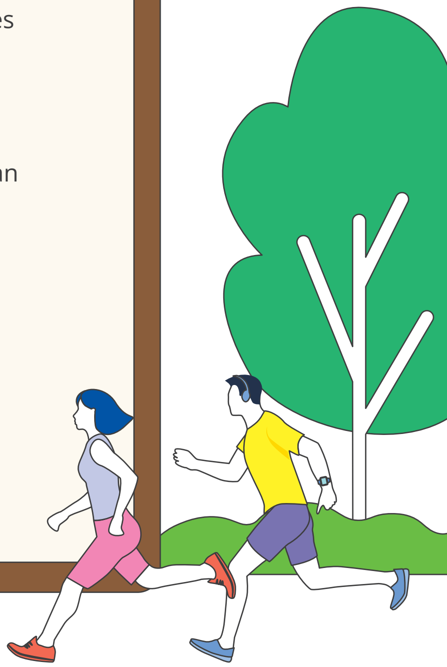

TARGET AUDIENCE

PUBLIC / GENERAL COMMUNITY

- Infographics with simple and positive language that is easily digestible to drive behavioural change
- Applying practical tips into daily life and build towards an active lifestyle, one step at a time regardless of life stage and level of readiness

PRACTITIONERS

- Consumer-friendly information that is evidence-based and ready to be used without the need for redesign
- Encourage ground-up initiatives for the community by adopting the guidelines and adapt to suit the needs of target audiences

POLICYMAKERS

- Evidence-based recommendations which have been adapted to the Singapore context
- Support efforts in promoting an active living culture in Singapore through policies and regulations, including influencing the built environment and social norming active behaviours

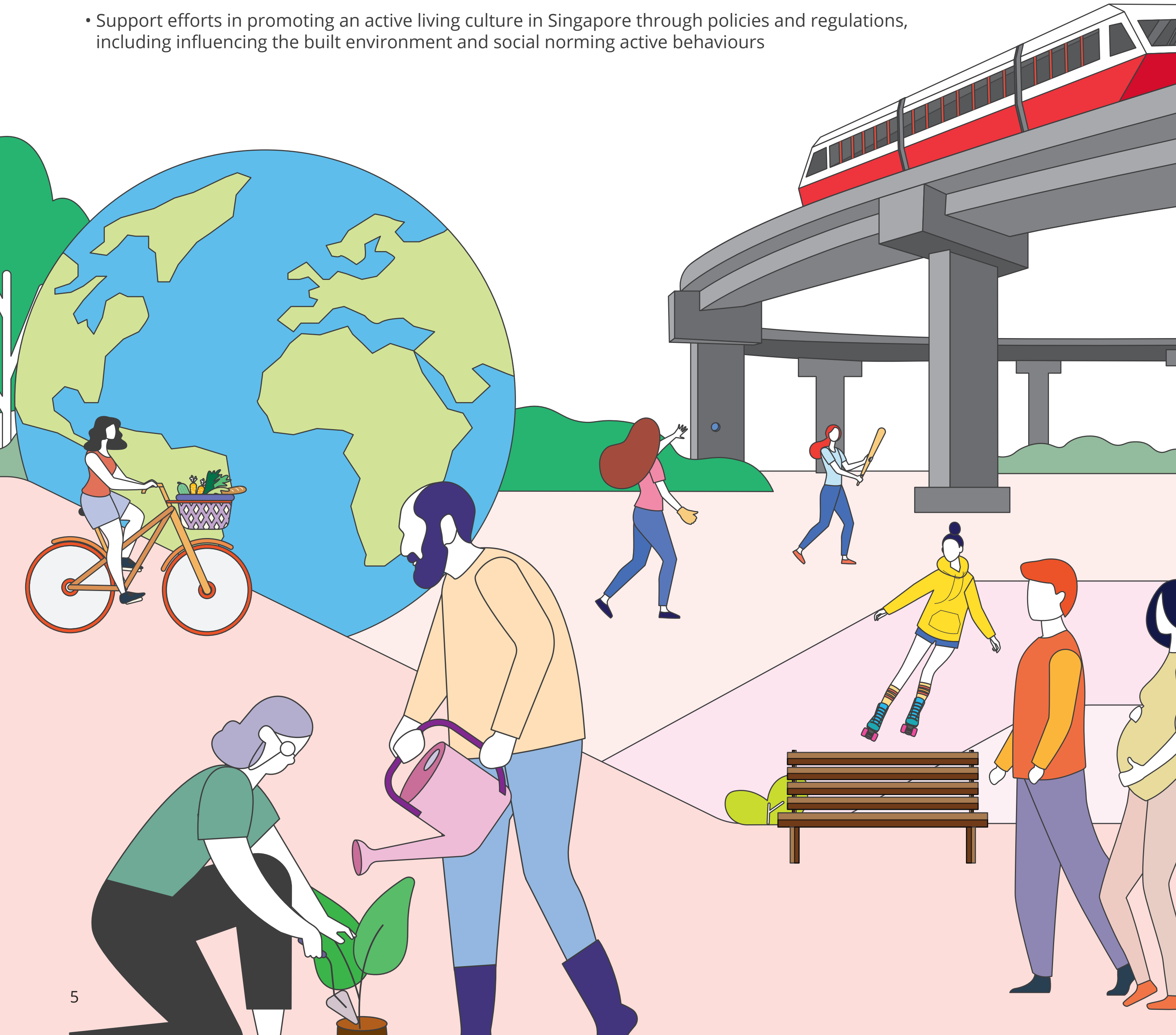

EXECUTIVE SUMMARY

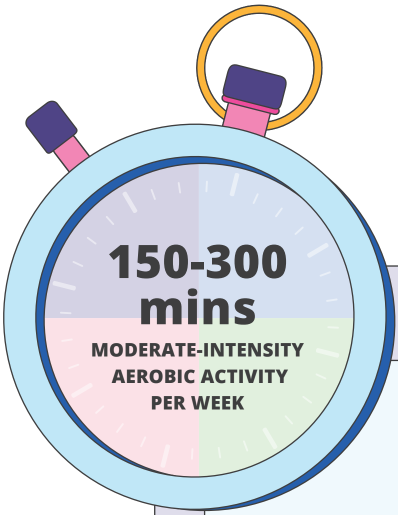

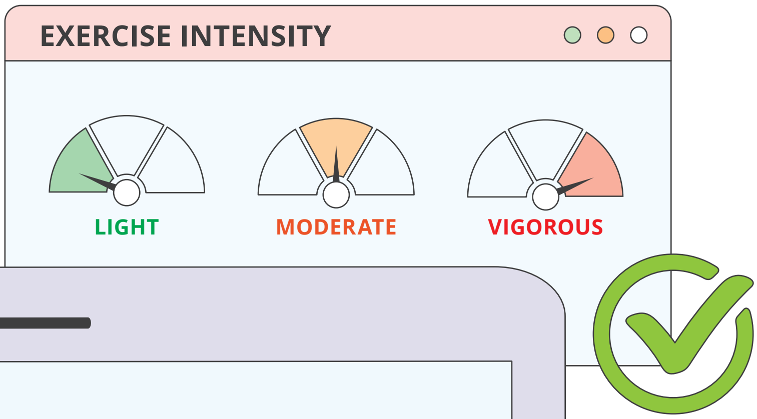

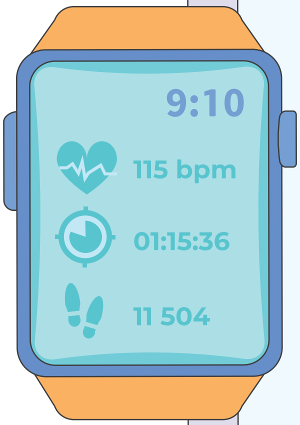

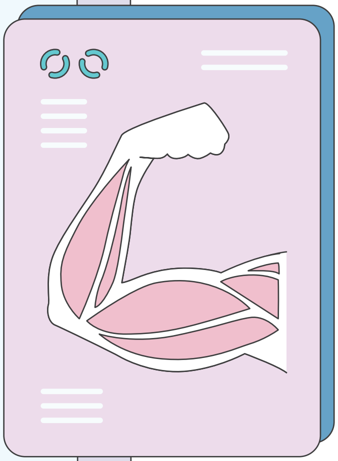

The WHO defines physical activity as any bodily movement produced by skeletal muscles that requires energy expenditure and recommends both moderate- and vigorous-intensity physical activity for an improved health. Sedentary behaviour is defined as any waking behaviour while in a sitting, reclining, or lying posture with low energy expenditure. Importantly, large amounts of sedentary behaviour are directly associated with poorer health outcomes.

Adopting the best practices from the WHO Guidelines on Physical Activity and Sedentary Behaviour, SPAG seeks to provide public health recommendations for children, adults, and older adults on the amount of physical activity (frequency, intensity, and duration) required to achieve significant health benefits and mitigate health risks.

The guidelines are intended for policymakers, practitioners, and the public. The final recommendations presented are for all populations and age groups ranging from preschool to 65 years and older and are relevant to people of all abilities. Those with chronic medical conditions and/or disability, pregnant, and postpartum women should try to meet the recommendations where possible.

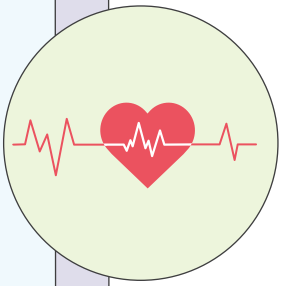

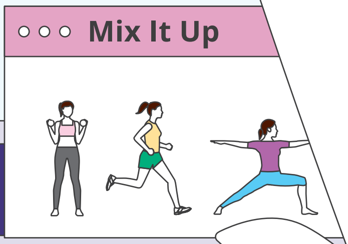

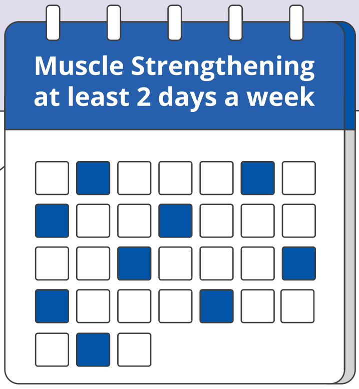

THE BENEFITS OF PHYSICAL ACTIVITY

Physical activity is defined as “any bodily movement produced by the contraction of skeletal muscles that increase energy expenditure above a resting level”. Physical activity can broadly be classified into three intensities – light, moderate and vigorous. When performing physical activity at a light-intensity, an individual should be able to talk and sing while moving. At a moderate-intensity, talking is comfortable, but singing becomes more difficult. At a vigorous-intensity, neither singing or prolonged talking is possible.

EXERCISE INTENSITY

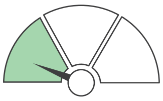

Light
Can talk in full sentences and sing

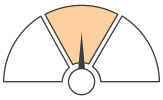

Moderate
Can talk in phrases or short sentences but cannot sing

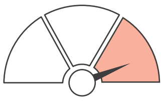

Vigorous
Have difficulty talking

5 TYPES OF ACTIVITIES FOR HEALTH AND PERFORMANCE

AEROBIC

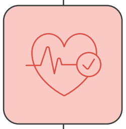

Activity that increases heartbeat and effort needed to breathe

Benefit:
Improves cardiovascular fitness

BONE STRENGTH

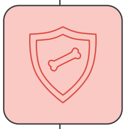

Activity that produces a force on the bones

Benefit:
Promotes bone growth and strength

MUSCULAR STRENGTH

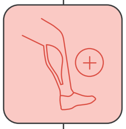

Activity that requires the body’s muscles to work or hold against an applied force or weight

Benefit:
Increases skeletal muscle strength, power, endurance, and mass

FLEXIBILITY

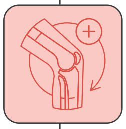

Activity that causes the body’s muscles to be lengthened but in a controlled manner

Benefit:
Enhances ability of a joint to move through its full range of motion

BALANCE

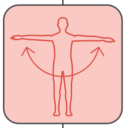

Activity that develops the ability to stay in control of the body when stationary or moving (causes the body to produce a coordinated response, e.g. sensory, strength & flexibility)

Benefit:
Improves motor skills

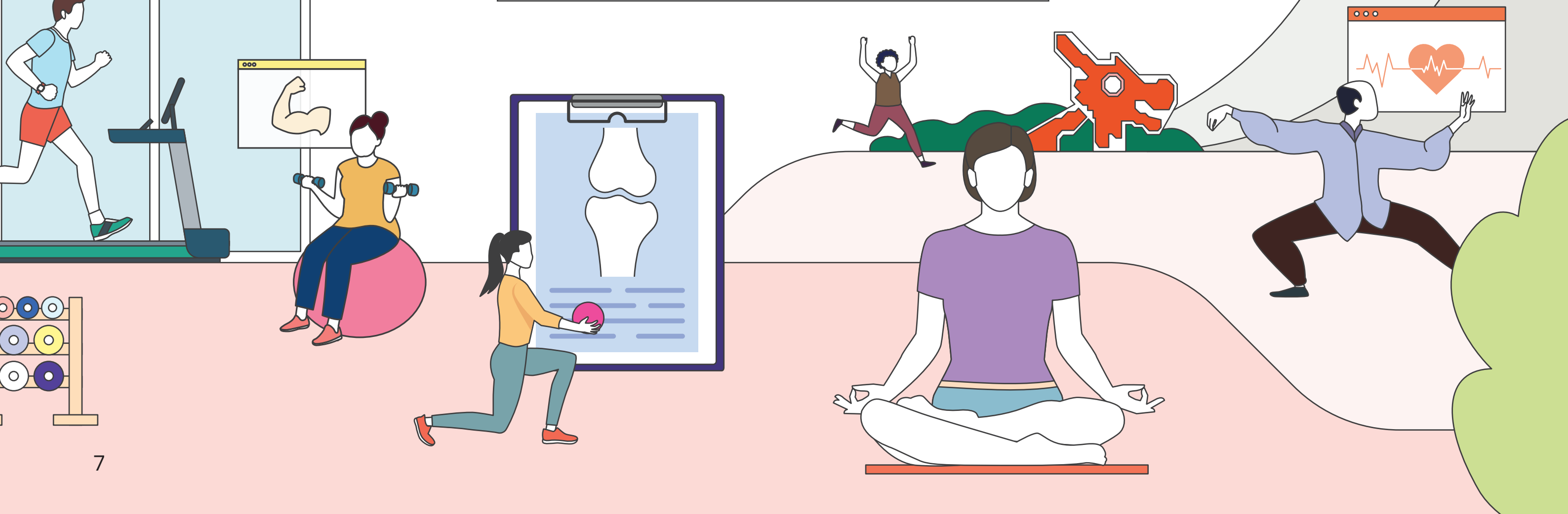

THE BENEFITS OF PHYSICAL ACTIVITY

EVERY MOVE COUNTS

No matter your ability, you will benefit from Moving More

THE BENEFITS OF PHYSICAL ACTIVITY

PHYSICAL WELLBEING

- ↑ Cardiometabolic health (e.g. Type-2 Diabetes)
- ↑ Weight management
- ↑ Bone and muscle health
- ↑ Quality of sleep
- ↑ Energy levels
- ↓ Various cancers
- ↓ Medical conditions and all-cause mortality

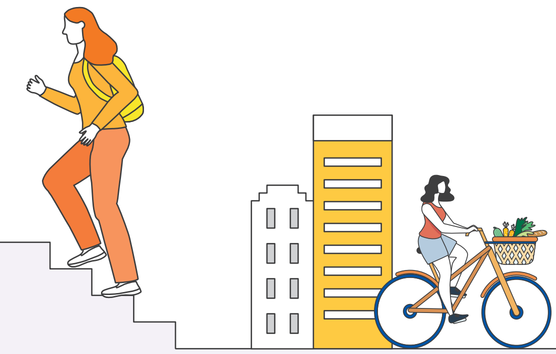

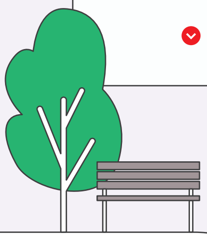

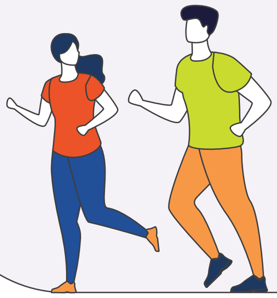

MENTAL WELLBEING

- ↑ Cognitive function
- ↑ Happiness and life satisfaction
- ↓ Depression, stress & anxiety
- ↓ Dementia

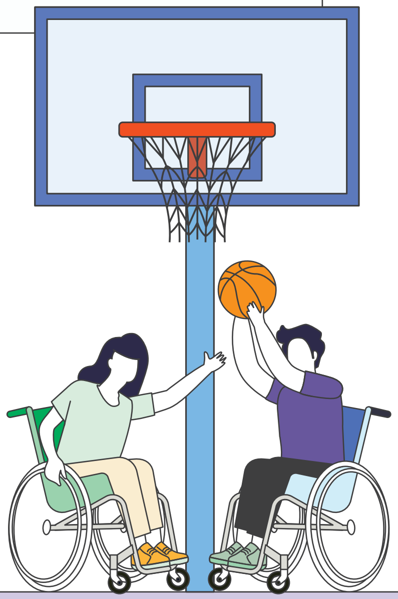

INDIVIDUAL DEVELOPMENT

- ↑ Creativity and critical thinking
- ↑ Social skills and interaction
- ↑ Academic performance and attention

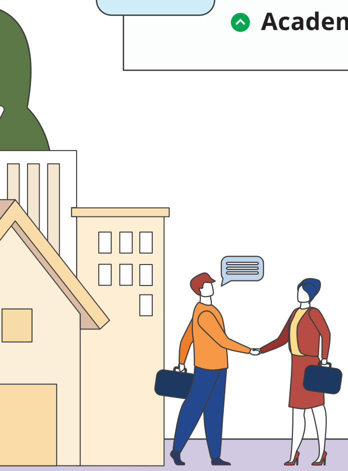

COMMUNITY DEVELOPMENT

- ↑ Social cohesion
- ↑ Sense of belonging
- ↑ National pride

IMPACT ON OUR PHYSICAL WELLBEING

An active lifestyle reduces the risk of Non-Communicable Diseases (NCDs)

Coronary Heart Disease

14%

Regular aerobic activity strengthens the heart muscle, reducing strain at rest.

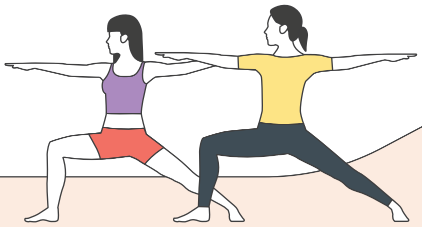

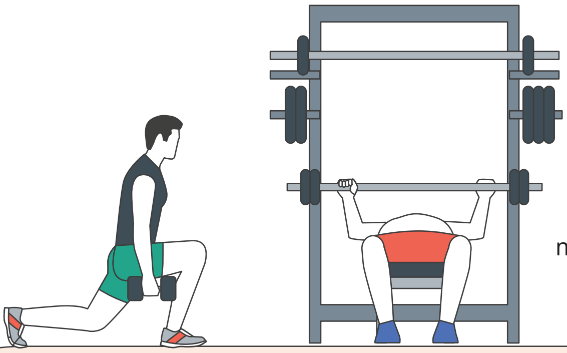

Osteoporosis

29%

Weight-bearing physical activity improves bone mineral density and reduces bone loss.

Uterine Cancer

20%

Colon Cancer

14%

Breast Cancer

25%

Cancer

Physical activity helps to maintain a healthy weight and regulates hormone levels, reducing risk of cancer in the following 8 site-specific cancers: bladder, breast, colon, endometrial, oesophageal adenocarcinoma, gastric, and renal.

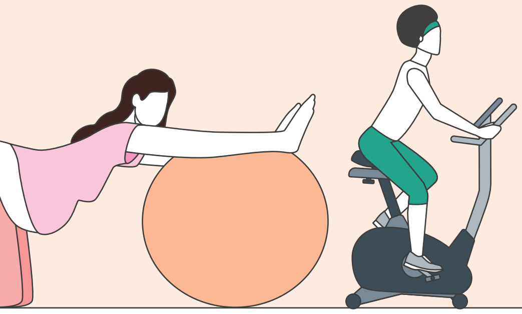

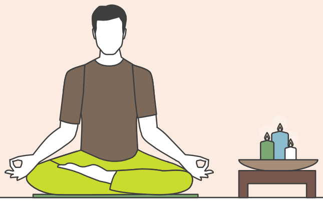

Type-2 Diabetes

26%

Physical activity increases insulin sensitivity and controls blood glucose levels.

Stroke

20%

Physical activity reduces risk of high blood pressure and cholesterol levels, which are key causes of strokes.

Hypertension

26%

Physical activity reduces cholesterol deposits in blood vessels, reducing blood pressure.

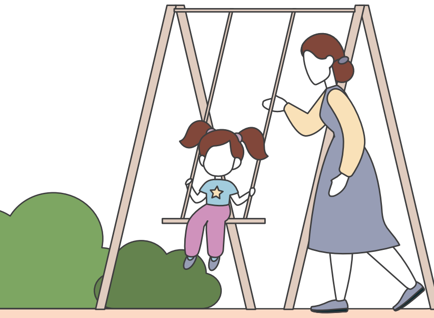

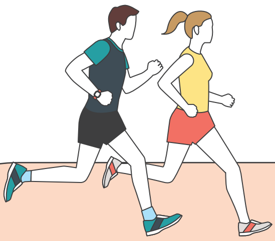

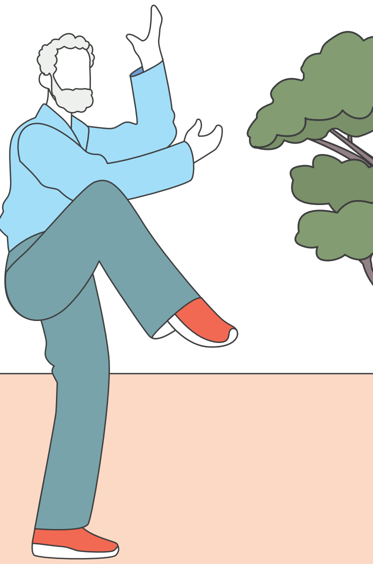

IMPACT ON OUR MENTAL WELLBEING

Physical activity can significantly improve brain health, increase functional capacity, and reduce the risk of mental illnesses

COGNITIVE FUNCTION

Mental agility and memory score (MMSE) ▲ **20%**

Physical activity improves blood flow to the brain and accelerates neurological adaptations, which optimises memory and executive functions in the brain.

EMOTIONAL HEALTH

Life satisfaction score ▲ **20%**

Physical activity increases various measures of self-esteem and confidence and regulates hormones which improves your mood.

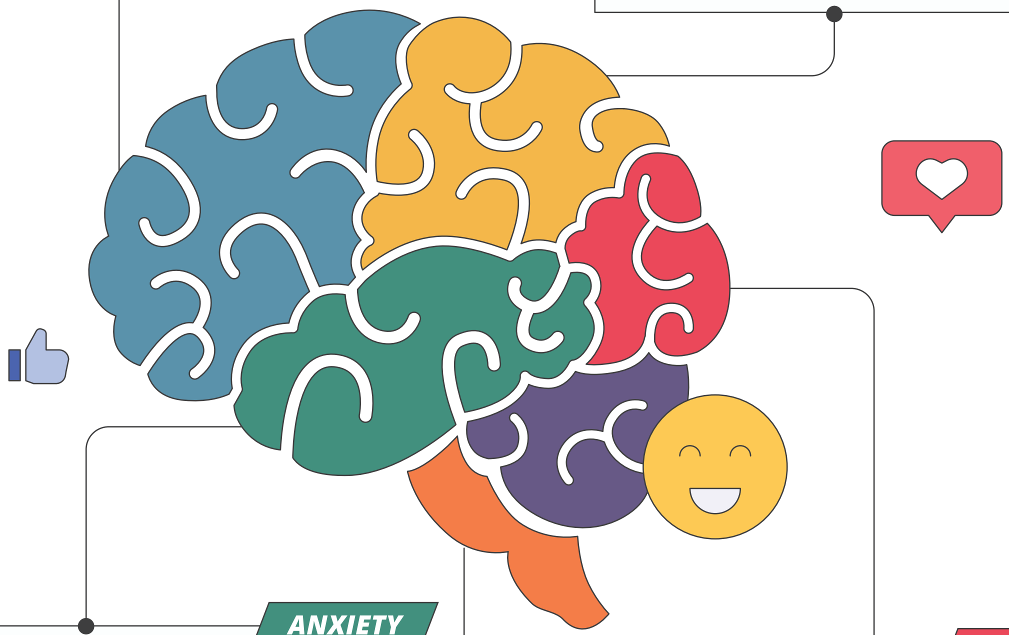

ANXIETY

Risk of developing anxiety ▼ **39%**

Physical activity reduces neurological reaction to stress (by regulating the reactivity of the HPA axis) reducing symptoms of anxiety.

DEPRESSION

Risk of developing depression ▼ **34%**

Physical activity optimises neurotrophin levels, which are vital to the maintenance of neurons controlling emotional function and mood.

ALZHEIMER'S DISEASE & DEMENTIA

Risk of developing Alzheimer's disease ▼ **45%**

Physical activity improves blood flow to the brain, preventing primary vascular dementia and reduces the risk of overall neurodegeneration.

IMPACT ON INDIVIDUAL AND COMMUNITY DEVELOPMENT

Engaging in physical activity, particularly in a group setting, improves an individual's psychosocial health, which leads to greater productivity and better quality of life.

Increases social interaction and improves social skills
Physical activity provides the opportunity for social interaction which helps improve interpersonal relationships.

Improves creativity and critical thinking
Physical activities, especially sports, require broader ways of thinking to succeed.

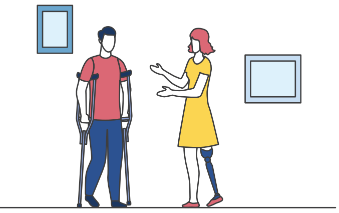

Moderately improves test scores
Physical activity results in better academic outcomes due to a greater ability to concentrate.

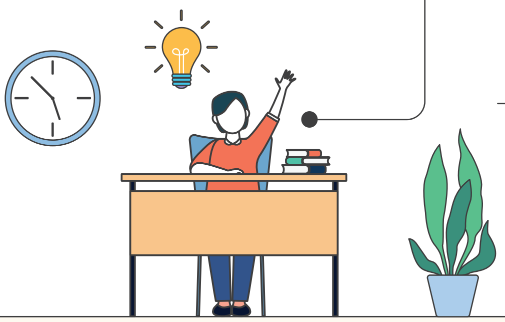

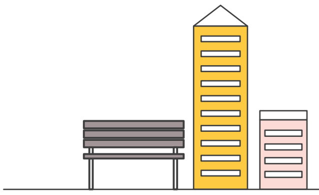

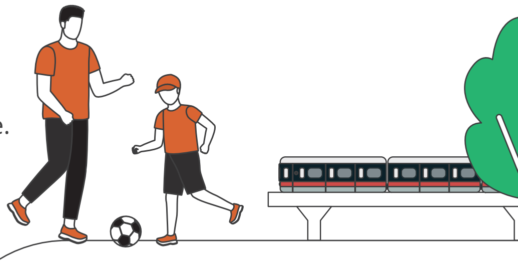

Acute bouts of exercise strengthens selective attention

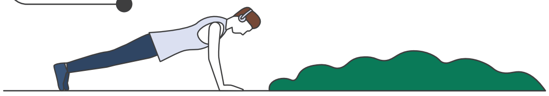

Additionally, the 'National Sports Participation Survey' shows that participation in sports or other group physical activities can provide benefits for community development.

Cohesion

- Participation in sports has the potential to bring people from diverse backgrounds together.
- Individuals are 25% more likely to interact with another race when they engage in sports.

Interaction

- Sports and group physical activities offer opportunities for social interaction, which can lessen social isolation.
- Individuals are 11% more likely to experience a sense of belonging when they engage in sports.

National Pride

- Physically active individuals are more likely to be engaged in their community, and have trust in state institutions.
- Individuals are 11% more likely to feel national pride when they engage in sports.

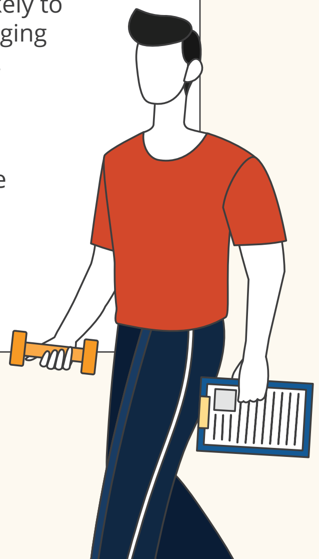

IMPACTS OF PHYSICAL ACTIVITY ON INDIVIDUALS THROUGHOUT DIFFERENT STAGES OF LIFE

*Not an exhaustive list

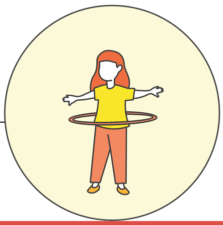

CHILDREN & ADOLESCENTS

PHYSICAL ACTIVITY IMPROVES:

- Bone health
- Mental health
- Cognitive function
- Motor skills
- Weight status
- Social skills

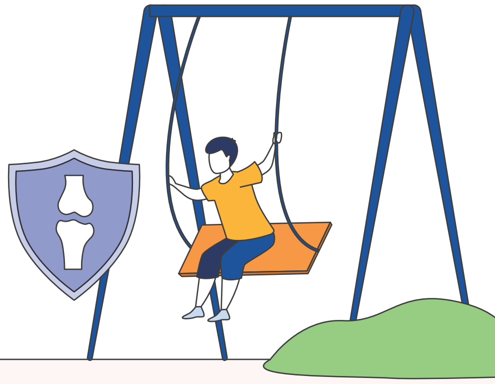

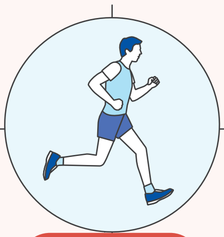

ADULTS

PHYSICAL ACTIVITY IMPROVES:

- Weight status
- Cognitive function
- Sleep
- Mental health

REDUCES RISK OF:

- Stroke and heart disease
- Hypertension
- Type-2 Diabetes
- 8 types of site-specific cancers

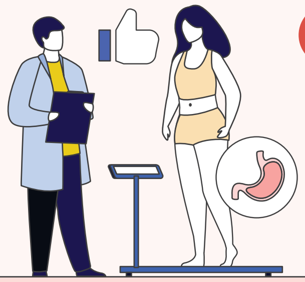

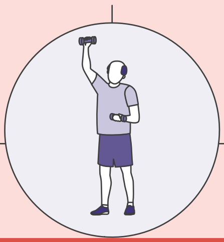

OLDER ADULTS

PHYSICAL ACTIVITY IMPROVES:

- Physical function
- Cognitive function
- Bone health

REDUCES RISK OF:

- Dementia
- Falls
- Frailty

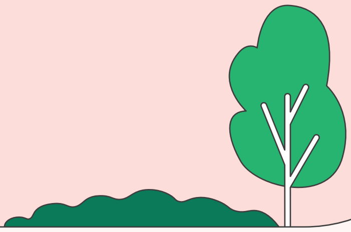

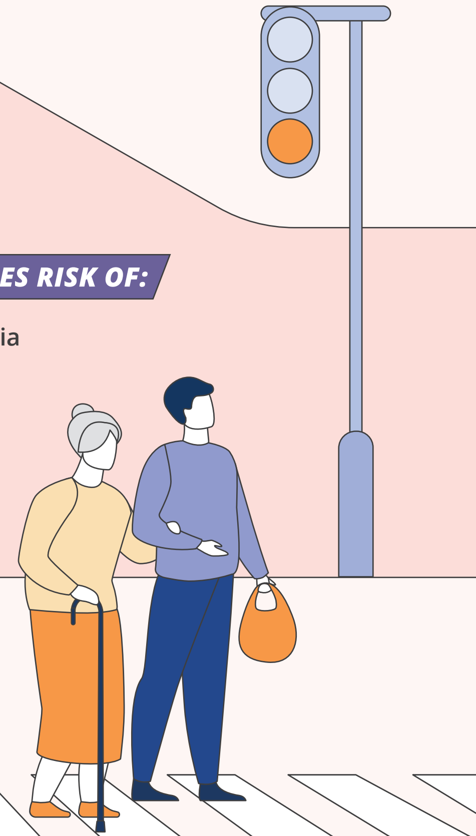

EXERCISE SAFETY

While physical activities bring about countless benefits, it is important to stay safe during physical activities. Use the SAFER acronym as a way of remembering the key safety tips:

- **S** is for screening, sustainability and suitability of exercise and equipment use
- **A** is for awareness of environment (weather), hydration, nutrition, and rest
- **F** is for fitness level, knowing one’s limits (ability and competency)
- **E** is for exercise with buddies to help take care of each other and be aware of emergency protocol and equipment
- **R** is for responsibility of taking ownership of one's safety and learn CPR/AED to support others if needed

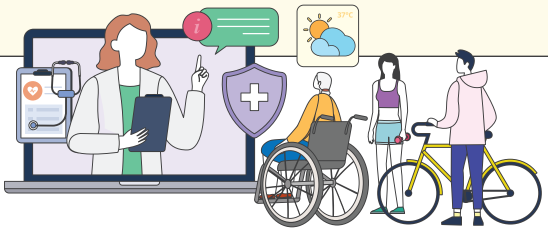

SUITABILITY

Based on your health/age/medical conditions, do you need to consult a health professional before starting?

- Use pre-participation screening tools such as the Get Active Questionnaire to assess your readiness to take part in physical activity.
- If you have been diagnosed with any illness or are unsure what activities you can do, consult a medical/ health professional before you begin.

Are you wearing suitable clothing? Do you have appropriate equipment? Is your equipment safe and in working condition?

- Before you start any type of physical activity, ensure that you have the appropriate attire, and equipment in working condition.

AWARENESS

What is the weather like? What time is best to exercise? Are you hydrated / Have ready access to water?

- Avoid adverse weather or heat conditions, e.g. rain or noon hours when the Sun is the hottest. For heat management, consider first ensuring you are heat acclimatised, pace accordingly, take more frequent breaks, and seek out shelters when it is hot/rainy day. To also dress light and hydrate appropriately.

Is the environment safe?

- Ensure that the space around is free of trip-hazards.

FITNESS

When did you last exercise?

- If you have not exercised in a while, start slow and build up intensity and duration gradually. Listen to your body and adapt exercises accordingly.

How are you feeling? Are you appropriately warmed up?

- Listen to your body and adapt exercises accordingly. It is important to incorporate warm-ups and cool-downs of at least 5-10 minutes into your routine.

EXERCISE WITH A BUDDY

Do you have a buddy to exercise with?

- Exercising with a friend will allow you to support one another if anything unforeseen happens.

Are you aware of the emergency protocol and safety equipment close to your exercise location?

- Knowing the emergency protocol and where the closest Automated External Defibrillator (AED) is when exercising may help save a life.

RESPONSIBILITY

Do you know CPR/AED?

- Take ownership and learn CPR/AED so that you can support those around you in case of an unexpected event.

PHYSICAL HEALTH STATUS & ACTIVITY LEVELS IN SINGAPORE

PERCENTAGE OF SINGAPOREANS AGED 5 TO 19 YEARS OLD WHO ARE OVERWEIGHT (2016)

27% MALE

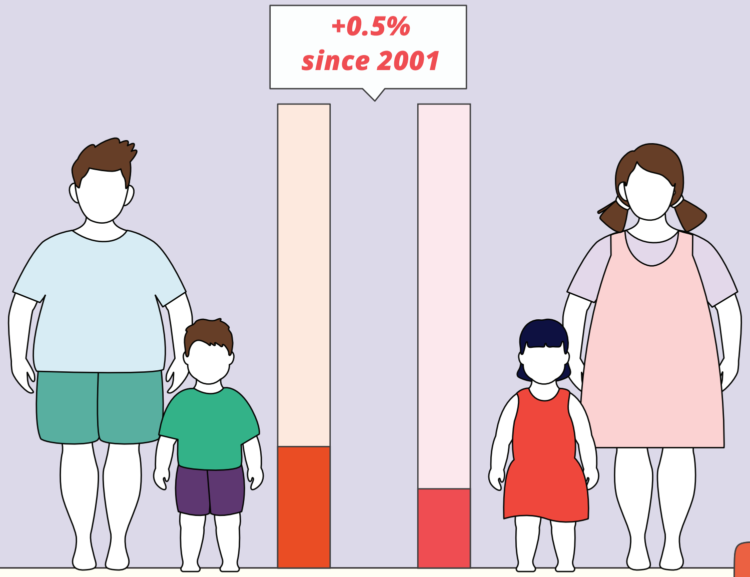

18% FEMALE

PERCENTAGE OF SINGAPOREANS AGED 11 TO 17 YEARS OLD THAT MEET THE WHO PHYSICAL ACTIVITY GUIDELINES (2016)

THE WHO RECOMMENDS:

An average of at least 60 minutes per day of moderate- to vigorous-intensity activity.

Incorporating vigorous-intensity aerobic activities, including activities that strengthen muscle and bone, minimally 3 times a week.

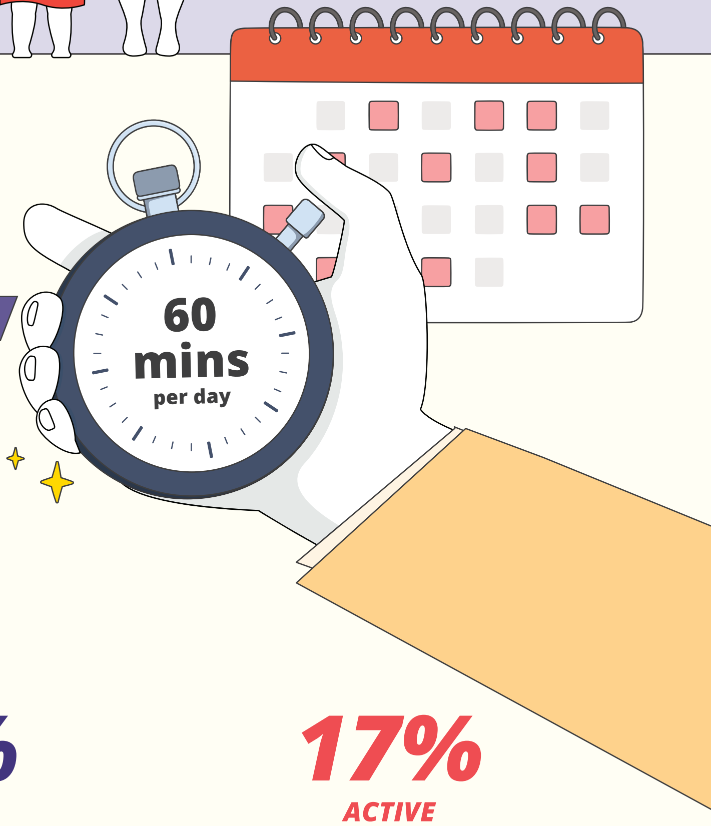

30% ACTIVE BOYS
+8% since 2001

24% ACTIVE OVERALL
+5% since 2001

17% ACTIVE GIRLS
+2% since 2001

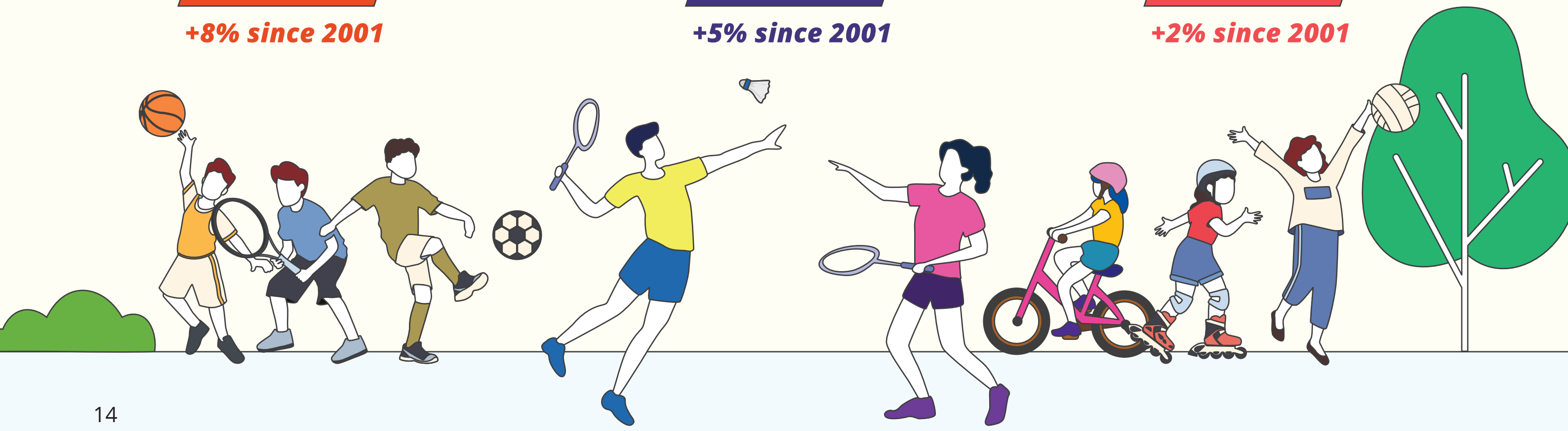

PHYSICAL HEALTH STATUS & ACTIVITY LEVELS IN SINGAPORE

PERCENTAGE OF NON-COMMUNICABLE DISEASES HAVE INCREASED IN RECENT YEARS FOR SINGAPOREANS AGED 18 TO 74 YEARS OLD (2020)

TYPE-2 DIABETES

+0.7%

since 2017

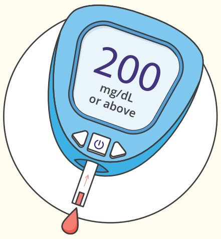

OBESITY

+1.9%

since 2017

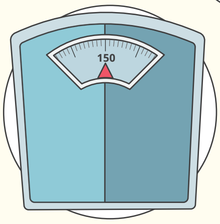

PERCENTAGE OF SINGAPOREANS AGED 18 TO 74 YEARS OLD THAT MEET THE WHO PHYSICAL ACTIVITY GUIDELINES (2020)

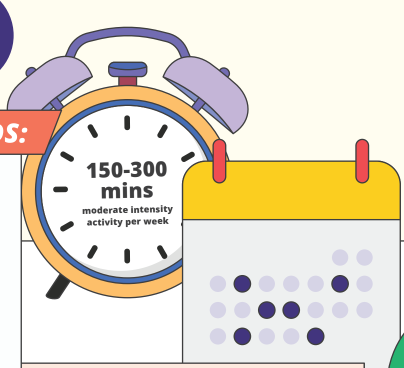

THE WHO RECOMMENDS:

A minimum of 150–300 minutes of moderate-intensity aerobic physical activity per week

Engage in muscle-strengthening activities at moderate or greater intensity on at least 2 days a week

76.4%

ACTIVE

BOTH GENDERS EQUAL

-3.7% since 2019

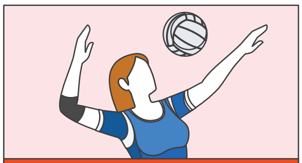

Leisure-time physical activity

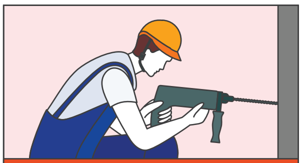

Activity at work

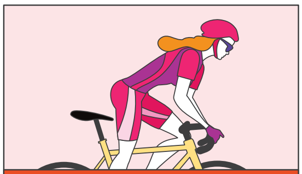

Active travel

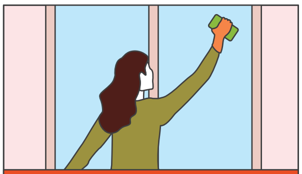

Household chores

33.8%

engaged in sufficient muscle strengthening activity in 2020

- More common among young adults aged 18 to 29 years (44.1%) compared with 30 to 59 years (~33.3%) and 60 to 74 years (25.5%)
- Males at 40.1% while females 27.8%

Includes those meeting both 'high' and 'moderate' classification of total physical activity
High: Vigorous Intensity activity on at least 3 days of 1,500 MET** Minutes*/week OR 7 or more days of activities of a minimum of 3,000 MET Mins
Moderate: 3 or more days of vigorous-intensity activity of at least 20 mins /day, OR 5 or more days of moderate-intensity activity of at least 30 mins/day OR 5 or more days of activities achieving a minimum of at least 600 MET Minutes/week

PHYSICAL HEALTH STATUS & ACTIVITY LEVELS IN SINGAPORE

Since the release of the guidelines in 2011, an overall increase in physical activity levels was observed across all adult age groups, despite small dip in 2020 which is likely attributed to the COVID-19 pandemic

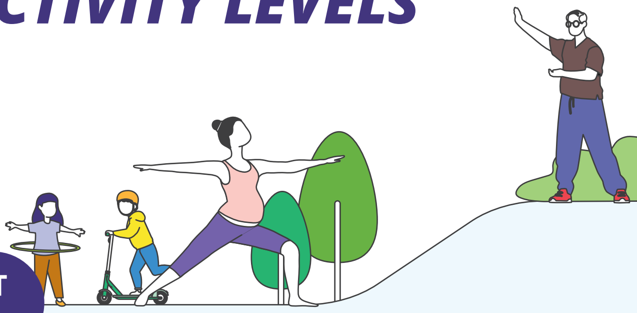

PERCENTAGE OF ADULTS AGED 18 TO 74 YEARS OLD THAT MEET THE WHO PHYSICAL ACTIVITY GUIDELINES

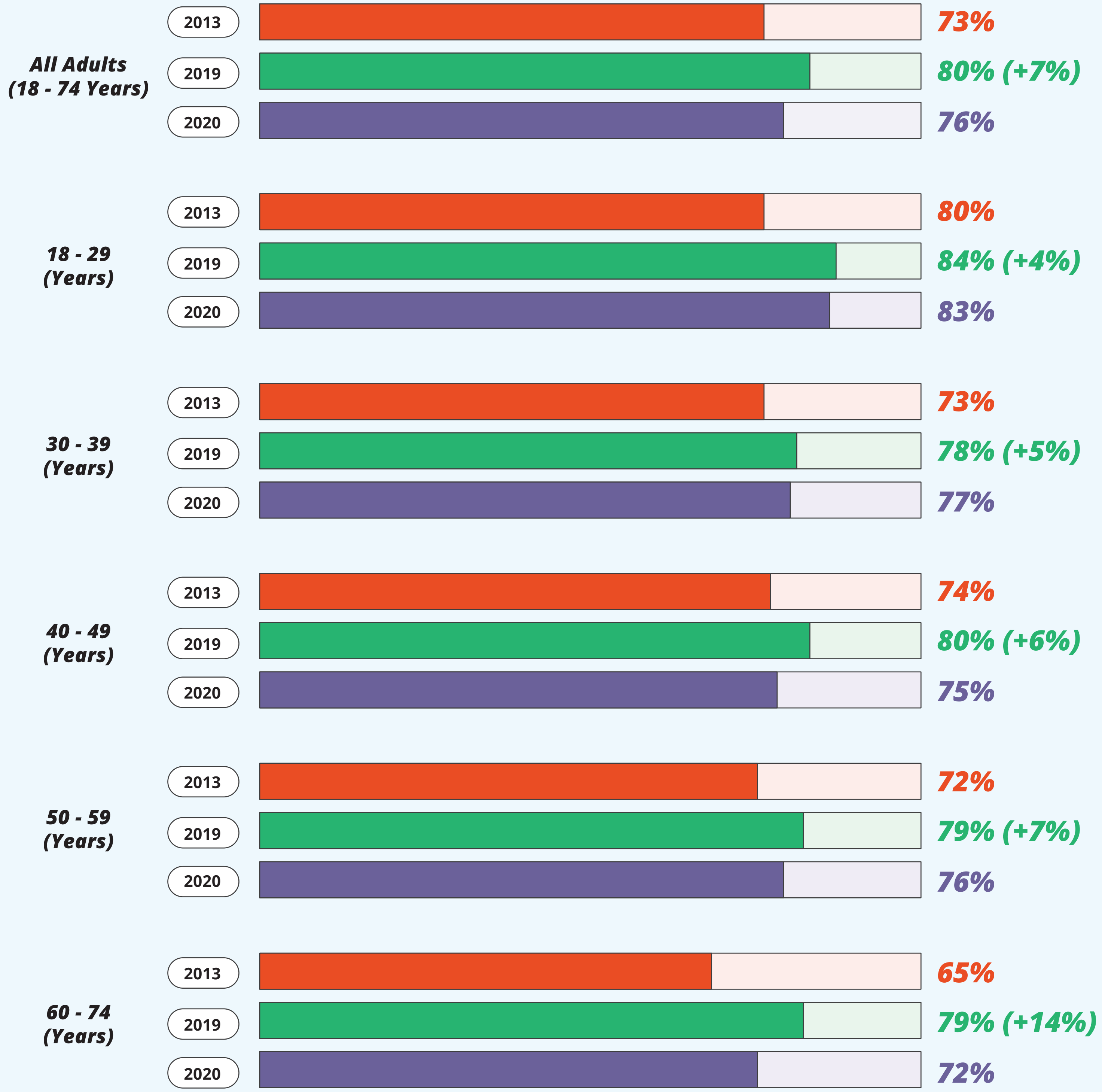

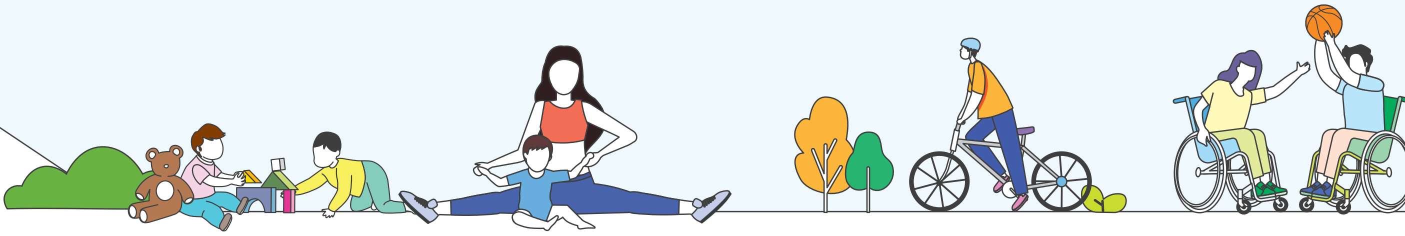

GUIDELINES BY SUB-POPULATION: PRESCHOOL, SCHOOL CHILDREN & YOUTHS

UPDATES IN THE SINGAPORE PHYSICAL ACTIVITY GUIDELINES (SPAG) PRESCHOOL (0 - 6 YEARS)

Previous (2013)	What's New
Limit sedentary entertainment screen time to 2 hours.	Sedentary time limit revised to a maximum of 1 hour.
Engage in physical activity for 180 minutes daily regardless of age.<br><br>Sleep recommendations not included.	Activity recommendations broken into 3 further sub-groups to reflect different developmental needs as preschool children grow.<br><br>Introduction of sleep time recommendations.

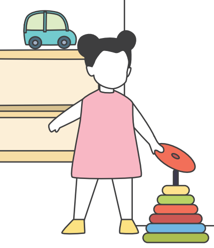

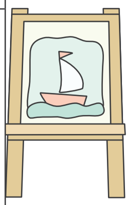

UPDATES IN THE SINGAPORE PHYSICAL ACTIVITY GUIDELINES (SPAG) SCHOOL CHILDREN ADN YOUTHS (7 - 17 YEARS)

Previous (2013)	What's New
Limit sedentary entertainment screen time to 2 hours.<br><br>Break up sedentary periods of 90+ minutes with 5-10 minutes of physical activity.	Removed time-specific recommendations.
Engage in physical activity for 60 minutes daily.<br><br>Minimum bouts of 5 minutes.<br><br>Fundamental movement skills not emphasised in previous edition.	Engage in an average of 60 minutes of physical activity per day over the week.<br><br>No minimum duration.<br><br>Greater emphasis on the importance of developing fundamental movement skills in school children and youths by engaging in different activities.

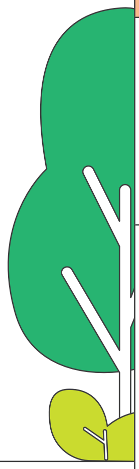

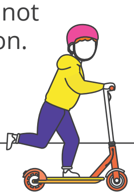

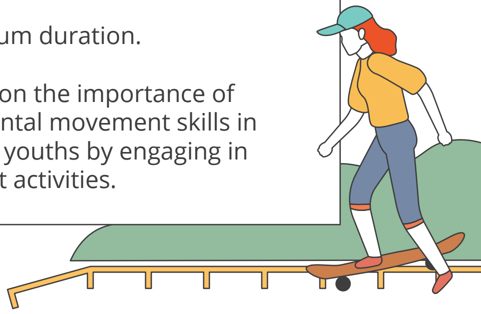

IT IS RECOMMENDED THAT...

Preschool, school children and youths should limit the amount of time spent in sedentary behaviours, especially recreational screen time as it has been highlighted as the key factor. Instead, they should engage in a variety of physical activities regularly.

Infants below one years old should be encouraged to engage in at least 30 minutes of tummy time per day. Young school children should aim for at least 180 minutes of physical activity of any intensity spread throughout each day. It doesn't have to be all at once and every minute counts. Additionally, for all children aged three years and older, 60 minutes of the time spent actively should be of moderate- to vigorous-intensity.

Preschool, school children and youths should also engage in vigorous-intensity aerobic activities, muscle and bone strengthening exercises at least 3 days a week, where doing a variety of activities can help to build their Fundamental Movement Skills (FMS).

SINGAPORE PHYSICAL ACTIVITY GUIDELINES FOR PRESCHOOL CHILDREN (0 - 6 YEARS)

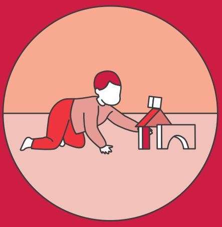

Preschool children should meet all recommendations for physical activity, sedentary behaviour and sleep habits to reap health benefits associated with regular physical activity and reduced sedentary behaviour.

MOVE WITH FUN & GROW

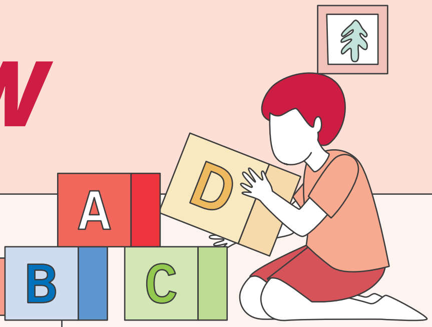

RECOMMENDATIONS

0-2 YEARS:

SEDENTARY TIME	PHYSICAL ACTIVITY	QUALITY SLEEP
**START PLAYING**<br>Limit the amount of time spent being sedentary, with recreational screen time not recommended.<br><br>Instead, engage in imaginative play and storytelling activities.	**PLAY MORE**<br><u>0-1 years</u><br>Encourage interactive floor-based activities for a minimum of 30-minutes a day.<br><br><u>1-2 years</u><br>Spend at least 180 minutes doing a variety of physical activities of any intensity, spread throughout the day.<br><br>Aim for daily outdoor play.	**SLEEP WELL**<br>0-3 months: 14 to 17 hours<br><br>4-11 months: 12 to 15 hours<br><br>1-2 years: 11 to 14 hours

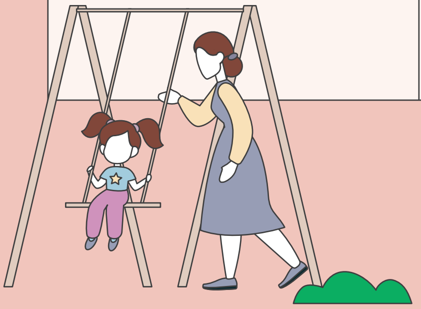

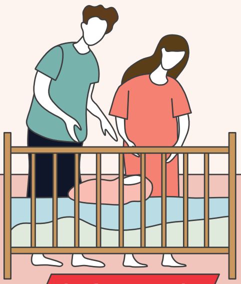

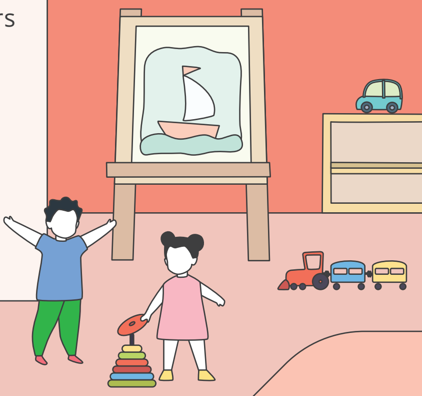

3-6 YEARS:

SEDENTARY TIME	PHYSICAL ACTIVITY	QUALITY SLEEP
**START PLAYING**<br>Limit the amount of time spent being sedentary, keeping recreational screen time to less than an hour a day.	**PLAY MORE**<br>Spend at least 180 minutes doing a variety of physical activities, of which at least 60 minutes should be moderate- to vigorous-intensity activity, spread throughout the day.	**SLEEP WELL**<br>3-4 years: 10 to 13 hours<br><br>5-6 years: 9 to 13 hours

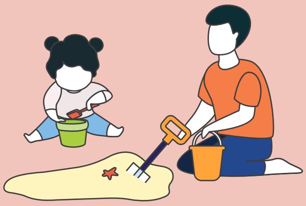

TIPS

- Create a home environment that encourages movement and exploration.
- Introduce activity-based games. E.g. Animal movements, dance activity.
- Encourage a wide variety of movements like running, jumping, catching, throwing, and kicking, in different environments, to build Fundamental Movement Skills (FMS).
- Visit outdoor playgrounds for your child to engage in social play and interact with others while being active.
- Establish a consistent bedtime routine to help your child develop good habits.

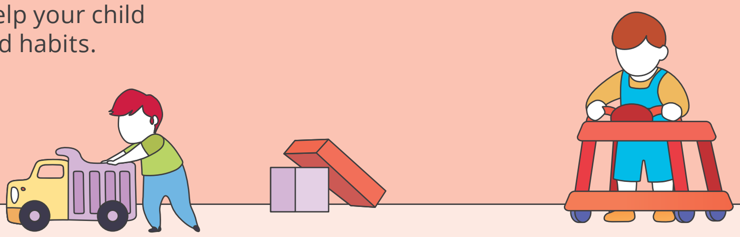

EXERCISE INTENSITY

- **Light** – Can talk in full sentences and sing
- **Moderate** – Can talk in phrases or short sentences but cannot sing
- **Vigorous** – Have difficulty talking

SINGAPORE PHYSICAL ACTIVITY GUIDELINES FOR SCHOOL CHILDREN & YOUTHS (7 - 17 YEARS)

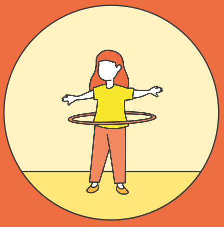

School children and youths can engage in physical activity through many different settings and not only through recreation and leisure, such as home, in school, and in community settings. They should meet all recommendations for physical activity, sedentary behaviour and sleep habits to reap health benefits associated with regular physical activity and reduced sedentary behaviour.

MOVE WITH PLAY & GROW

RECOMMENDATIONS

PLAY FOR 60 & MIX IT UP / *Physical Activity*

Accumulate an average of ***60 minutes in moderate- to vigorous-intensity aerobic activity per day across the week***.

*Every minute of vigorous-intensity activity can generally be considered as two minutes' worth of moderate-intensity activity.

A variety of ***vigorous-intensity aerobic activities, muscle- and bone-strengthening exercises*** should be incorporated ***at least 3 days a week*** to promote the development of movement skills.

PLAY MORE / *Sedentary Behaviour*

Limit the amount of time spent being sedentary, particularly recreational screen time, by ***engaging in activities of any intensity, including those of light-intensity***.

SLEEP WELL / *Quality Sleep*

7-13 years: ***9 to 12 hours***
14-17 years: ***8 to 10 hours***

TIPS

- Incorporate active travel into the weekly routine, such as walking or cycling to school.
- Explore a variety of activities to help school children and youths develop active hobbies or interests.
- Visit outdoor playgrounds for your child to engage in social play and interact with others while being active.
- Stand up while studying or take movement breaks in between study sessions.
- Consider joining a sports team/club/organisation to experience physical activity in a social setting, which helps school children and youths stay motivated.

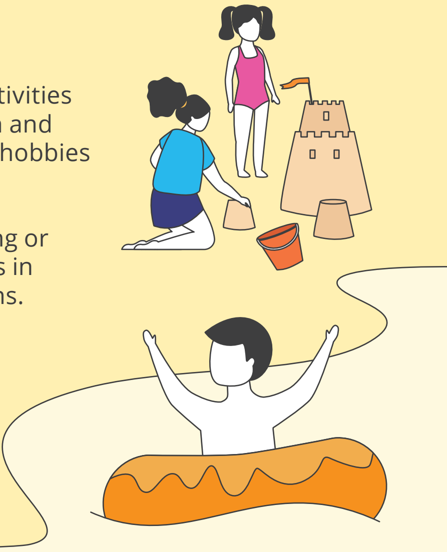

EXERCISE INTENSITY

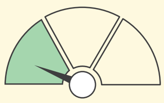

Light
Can talk in full sentences and sing

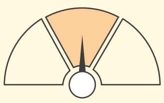

Moderate
Can talk in phrases or short sentences but cannot sing

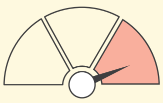

Vigorous
Have difficulty talking

EXAMPLES OF AEROBIC, MUSCLE & BONE STRENGTHENING ACTIVITIES FOR SCHOOL CHILDREN

*Not an exhaustive list

	MODERATE-INTENSITY	VIGOROUS-INTENSITY
**AEROBIC** 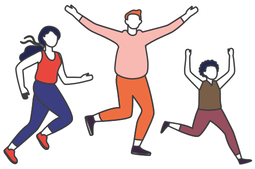	• Games at the Playground (Hide and Seek)<br>• Commute by cycling<br>• Casual racquet sports	• Games at the Playground (Tag/Catching)<br>• Mountain biking<br>• Competitive racquet sports
**MUSCLE STRENGTHENING*** 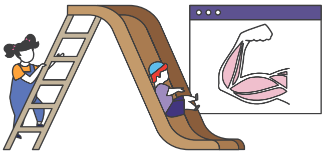	• Games at the playground (e.g. tug-of-war)<br>• Climbing	• Circuit training<br>• Hopping, jumping, and rope-skipping<br>• Playing a football match
**BONE STRENGTHENING*** 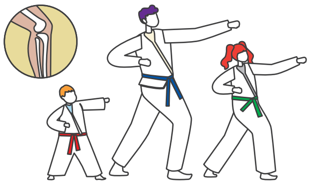	• Games at the playground (e.g. hopscotch)<br>• Casual basketball or netball games<br>• Gymnastics	• Martial arts<br>• Competitive basketball or netball games<br>• Dancing

* All muscle and bone strengthening activities of moderate- or vigorous-intensity are also considered as aerobic activities

EXAMPLES OF FUNDAMENTAL MOVEMENT SKILLS

LOCOMOTOR SKILLS

Enabling children to move through different environments, moving their bodies from one location to another.

Examples of key locomotor skills:

- **Walking**
- **Running**
- **Jumping**
- **Hopping**
- **Skipping**
- **Sliding**

OBJECT CONTROL SKILLS

Making controlled contact with objects using parts of the body or using an implement.

Examples of object control skills:

- **Rolling**
- **Throwing**
- **Catching**
- **Kicking**
- **Striking**
- **Dribbling**

STABILITY SKILLS

Maintaining and acquiring balance in static and dynamic movements.

Examples of stability skills:

- **Balancing**
- **Turning**
- **Twisting**
- **Stretching**
- **Transferring of weight**

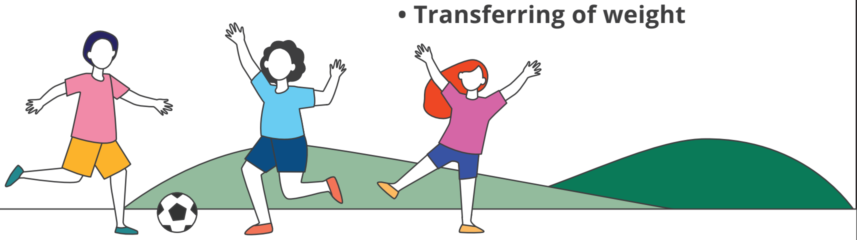

EXERCISE INTENSITY

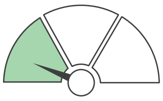

Light
Can talk in full sentences and sing

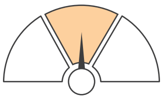

Moderate
Can talk in phrases or short sentences but cannot sing

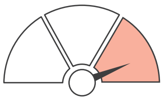

Vigorous
Have difficulty talking

CASE STUDIES: PRESCHOOL CHILDREN

CASE STUDY #1

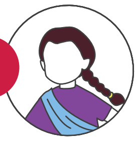

SHANTI, 3

Preschooler with two busy working parents

Shanti is a quiet girl who enjoys being at home with her parents. She sleeps for 12 hours each night and has been in preschool since she was six months old. Both her parents are office workers, and her dad usually picks her up from school at 7pm. Due to the busy schedules of her parents, Shanti relies on her school teachers to support her daily physical activity needs. This usually includes some dancing, games, and arts and craft tactile work.

On weekends, Shanti goes to the playground in the evening with her parents to meet her friends and she engages in a combination of running, jumping, and climbing for 60 minutes whilst socialising. Her busy parents who work on weekends tend to give her a handphone to keep her occupied. Recently, this has become more frequent and resulted in less active time for Shanti.

Shanti's parents are doing great by encouraging her to play at the playground on weekends for an average of 60 minutes per day as this gives her an opportunity to activate a variety of different muscle groups and energy systems in an unstructured play setting. However, on weekdays, it is tough for Shanti to meet her daily 60 minutes of physical activity due to the late pick-ups from school. For working parents with young children, it is important to:

- Plan for some form of physical activity for young kids such as play time at the playground or at home.
- Expose your kids to ball activities to develop their movement skills.

CASE STUDY #2

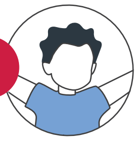

JIE YANG, 5

Energetic and active preschooler

At preschool, Jie Yang gets light to moderate physical activity time through class activities such as dancing and organised outdoor ball games. At home, Jie Yang is always on the move, particularly after dinner when his playtime includes moderate-intensity activities such as chasing, hide-and-seek, and pillow fights. Sometimes, evening playtime extends beyond his bedtime, and he goes to bed as late as 11.00pm. The early start to the day for school results in difficult mornings as Jie Yang struggles to wake up.

On weekends, Jie Yang goes swimming with his dad in the morning and his mother arranges for playground play dates with his preschool friends in the afternoon. This allows him to run, jump, and fall in a safe environment which supports the development of his movement skills in an unstructured way. The exposure to water and land-based activities also helps develop his movement competency in a range of environments.

Jie Yang is doing well by mixing up his activities across the week with unstructured and structured play. Parents should:

- Encourage your child to sleep early, particularly on weekdays, to support recovery and rest.
- Combine both structured and unstructured play in your child's routine to allow him/her to build physical competency in diverse environments like land and water.

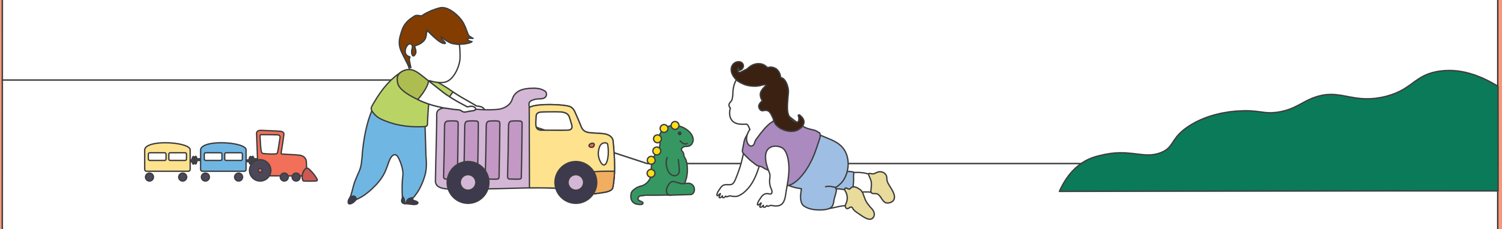

CASE STUDIES: SCHOOL CHILDREN & YOUTHS

CASE STUDY #1

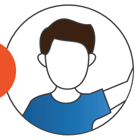

DE WEI, 9

Sedentary school child who enjoys e-games

De Wei is a primary school boy who enjoys e-gaming. Apart from walking to and from school on weekdays, he does not engage in any other structured physical activity. De Wei's biggest passion is playing online games with his friends on weekends. When he is not gaming, he is studying for his school exams. His parents spend a lot of time supporting De Wei in his studies and feel that there is no time for leisure time physical activity. De Wei often feels lethargic and tired, and he tends to eat high-sugar foods to give him energy boosts before school or during an intense gaming session.

While De Wei is getting some light intensity exercises intermittently throughout the week, he should be aiming for higher levels of physical activity. If your child is also passionate about e-gaming, consider the following:

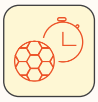

Always set aside time throughout the week for leisure time physical activity as it helps to boost your child's mood and improves critical thinking.

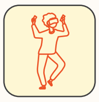

Find a gaming system that involves movement to increase physical activity while gaming. Activities like dancing, tennis, bowling and boxing have been adapted to a virtual format so you can now interact with the game as a form of physical activity.

CASE STUDY #2

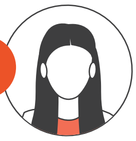

EMMA, 5

Teenage girl with a packed academic and social calendar

Emma is a secondary school girl who enjoys hip hop dance. This aerobic physical activity is intense, and Emma takes part in dance classes three times a week, with each session lasting 90 minutes. This adds up to a total of 4.5 hours of physical activity each week. Besides dancing, Emma takes part in her weekly physical education classes in school.

As the pressure from school continues to build up, Emma finds herself becoming more inactive in and out of school when she's not dancing with her friends. She feels tired all the time and struggles to squeeze all her studies and schoolwork into the time she has after school. On weekends when she's not studying, Emma likes to go for a walk at MacRitchie reservoir with her mother and has been doing it since she was a little girl. She finds the light-intensity walk in a nature setting calming and enjoys conversations with her mother about life in general. Like many of her friends, Emma spends many hours in front of a screen doing schoolwork and catching up on the latest social media trends.

Emma can focus on meeting the sleep recommendations of 8 to 10 hours every night to help her stay energised. This will also have a positive influence over her attention, memory, learning, behaviour, emotional regulation, quality of life, mental and physical health. She is doing great on a weekly basis and engages in physical activities. She has found a hobby that she is passionate about, and this will help to sustain the level of physical activity on a long-term basis. As an activity, dancing can engage multiple muscle groups and energy systems in each bout of activity. The balance and coordination component of dancing may also help to improve Emma's quality of movement and sense of movement competency. For most youths, it is important to:

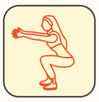

Include activities that require multi-directional bodyweight movement to improve strength.

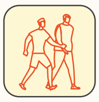

Head out during the weekends with friends and family for a stroll, and enjoy nature for light-intensity physical activity.

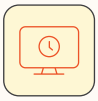

Limit screen time as it may impact one's quality of sleep.

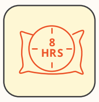

Have a consistent sleep routine and sleep for about 8 to 10 hours every night.

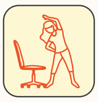

Build in regular breaks to move around during times of prolonged sitting or inactivity.

SUMMARY OF EVIDENCE: PRESCHOOL CHILDREN

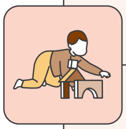

CHILDHOOD OBESITY:

Regular physical activity has been linked to an increase of healthy weight children in this age group, with carry-over effects that can persist into adulthood.

HABITS OF PHYSICAL ACTIVITY:

Physical activity habits established during childhood are likely to be carried through into adolescence.

COGNITIVE DEVELOPMENT:

Lower levels and quality of sleep are associated with reduced cognitive function and poorer emotional regulation amongst young children.

MUSCULOSKELETAL DEVELOPMENT:

Moderate-intensity physical activity has been proven to improve bone mineral density and muscular development, and promotes the development of motor skills.

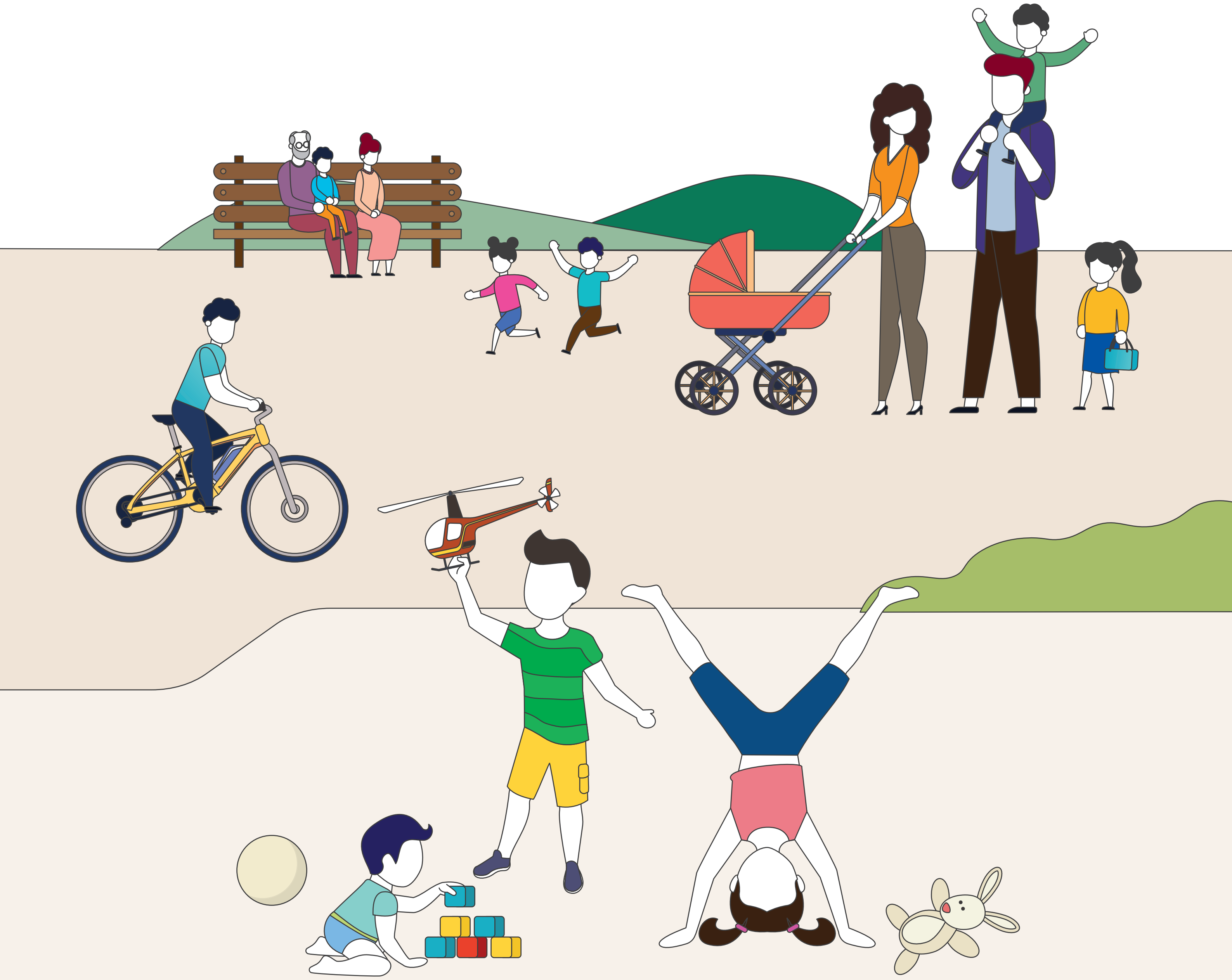

SUMMARY OF EVIDENCE: SCHOOL CHILDREN & YOUTHS

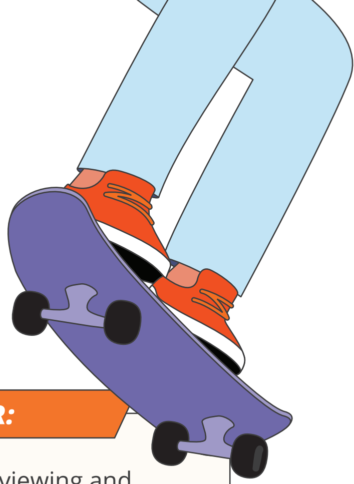

SEDENTARY BEHAVIOUR

GENERAL FITNESS:

Sedentary behaviour, especially recreational screen time, is related to poorer health outcomes, e.g. poorer fitness and cardiometabolic health in children and adolescents.

SOCIAL BEHAVIOUR:

Increased duration of television viewing and video gaming are significantly associated with unfavourable measures of behavioural conduct/pro-social behaviour.

OBESITY:

More than 2 hours of sedentary behaviour per day was positively associated with increased childhood obesity, as compared to lower durations of sedentary behaviour.

MENTAL HEALTH:

The use of screen during leisure time is associated with depressive symptoms and psychological distress among children and adolescents.

AEROBIC ACTIVITY

FITNESS:

Increased physical activity improves cardiorespiratory fitness and musculoskeletal fitness in children and adolescents.

MENTAL HEALTH:

Exercising and endurance training have a beneficial effect on depressive symptoms in children.

COGNITIVE FUNCTION & ACADEMIC OUTCOMES:

Physical activity has positive effects on cognitive function and academic outcomes (e.g. school performance, memory, and executive function).

CARDIOMETABOLIC HEALTH:

Regular physical activity in children and adolescents is positively associated with beneficial cardiometabolic health outcomes.

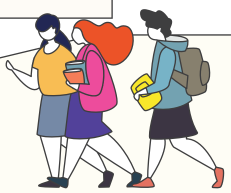

VIGOROUS-INTENSITY AND MUSCLE/BONE STRENGTHENING ACTIVITIES

GROWTH AND DEVELOPMENT:

Vigorous-intensity activity promotes strength gains, strong joints, and healthy bones, which are vital for optimal growth and development.

BONE HEALTH:

Physical activity increases bone mass, bone mineral content, and bone strength, which can help to protect from osteoporosis and related fractures later in life.

MOVEMENT SKILLS:

Physical activity in school children and youths is positively associated with increased proficiency in motor/movement skills (physical literacy).

CARDIORESPIRATORY FITNESS:

High-intensity interval training, compared with moderate-intensity continuous training, had a moderate beneficial effect on cardiorespiratory fitness.

GUIDELINES BY SUB-POPULATION: ADULTS

UPDATES IN THE SINGAPORE PHYSICAL ACTIVITY GUIDELINES (SPAG): ADULTS (18 - 64 YEARS)

Previous (2011)	What's New
Break up sedentary periods of 90+ minutes with 5-10 minutes of physical activity.	Removed time-specific recommendations.
Minimum bouts of 10 minutes of moderate-intensity physical activity per week.	No minimum duration.
Single time targets (e.g. 150 minutes of moderate-intensity aerobic activity per week).	Clocking in a target range e.g. 150 to 300 minutes of moderate-intensity activity per week.
No minimum intensity for muscle strengthening activity.	Muscle strengthening activity at moderate- or vigorous-intensity is encouraged, at least 2 days a week.

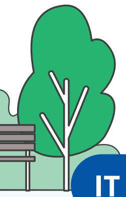

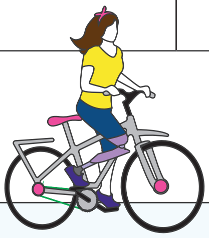

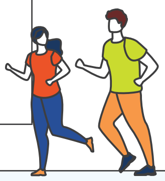

IT IS RECOMMENDED THAT...

Any level of physical activity is better than none. Where possible, adults should break up prolonged sedentary periods with light physical activity for health benefits.

Adults should accumulate a total equivalent of 150 to 300 minutes of moderate-intensity activity (mostly aerobic) throughout the week for health benefits. Spreading the activity throughout the week minimises the risk of injury and prevents excessive fatigue which can contribute towards meeting the recommendations.

Additionally, adults should engage in moderate-intensity muscle strengthening activities targeting the major muscle groups at least twice per week to build their strength. Finding the suitable weight/resistance is key and a general good practice is to complete 8 to 12 repetitions per set to achieve strengthening outcomes.

For adults with chronic conditions, the same recommendations and tips are still applicable but if unsure on how to begin, consult a health professional to determine a suitable exercise regime.

Meeting the recommendations can offer additional health benefits, such as:

Diabetes – Resistance training can aid in controlling hormone levels
Obesity – Physical activity expands energy and by engaging in sufficient activity to burn off excess calories, it can contribute to weight loss
Hypertension – Physical activity strengthens your heart and promotes better blood flow

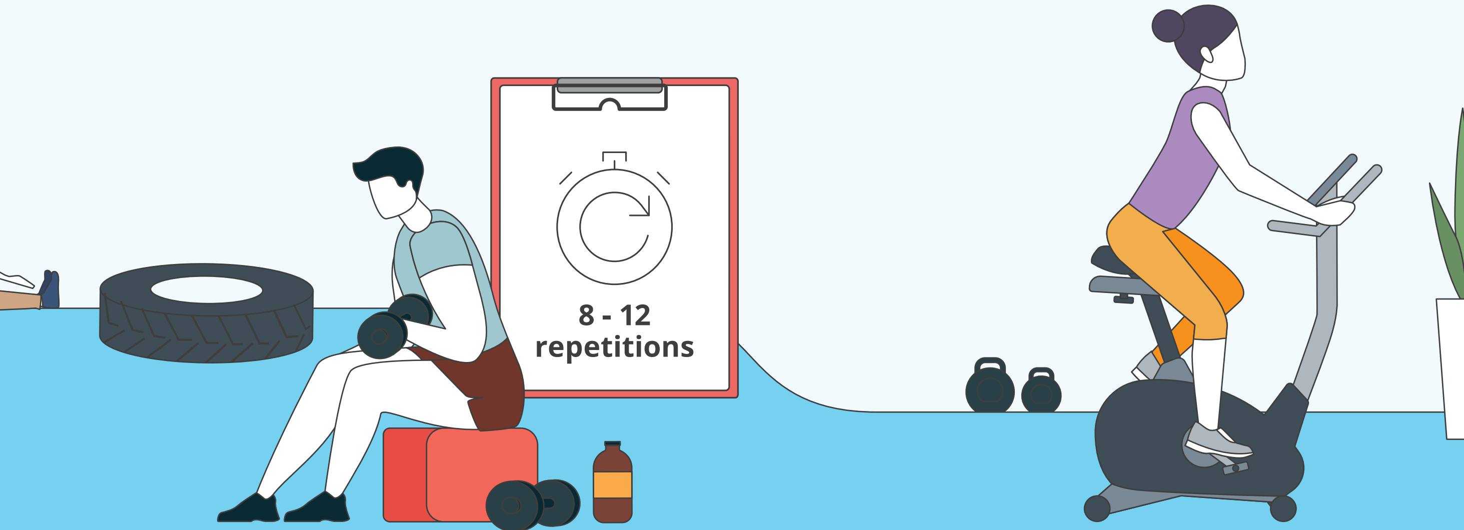

SINGAPORE PHYSICAL ACTIVITY GUIDELINES FOR ADULTS (18 - 64 YEARS)

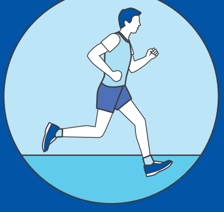

Adults are encouraged to engage in physical activity not only through recreation and leisure, but also when in different settings - At home (household chores), as a form of transportation (cycling, brisk walking, jogging), or in occupational and community settings.

MOVE MORE & THRIVE

RECOMMENDATIONS

GET MOVING AND MIX IT UP

Limit the amount of time spent being sedentary, particularly recreational screen time, by ***engaging in activity of any intensity***.

HIT 150 – 300

Aim for at least ***150 to 300 minutes*** of moderate-intensity aerobic physical activity per week.

*Every minute of vigorous-intensity activity can generally be considered as almost two minutes' worth of moderate-intensity activity.

150-300 minutes

GET STRONGER

Strengthen muscles, bones, and joints through activities at moderate- or vigorous-intensity, ***at least 2 days per week***.

For those over 50 years, include ***multi-component physical activity that emphasizes strength and functional balance at least 3 days of the week*** at a moderate or greater intensity.

For adults with chronic conditions (e.g. Type-2 Diabetes and Hypertension), the recommendations and tips are still applicable.

If unsure of how to begin, consult a health professional to determine a suitable exercise regime for you.

TIPS

- Incorporate active travel into your daily routine, such as heading out to purchase your food and groceries personally and rely less on food deliveries.
- Start with some light-intensity activity as opposed to being inactive.
- Work out your muscle groups through different activities such as jogging, swimming, and playing a racquet sport.
- Start with small changes like taking the stairs instead of the lift, as better beats perfect.
- Being active can be enjoyable - try a new sport or explore one of the many local parks.

EXERCISE INTENSITY

Light
Can talk in full sentences and sing

Moderate
Can talk in phrases or short sentences but cannot sing

Vigorous
Have difficulty talking

EXAMPLES OF AEROBIC & MUSCLE STRENGTHENING ACTIVITIES BY TYPE

AEROBIC ←	MULTICOMPONENT	→ MUSCLE STRENGTHENING
Jogging	Cycling	Tai Chi
Leisure Dancing	Kayaking	Pilates
Rope Skipping	Circuit Training	Weight Training
Running	Racquet Sports	
	Swimming	

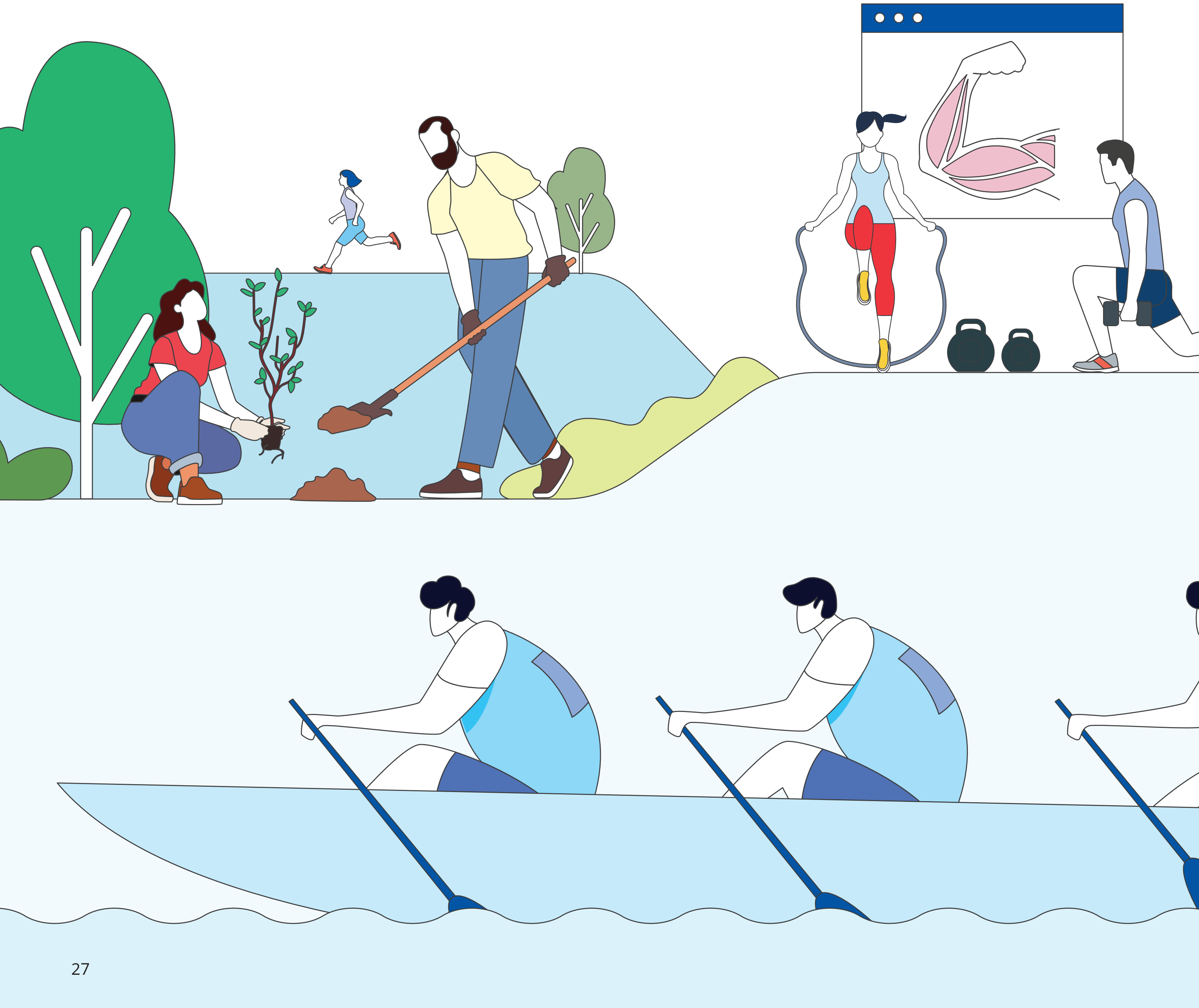

SUMMARY OF EVIDENCE: ADULTS

SEDENTARY BEHAVIOUR

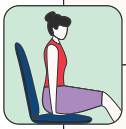

CARDIOVASCULAR DISEASE:

High levels of sedentary behaviour, even in healthy individuals, are associated with an increasing risk of developing cardiovascular disease.

SLEEP:

High levels of sedentary behaviour, especially prolonged periods of sitting screen time, is associated with lower quality of sleep, impacting mood, cognitive and physical performance.

TYPE-2 DIABETES:

Relatively high amounts of sedentary time have been associated with significantly greater risk for Type-2 Diabetes.

MENTAL HEALTH:

Evidence suggests that higher levels of sedentary behaviour results in increased frequency and severity of anxiety and depressive symptoms.

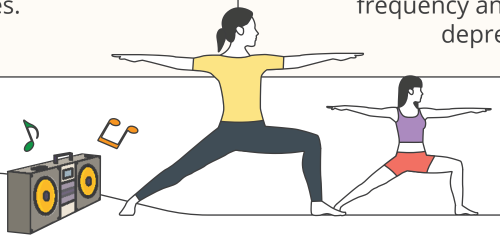

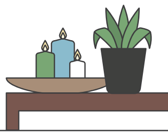

AEROBIC ACTIVITY

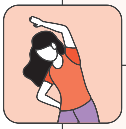

BLOOD PRESSURE & HYPERTENSION:

Aerobic activity reduces blood pressure in pre-hypertension adults, and reduces incident hypertension, both key risk indicators of cardiovascular diseases.

CANCER:

Higher levels of physical activity are associated with a reduced risk (10-20%) of multiple cancers such as colon, breast, bladder, liver, renal and more.

TYPE-2 DIABETES:

Inverse curvilinear relationship between physical activity and incidence of Type-2 Diabetes, with no significant difference by ethnicity and weight status.

MENTAL HEALTH:

Higher levels of aerobic activity was associated with a 17% lower incidence of anxiety and 24% lower incidence of depression among adults.

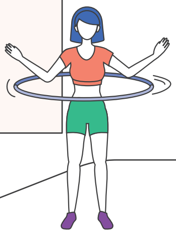

VIGOROUS-INTENSITY AND MUSCLE/BONE STRENGTHENING ACTIVITIES

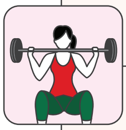

BODY COMPOSITION:

Resistance training increases lean muscle mass and resting metabolic rate, aiding fat loss in adults, and reducing the rate of muscle loss.

BONE HEALTH:

Resistance training increases bone mineral density in adults, which helps to reduce incidence of lower back pain and mitigate symptoms of arthritis.

COGNITIVE FUNCTION:

Muscle-strengthening activity is associated with improvements in cognition (memory and processing speed) and lowered the risk of Alzheimer's disease.

MENTAL HEALTH:

Resistance training and yoga are correlated with improvements in sleep quality and mental health indicators, such as reducing symptoms of anxiety and depression.

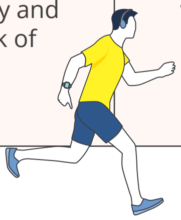

CASE STUDIES: ADULTS

CASE STUDY #1

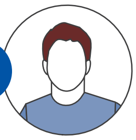

SHAFIQ, 24

Transitioning from student to full-time work

Shafiq led an active lifestyle when he first entered university and took part in many sporting events. As he had just finished his National Service, he was determined to maintain his fitness level while juggling schoolwork. During his free time, he and his peers would play football at the school field.

As Shafiq transits to a full-time working adult, he finds himself struggling to manage his time and to strike a balance between work and social life. He often works overtime on weekdays and occasionally on weekends too. When his friends invite him to dinner, he is often too tired from work and would rather stay home to browse social media or watch shows online. He has realised that his ability to perform basic movements has deteriorated in recent years due to his inactivity. Despite his busy schedule, Shafiq enjoys football and finds time to join his friends on some weekends for a few games. At times, Shafiq will also go for an evening jog if he feels overwhelmed by work as he finds it to be helpful in clearing his mind and reducing stress levels.

Shafiq has been doing a good job in trying to stay fit despite his busy schedule. By using pockets of time to clear his mind through exercise and playing football with friends, he can stay connected while keeping fit. For individuals like Shafiq, always remember to:

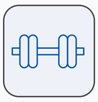

Find the time to incorporate workouts into your routine, for example, replace 30 minutes of social media time with a quick strength training session.

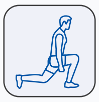

Bite-sized movement snacks during the workday can help improve systemic circulation and energise you after long bouts of sedentary behaviour.

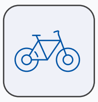

Make full use of your travelling time by cycling to nearby places to get a quick workout.

CASE STUDY #2

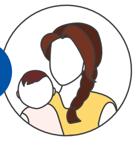

AMANDA, 37

Working mother with 2 young children

As a parent with young children, Amanda usually prioritises her family's needs before her own, such as ferrying her children around and completing household chores. With her busy work schedule, she has little time for exercise. However, Amanda learnt about High Intensity Interval Training (HIIT) workouts which can help her achieve more in less time. She started doing HIIT during her free time and eventually incorporated a quick 20-min HIIT workout into her day. With HIIT, Amanda also included weights training and overtime, she noticed that her usual chores were not as tiring as she feels physically stronger. Even on days that are busy, Amanda still does a 20-min workout every other day because it has become something she enjoys, and it helps her feel more ready for the day. On weekends, Amanda also tries to engage in activities with her family, such as exploring local nature trails or cycling at the park connectors.

It is great to hear that Amanda has found an enjoyable way to meet the recommended level of physical activity despite her busy schedule. For working mothers who would like to do the same, try the following:

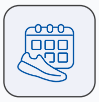

Attend weekly exercise programmes while incorporating muscle strengthening exercises at least 2 days a week.

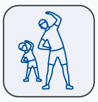

Get your children involved and exercise together during the weekends for a good way to stay active while bonding at the same time.

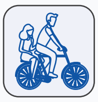

Ride your scooter or bicycle when doing the pick-up or drop-off to move as a family on school days.

CASE STUDIES: ADULTS

CASE STUDY #3

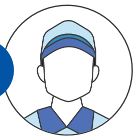

NICHOLAS, 48

Delivery man with irregular working hours

Nicholas's job as a delivery man is physically demanding. He delivers parcels from the warehouse to different locations, requiring him to lift items of various sizes and weights and walking short distances to deliver the items (i.e., from the van to the apartment). He usually takes the lift to higher floors but sometimes chooses to take the stairs for deliveries to lower floors to save time.

As his work hours are irregular (working on both weekdays and weekends), he does not have a fixed exercise routine and uses random pockets of time to go fishing with his friends. On his day off, he meets up with his friends at the coffeeshop for meals and drinks, where he can enjoy some downtime and relieve stress from work. Apart from this, he spends most of his off days resting at home.

Climbing stairs while making deliveries is a good practice to get an aerobic workout. Being a delivery man, he is already getting work-related physical activity in his daily routine. He may benefit from some strength, mobility, and flexibility exercises to enhance his quality of movement and build strength. If you're like Nicholas and have irregular working hours, here are some tips for individuals to achieve a healthier lifestyle.

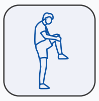

Incorporate mobility and flexibility movements throughout the day to increase your systemic blood circulation and help the overworked joints and muscles feel better.

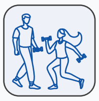

Try playing a sport with your friends before going for a meal together. By doing so, you get to spend quality time plus keep fit and relieve stress.

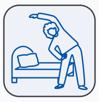

If you are exercising close to bedtime, try keeping to a light- or moderate-intensity exercise to avoid disruptions to your sleep.

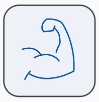

Incorporate muscle-strengthening exercises at least 2 days a week to combat age-related muscle loss.

CASE STUDY #4

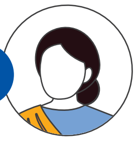

NISHA, 58

Retired, recently diagnosed with Type-2 Diabetes

Nisha is a hotel manager who is recently retired. She mindfully slows down her pace of life by revisiting old hobbies. She conducts part-time cooking classes at the nearby Community Centre and volunteers with a sports interest group as a local guide, occasionally organising community health and wellbeing events.

Unfortunately, Nisha was subsequently diagnosed with Type-2 Diabetes. She manages her condition by making gradual lifestyle changes like using healthier alternatives when cooking. She also keeps herself active by participating in a variety of community fitness classes such as zumba and yoga at least twice a week, and hikes with her interest group once a month. Nisha has always been motivated to learn more about taking ownership of her health, but her recent annual health check-up gave her a surprise. She learnt that she has low skeletal muscle mass and is at risk of sarcopenia. Worried and puzzled, Nisha hopes to understand why she is at-risk despite being active and how she can combat age-related muscle loss.

Nisha is a role model for her efforts in incorporating healthy practices into her daily life. Here are some other helpful tips that could benefit Nisha and others in a similar situation:

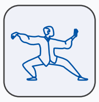

You can protect yourself from sarcopenia through more muscle- and bone-strengthening activities such as stretch band classes, aqua aerobics, and Tai Chi.

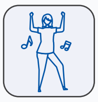

Participate in dance classes, such as zumba, at higher frequencies and alternate between dance activities and strengthening exercises, such as Active Health workshops aimed to combat age-related loss of muscles.

GUIDELINES BY SUB-POPULATION: PREGNANT & POSTPARTUM WOMEN

UPDATES IN THE SINGAPORE PHYSICAL ACTIVITY GUIDELINES (SPAG): PREGNANT & POSTPARTUM WOMEN

WHAT'S NEW

Encourage physical activity for pregnant and postpartum women without contraindications.

Guidelines for the adjustments to physical activity during and after pregnancy.

Safety considerations of physical activity for pregnant and postpartum women.

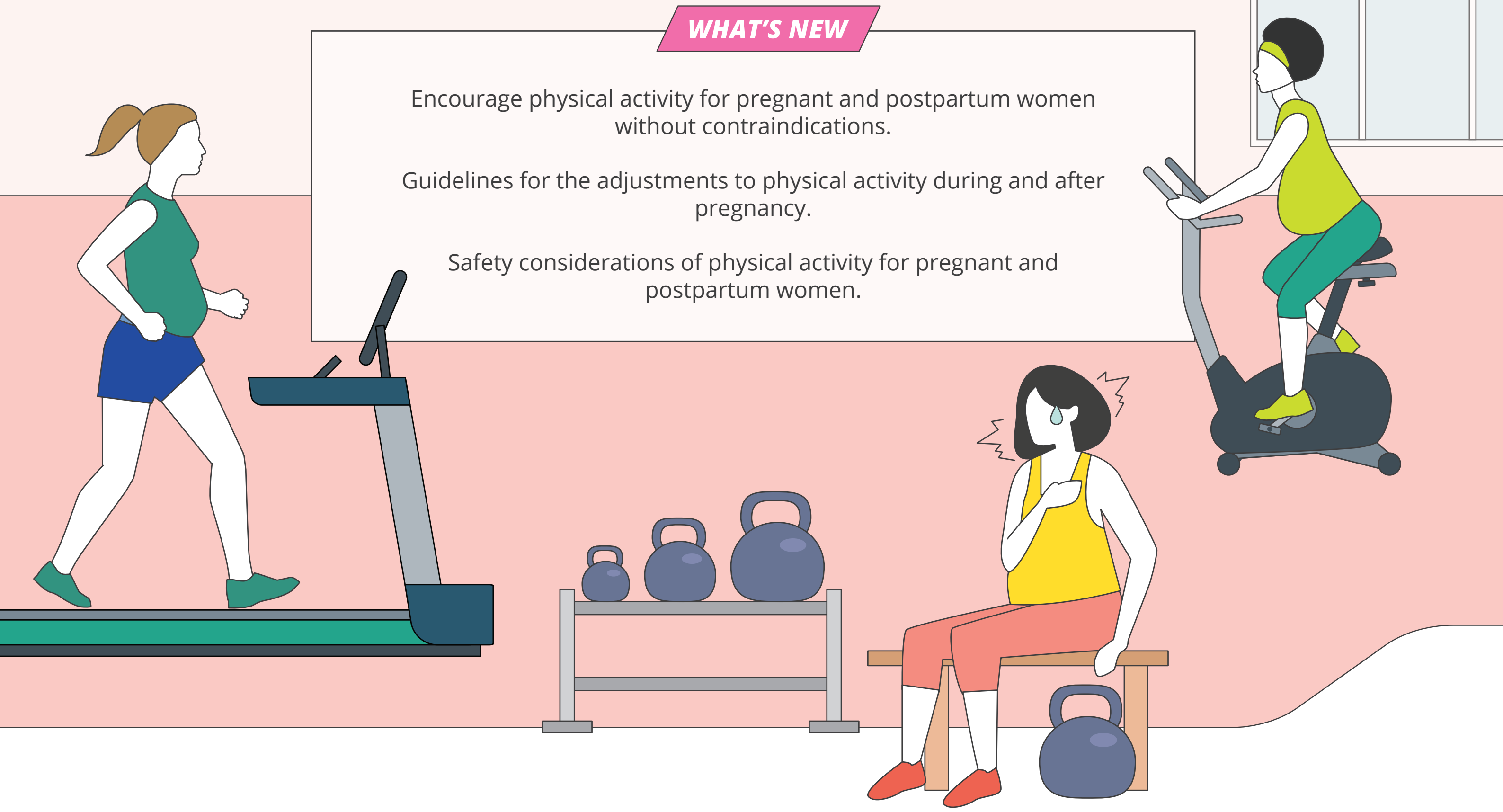

IT IS RECOMMENDED THAT...

A variety of aerobic, muscle-strengthening and light stretching exercises should be incorporated during pregnancy and the postpartum period.

Pregnant and postpartum women should avoid vigorous-intensity physical activity.

SAFETY CONSIDERATIONS

- Avoid excessive heat
- Avoid contact sports and exercises with high fall risks
- Ease back into physical activity gradually
- Consult your doctor when needed

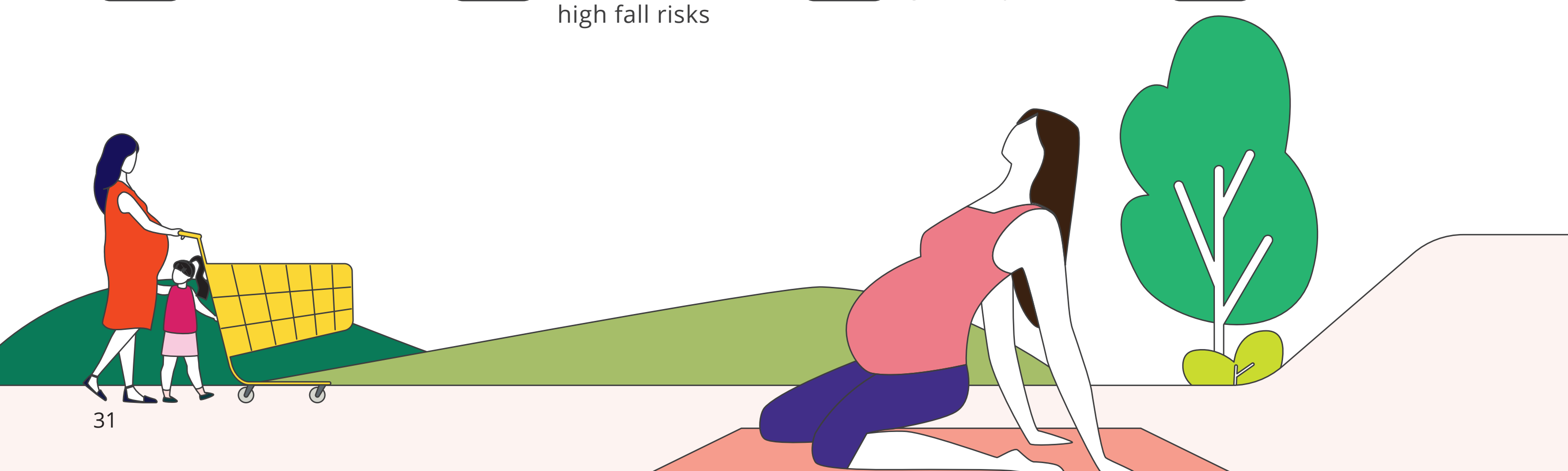

SINGAPORE PHYSICAL ACTIVITY GUIDELINES FOR PREGNANT & POSTPARTUM WOMEN

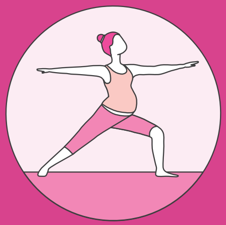

Pregnant and postpartum women without contraindications are encouraged to engage in physical activity not only through recreation and leisure, but also when in different settings - At home (light household chores), as a form of transportation (cycling, walking, jogging), or in occupational and community settings.

MOVE WITH CARE & NURTURE

RECOMMENDATIONS

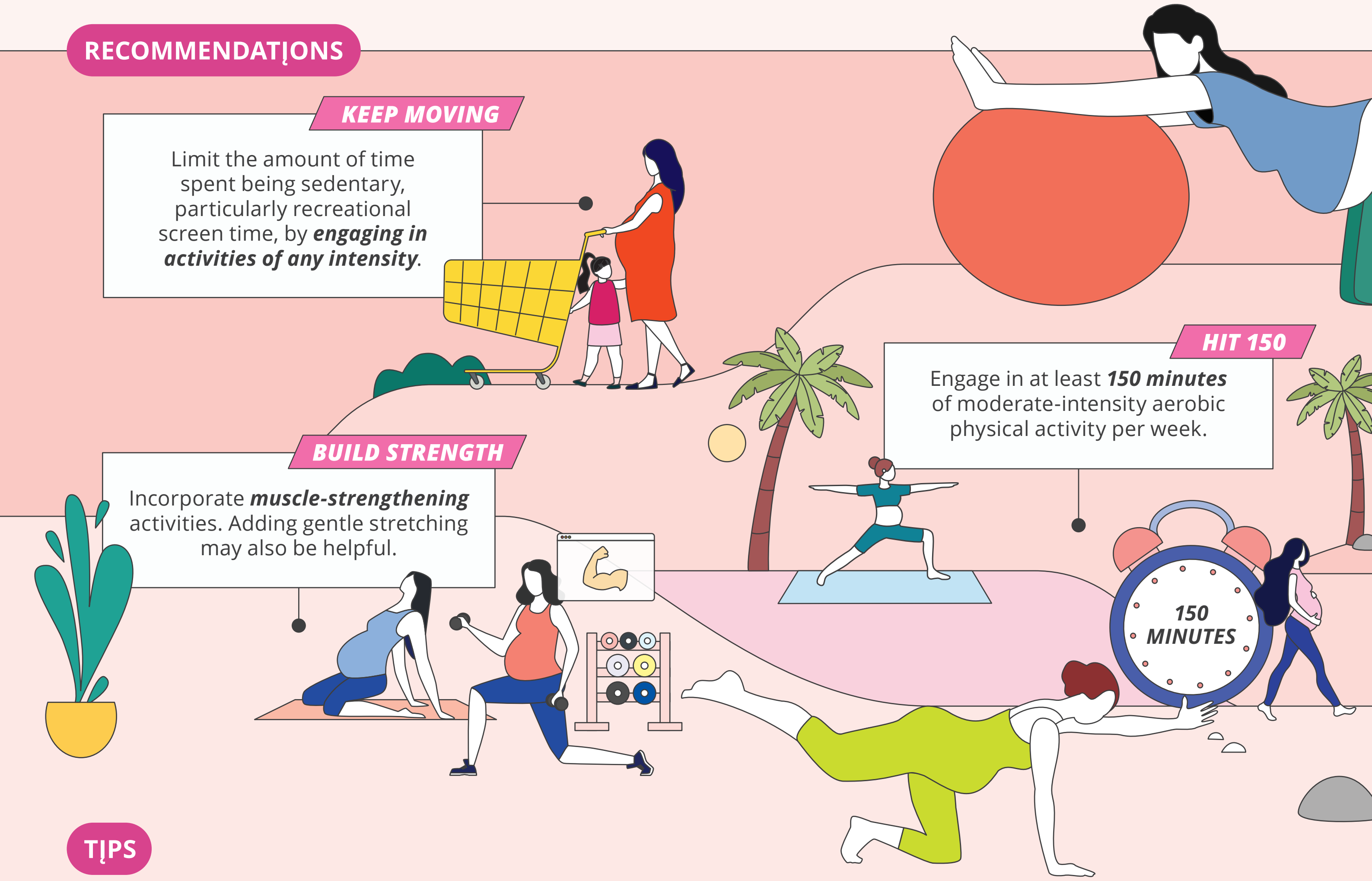

KEEP MOVING

Limit the amount of time spent being sedentary, particularly recreational screen time, by ***engaging in activities of any intensity***.

HIT 150

Engage in at least ***150 minutes*** of moderate-intensity aerobic physical activity per week.

BUILD STRENGTH

Incorporate ***muscle-strengthening*** activities. Adding gentle stretching may also be helpful.

TIPS

- Doing some physical activity is better than none.
- Ease into your physical activity routine progressively. Listen to your body and seek your doctor's recommendation on suitable activities if you are unsure of how to start.
- Core strengthening activities and pelvic muscle training may be performed regularly to strengthen the trunk and reduce the risk of urinary incontinence.
- Start by doing small amounts of physical activity, and gradually increase frequency, intensity and duration over time.
- Avoid participating in activities which involve physical contact or a higher risk of falling.

EXERCISE INTENSITY

Light
Can talk in full sentences and sing

Moderate
Can talk in phrases or short sentences but cannot sing

Vigorous
Have difficulty talking

RECOMMENDED ADJUSTMENTS OF PHYSICAL ACTIVITY DURING & AFTER PREGNANCY

Women who have been engaged in light- to moderate-intensity physical activity before pregnancy should continue this regime during pregnancy and adjust the intensity of exercises according to their bodies during the postpartum period.

Women who have been physically active or who have engaged in vigorous-intensity aerobic activity can continue these activities during pregnancy and during the postpartum period.

INDIVIDUAL

BEFORE PREGNANCY

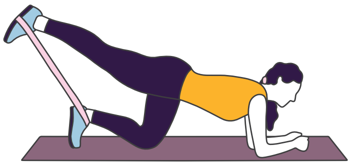

Commit to an active lifestyle by following the recommendations for an adult.

Light- to moderate-intensity
E.g.: Pilates, jogging, swimming

Vigorous-intensity
E.g.: Running, floorball, basketball, netball

DURING PREGNANCY

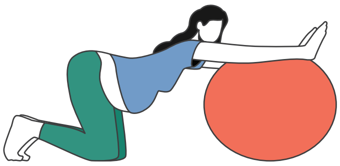

Reduce intensity of activity, keep at moderate-intensity. Avoid contact sports and risk of falls.

E.g.: Elliptical, yoga, jogging, swimming

AFTER PREGNANCY

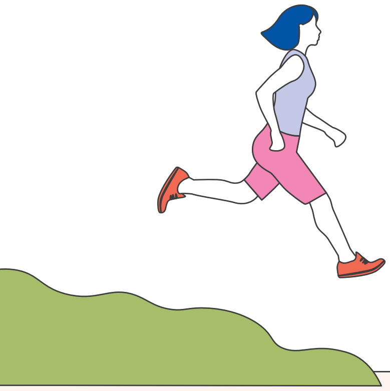

Listen to your body and ease into pre-pregnancy exercise regime gradually, building up intensity progressively.

Low-intensity
E.g.: Walking, light stretching

Moderate-intensity
E.g.: Brisk walking, post-natal yoga, swimming

Vigorous-intensity
E.g.: Floorball, running, basketball, netball, circuit classes

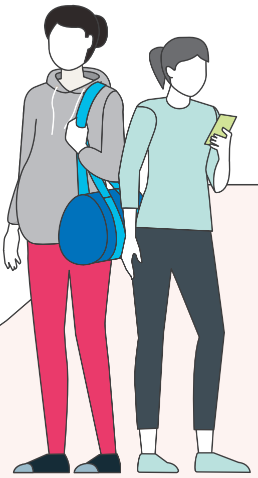

REMEMBER

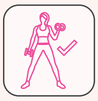

It is safe to be active postpartum

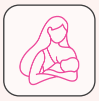

You can be active whilst breastfeeding

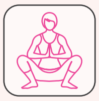

Start pelvic floor exercises early to strengthen your muscles

SUMMARY OF EVIDENCE: PREGNANT & POSTPARTUM WOMEN

PREGNANT WOMEN

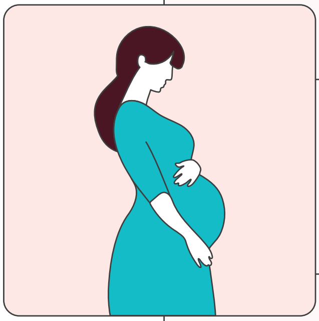

GESTATIONAL WEIGHT GAIN:

Engaging in physical activity during pregnancy is significantly associated with reduced gestational weight gain.

GESTATIONAL DIABETES:

Engaging in physical activity before and during pregnancy is associated with a reduced risk of gestational diabetes.

PREMATURE BIRTH:

Women who engaged in vigorous-intensity physical activity had a small but significantly reduced risk of prematurity.

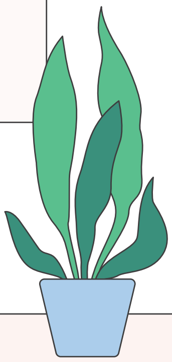

POSTPARTUM WOMEN

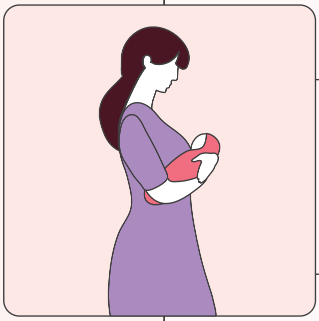

POSTPARTUM DEPRESSION:

Evidence demonstrates that physical activity during pregnancy may reduce postpartum depression.

POSTPARTUM WEIGHT GAIN:

Regular physical activity can help minimise weight gain in the postpartum period.

RECOVERY:

Engaging in physical activity after childbirth can increase the speed of musculoskeletal recovery.

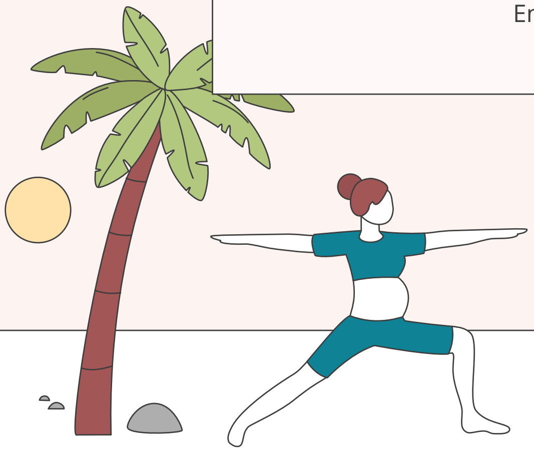

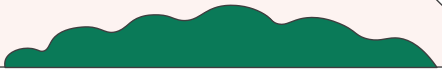

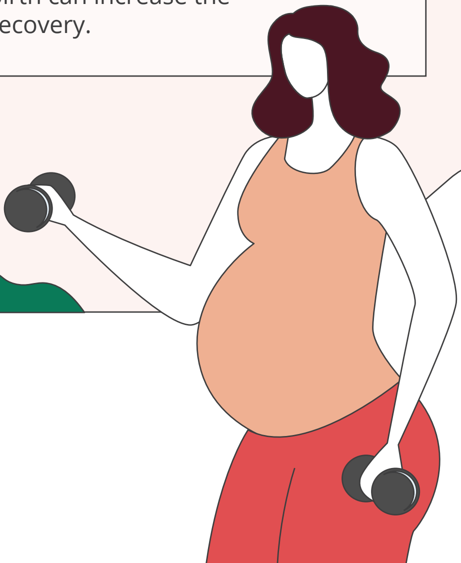

CASE STUDIES: PREGNANT & POSTPARTUM WOMEN

CASE STUDY #1

ISLA, 28

First-time mother who has always led an active lifestyle

Isla is 18 weeks pregnant. Before she got pregnant, Isla enjoyed an active lifestyle where she engaged in a variety of workouts, ranging from boxing to spin classes during her leisure time. She is motivated to continue being physically active, so she keeps her doctor informed of her activities throughout her pregnancy. She used to go for long hikes and continued to do so in her 1st trimester. As her pregnancy progressed, she slowly adapted to her body's condition and replaced hikes with daily 30-minute walks in the park. Isla also changed her weekly workouts to resistance band exercises to strengthen her muscles. Each weekend, Isla will attend a prenatal yoga session for additional strength training and to learn some simple exercises that she can safely do at home. Isla is looking forward to resuming her physical activity and trying out postnatal yoga after her delivery, once her doctor deems it is safe for her to do so.

Isla has adopted a great mindset of maintaining an active lifestyle throughout pregnancy. For expectant mothers like Isla, it is important to note the following:

- Be mindful of exertions associated with exercise and pace yourself accordingly.
- Consider trying yoga to maintain body strength or activities like aqua aerobics for a low-impact workout.
- Due to Singapore's hot and humid climate, it is important to be mindful of your exercising environment as pregnancies make you more vulnerable to the heat.

CASE STUDY #2

JAIME, 32

Sedentary mother with an increased risk of Gestational Diabetes Mellitus

Ever since her first pregnancy, Jaime has been leading a more sedentary lifestyle as she juggles the demands of work and taking care of her child. Recently, Jaime learnt that she is expecting her second child and her doctor advised that it is important to start adopting an active lifestyle for herself and her child. Jaime was diagnosed with Gestational Diabetes Mellitus (GDM) during her first pregnancy and coupled with her current inactive lifestyle, she has an increased risk of it recurring.

Jaime goes for a 30-minute stroll only when she finds the free time between a full day of work and caring for her child. With her doctor's advice, she tries to gradually squeeze in a 15-minute workout during lunch time and attends a weekly prenatal yoga class on the weekends. She hopes to gradually build up her activity by consulting her doctor regularly to keep herself and her child healthy.

Jaime's attempt is commendable. For other mothers who are trying to do the same, here are some simple ways to build up your activity levels:

- Work closely with your doctor to come up with an exercise regimen that includes aerobic activities and muscle-strengthening exercises.
- Incorporate activities like Pilates, yoga, and resistance band exercises to maintain muscle mass and aid in glucose control.
- Try swimming for a low-impact full body workout.
- Plan for pool days or going for long strolls at the park with your family as a workout and spend quality time together.

GUIDELINES BY SUB-POPULATION: PERSONS WITH DISABILITIES

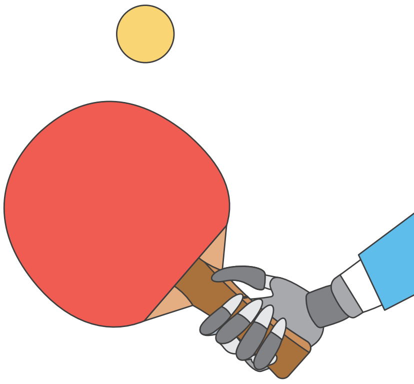

UPDATES IN THE SINGAPORE PHYSICAL ACTIVITY GUIDELINES (SPAG): PERSONS WITH DISABILITIES

WHAT'S NEW

Encourage physical activity for persons with disabilities

Safety considerations of physical activity for persons with disabilities

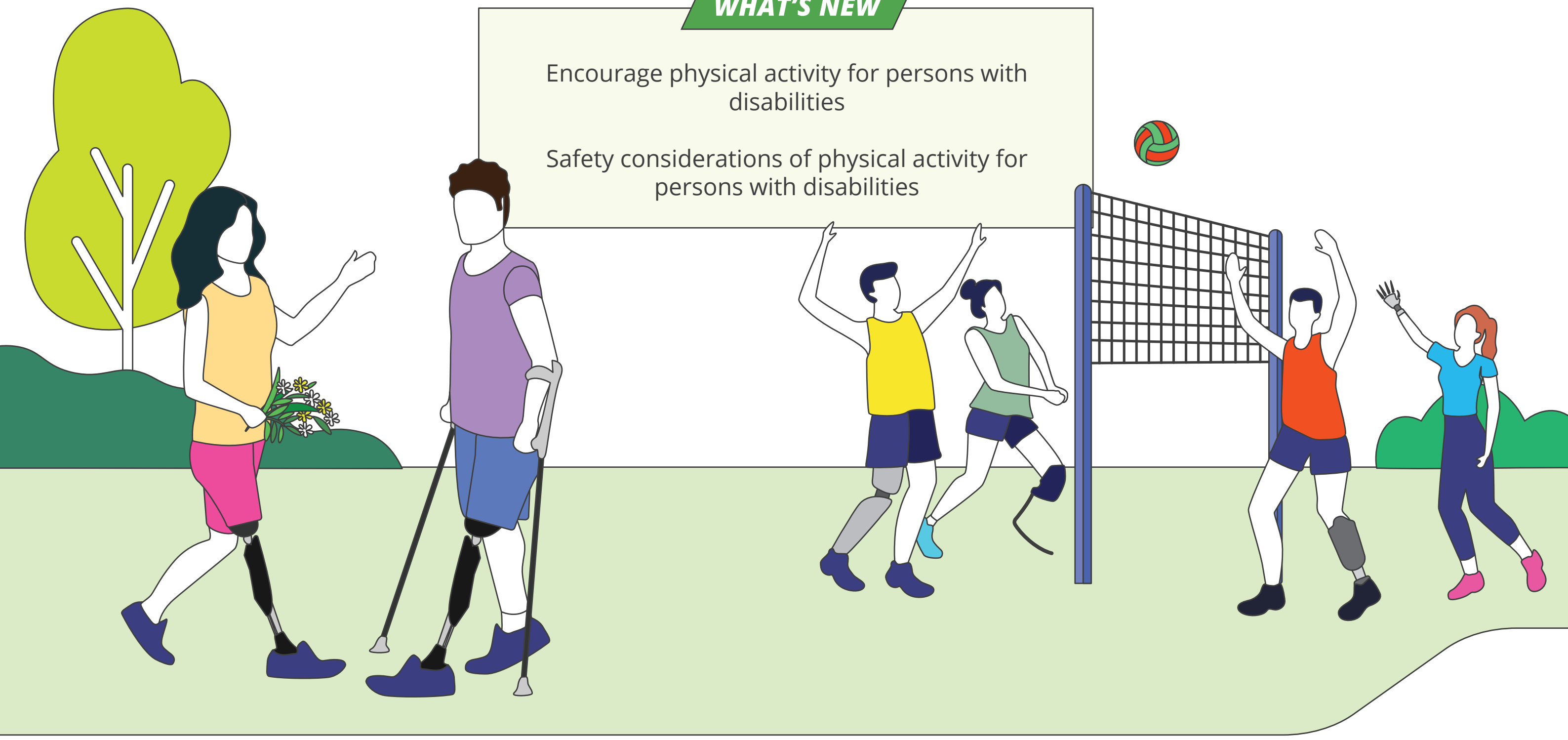

IT IS RECOMMENDED THAT...

Adults with disabilities who are not able to meet the key guidelines should avoid inactivity and engage in regular physical activity according to their abilities.

To participate in physical activities that are adapted to accommodate those with disabilities, for example, engage in basketball, badminton, tennis, and swimming.

Focus on finding and doing activities that you can enjoy, instead of avoiding all activities due to mobility and health concerns.

SAFETY CONSIDERATIONS

- Participate in suitable activities that are safe
- Always adopt a phased approach at the start
- Seek doctor's recommendations on suitable physical activities
- Supervision may be needed at times

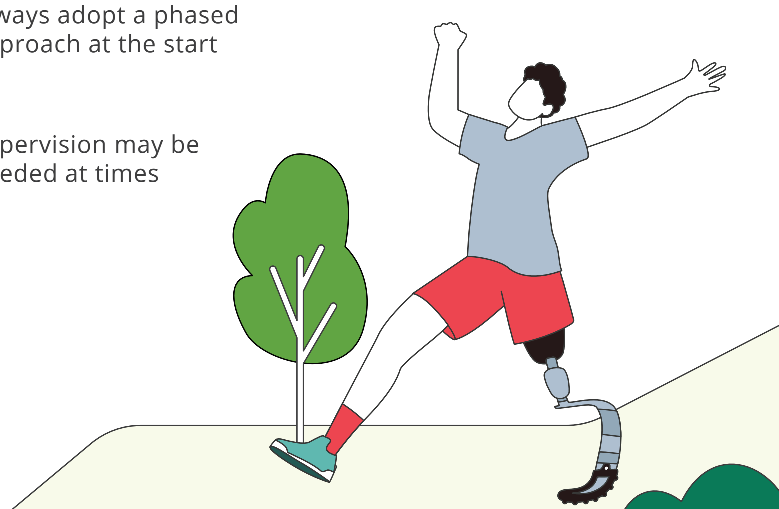

SINGAPORE PHYSICAL ACTIVITY GUIDELINES FOR PERSONS WITH DISABILITIES

Persons with disabilities can achieve important health benefits from physical activity. They should try to meet these recommendations where possible and as able.

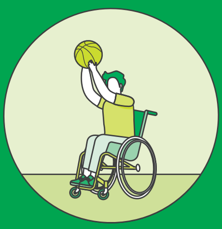

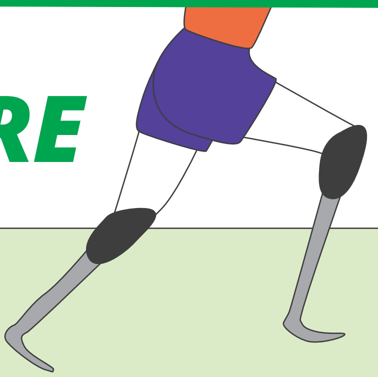

MOVE TOGETHER & INSPIRE

RECOMMENDATIONS

GET STRONGER

Strengthen muscles, bones, and joints through activities ***at least 2 days a week***.

KEEP MOVING

Aim to ***engage in physical activities every day***, including light-intensity activities.

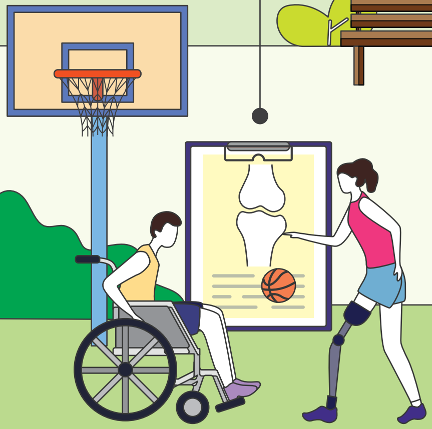

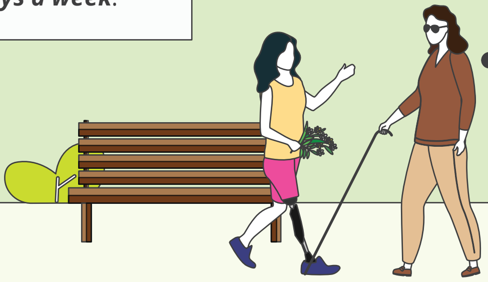

HIT 150 - 300

Aim for at least ***150 to 300 minutes of moderate-intensity aerobic physical activity per week***.

*Every minute of vigorous-intensity activity can generally be considered as two minutes' worth of moderate-intensity activity.

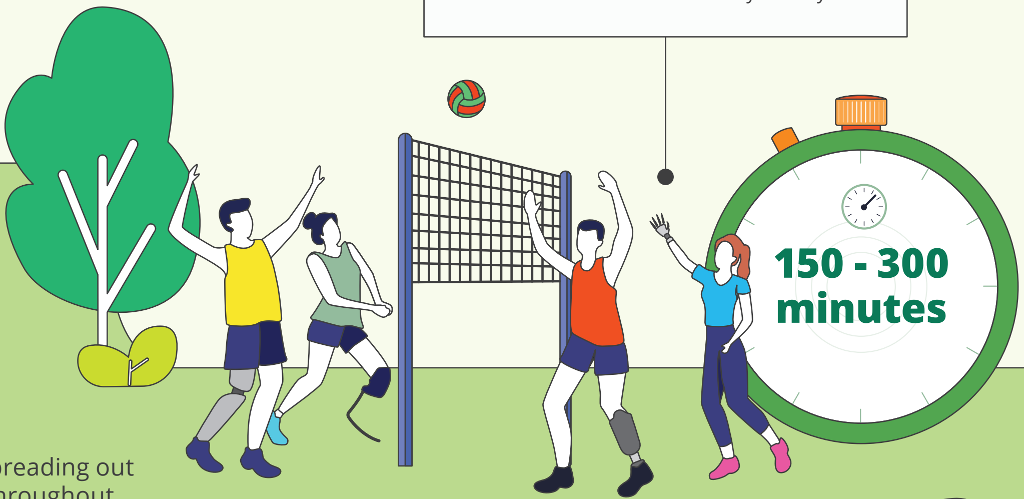

TIPS

- Avoid staying sedentary by spreading out some light-intensity activity throughout the day.
- Turn workouts into social events by reaching out to your friends and family to exercise together for extra support and motivation.
- Seek doctor's recommendations on suitable physical activities if unsure on how to start.
- Take a phased approach if necessary – gradually increasing the complexity and intensity of your activities.
- Listen to your body, reduce the intensity of your activities and adapt accordingly to prevent overexertion.

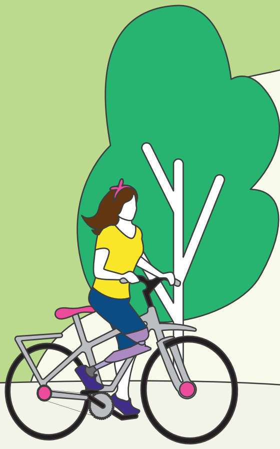

EXERCISE INTENSITY

Light
Can talk in full sentences and sing

Moderate
Can talk in phrases or short sentences but cannot sing

Vigorous
Have difficulty talking

SUMMARY OF EVIDENCE: PERSONS WITH DISABILITIES

DISABILITIES

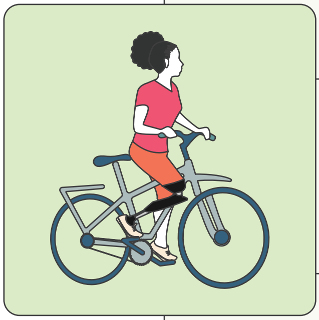

PHYSICAL FUNCTION:

Physical activity can improve motor symptoms, functional mobility and performance, endurance and velocity of forward and backward movement.

MENTAL HEALTH:

Increasing functional independence and ability helps to increase the confidence and mental health of those with physical disabilities.

COGNITIVE FUNCTION:

Physical activity can have beneficial effects on cognition, working memory, social cognition, and attention/vigilance.

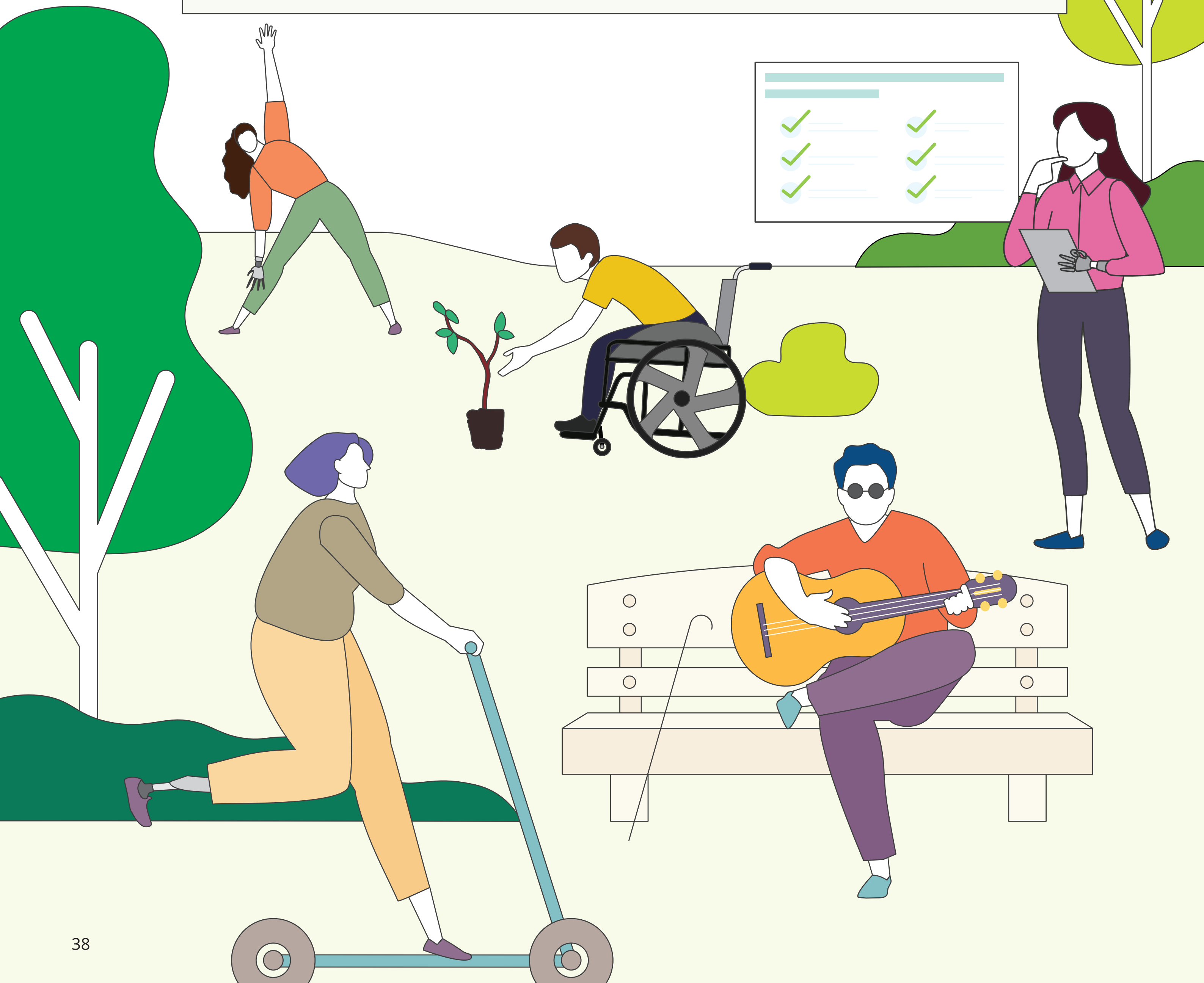

CASE STUDIES: PERSONS WITH DISABILITIES

CASE STUDY #1

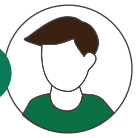

JAVEN, 16

Enjoys playing wheelchair basketball despite a spinal cord injury

Javen is an active teenager. On weekday mornings and evenings, he clocks in additional wheel time at the park connectors. He has always been an active boy and knows that he sleeps better after higher levels of activity in the day. Javen also engages in structured leisure-time physical activity once a week in the form of an organised wheelchair basketball game that lasts for 120 minutes.

In addition to the aerobic activity that he is exposed to in his basketball games and daily time spent at the park connector, Javen goes to the ActiveSG Enabling Village once a week to do some upper body strength exercises. Javen has a great social network, and his friends are also very active and inclusive, so he gets other opportunities throughout the week to be active and involved in sporadic bouts of physical activity with his buddies.

Javen has found the right balance of light, moderate- and vigorous-activities incorporated into his weekly routine. For Persons with Disabilities, it is important to:

- Combine strength and cardiorespiratory activities
- Include some light mobility and flexibility exercises to ensure proper recovery and minimise the risk of injury

CASE STUDY #2

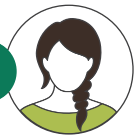

SERENE, 17

Diagnosed with an intellectual disability and does not have many opportunities for physical activity

Serene is in her final year of school. Her day is structured to include regular physical activity with the support of trained teachers for those with special needs. Serene's parents are concerned that she will not be able to remain active and stay healthy when the school term ends. To help Serene incorporate physical activity into her routines outside the school, they purposefully bring Serene out for a 30-minute light-intensity walk in the park every weekday evening. At home, Serene's mother gets Serene to help with some of the housework. This usually takes 60 minutes on weekends. Serene's mother gets her to play more organised sports and have recently signed her up to try adapted badminton.

Serene and her parents are making a great effort to help Serene stay physically active. Her family should also aim to get Serene to engage in more moderate-intensity activities during her evening walks and when doing housework.

- By including sport like adapted badminton into Serene's weekly routine, it will allow her to get the physiological benefits of physical activity and provide her with an opportunity to form social connections with others while having fun

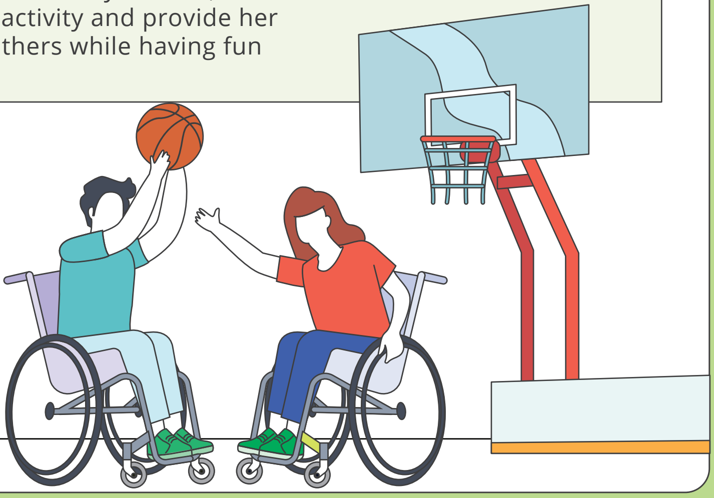

GUIDELINES BY SUBPOPULATION: OLDER ADULTS

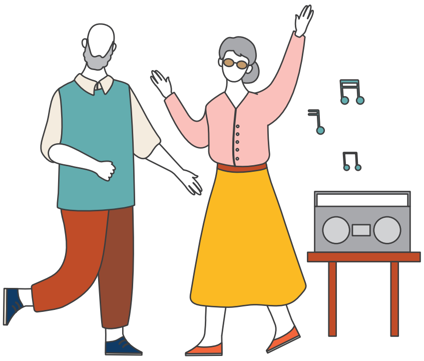

UPDATES IN THE SINGAPORE PHYSICAL ACTIVITY GUIDELINES (SPAG) Older Adults (65 YEARS & ABOVE)

Previous (2011)	What's New
Break up sedentary periods of 90+ minutes with 5-10 minutes of physical activity.	Removal of time-specific recommendations.
Minimum bouts of 10 minutes of physical activity per week.	No minimum bouts of physical activity.
Single time targets (e.g. 150 minutes).	Clocking in a target range e.g. 150 to 300 minutes of moderate-intensity aerobic physical activity.
No minimum intensity for muscle-strengthening activity.	Muscle-strengthening activities are recommended to be done at least 2 days a week, at moderate-intensity.
No recommendation on frequency of balance physical activity.<br><br>No functional training recommendation	Beyond balance training exercises to be done 3 days a week, functional training exercises are also recommended to be done at least 3 days a week.

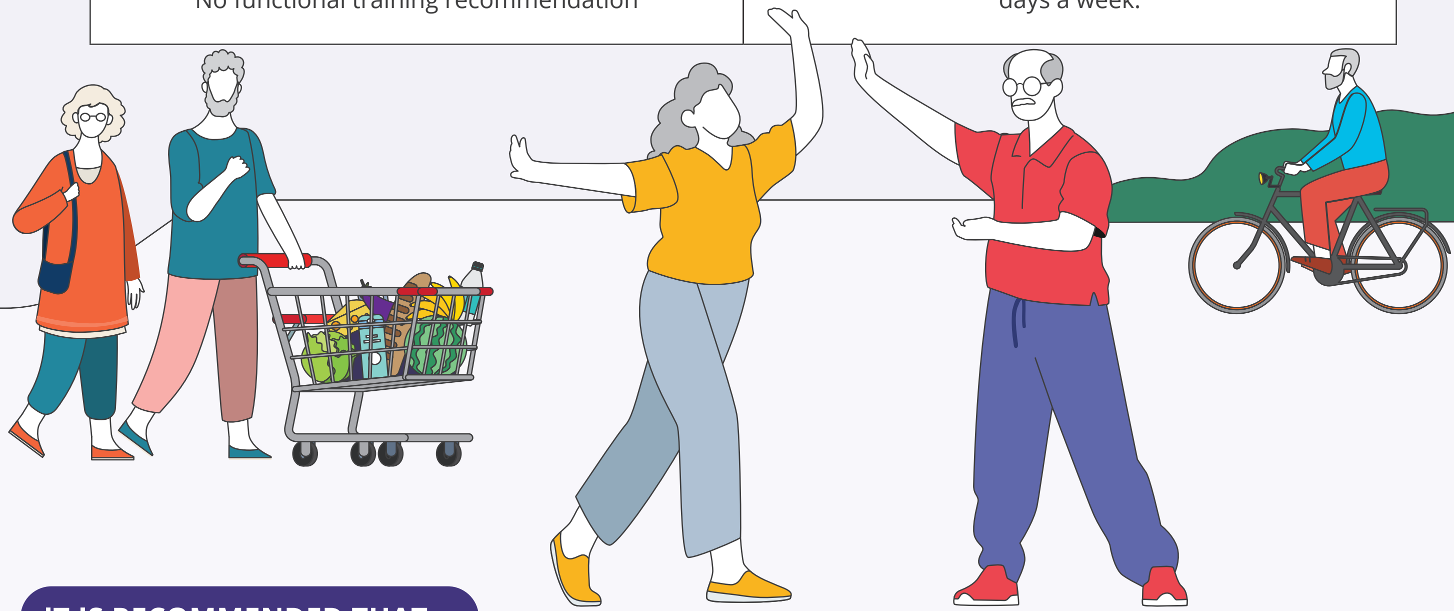

IT IS RECOMMENDED THAT...

Older adults should limit the amount of time spent sedentary, replacing that with physical activity of any intensity.

Older adults should engage in moderate-intensity physical activity daily, clocking in at least 150 to 300 minutes of moderate-intensity aerobic physical activity throughout the week.

Incorporate exercises aimed at improving or maintaining muscle strength, balance, and flexibility at least three days a week as part of the weekly physical activities.

SINGAPORE PHYSICAL ACTIVITY GUIDELINES FOR OLDER ADULTS (65 YEARS & ABOVE)

Older adults should engage in regular physical activity to improve overall wellbeing, enhance functional capacity, and prevent falls.

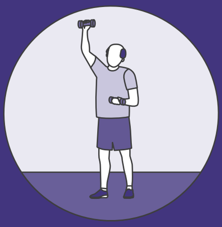

MOVE STRONG & BE BALANCED

RECOMMENDATIONS

RETAIN STRENGTH

Engage in muscle-strengthening activities on 2 or more days a week, at moderate or greater intensity, to keep muscles, bones, and joints strong.

Include ***multi-component physical activity that emphasizes strength and functional balance at least 3 days of the week*** at a moderate-intensity or greater.

HIT 150 – 300

Aim for at least ***150 to 300 minutes of moderate-intensity aerobic physical activity per week***.

*Every minute of vigorous-intensity activity can generally be considered as two minutes' worth of moderate-intensity activity.

150-300 minutes

KEEP MOVING

Limit the amount of time spent being sedentary, particularly recreational screen time, by ***engaging in activity of any intensity***.

For adults with chronic conditions (e.g. Type-2 Diabetes and Hypertension), the recommendations and tips are still applicable.

If unsure of how to begin, consult a health professional to determine a suitable exercise regime for you.

TIPS

- Incorporate simple strength training exercises such as resistance band exercises when watching your favourite shows.
- Engage in varied multi-component physical activity at home or in a structured group setting, which can combine aerobic, muscle-strengthening, and balance training into a session.
- Walk or play sports with your family and friends; this is a great way to be active while spending quality time with your loved ones.
- Play and try different sports with friends at publicly available sport facilities.
- Make use of the outdoor fitness equipment around your community spaces or your built environment as you brisk walk around your neighbourhood.

EXERCISE INTENSITY

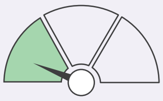

Light
Can talk in full sentences and sing

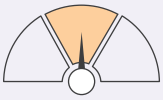

Moderate
Can talk in phrases or short sentences but cannot sing

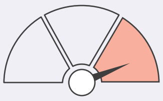

Vigorous
Have difficulty talking

EXAMPLES OF AEROBIC & MUSCLE STRENGTHENING ACTIVITIES BY TYPE

AEROBIC ←	MULTICOMPONENT	→ MUSCLE STRENGTHENING
**Brisk Walking**	**Cycling**	**Tai Chi**
**Leisure Dancing**	**Racquet Sports**	**Pilates**
**Jogging**	**Hiking**	**Weight Training**
**Rope Skipping**	**Swimming**	
	**Circuit Training**<br>*Circuit training for older adults is usually performed at a light- to moderate-intensity and helps to build muscular endurance and cardiorespiratory fitness.*	

PHYSICAL ACTIVITY IS IMPORTANT IN TACKLING THE FOLLOWING HEALTH CONSIDERATIONS THAT CAN IMPACT THE FRAILTY OF OLDER ADULTS.

JOINT COMPLICATIONS

Physical activity helps improve physical function following a hip fracture, improve walking and performance-based measures of gait, balance, strength, and mobility.

FUNCTIONAL PERFORMANCE

Regular physical activity is safe and beneficial for those with limitations to improve functional ability and ability to do everyday activities.

OVERWEIGHT & OBESITY

To keep within a healthy weight range, use at least 350 calories per day in physical activity as well as the energy used in everyday tasks.

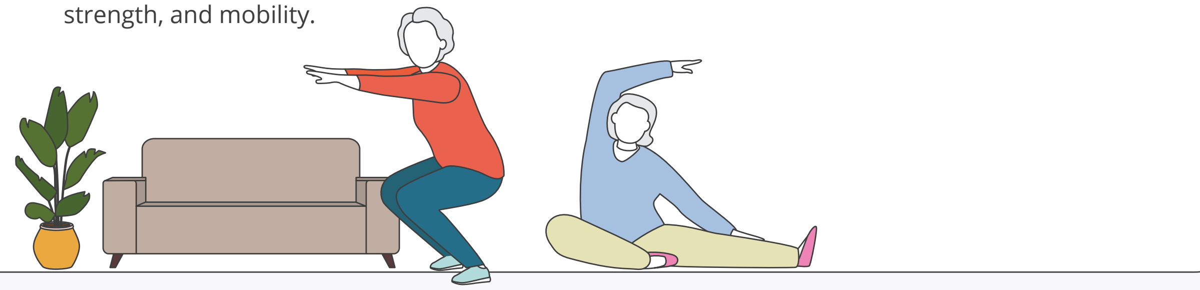

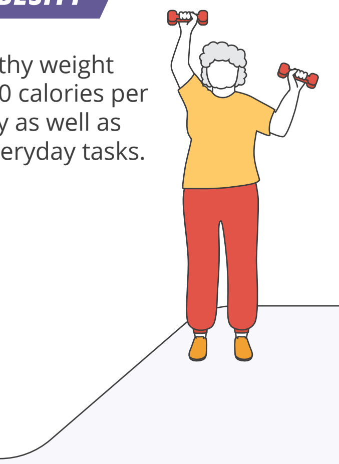

DIFFERENT ACTIVITIES OF VARYING INTENSITIES AND THEIR IMPACT ON MUSCLE FUNCTION, BALANCE, AND FLEXIBILITY

	INTENSITY	IMPROVEMENT IN MUSCLE FUNCTION	IMPROVEMENT IN BALANCE/FLEXIBILITY
**Walking**	Light to moderate	✓	
**Jogging**	Moderate to vigorous	✓	✓
**Swimming**	Moderate to vigorous	✓✓	
**Tai Chi**	Light to moderate	✓	✓
**Strength Exercises**	Moderate to vigorous	✓✓	
**Activities of Daily Living**	Light	✓	✓

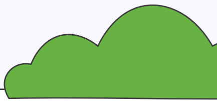

SCALE ✓ LOW ✓✓ MEDIUM

SUMMARY OF EVIDENCE

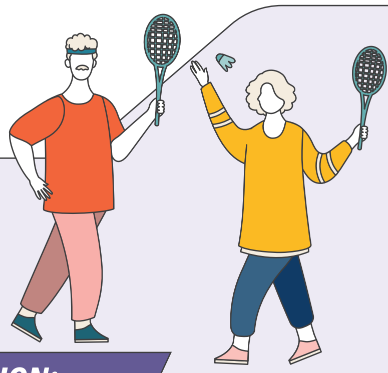

SEDENTARY BEHAVIOUR

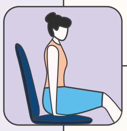

ALL-CAUSE MORTALITY:

Replacing sedentary or inactive behaviours with light-intensity activity reduces the risk of all-cause mortality, cardiovascular disease, and type-2 diabetes.

PHYSICAL FUNCTION:

Physical function has a linear relationship with mortality, and those with poor physical function have a higher risk of all-cause mortality, even from mid-life.

CARDIOVASCULAR DISEASE:

Prolonged sitting is associated with a higher risk of premature death and death from cardiovascular disease (CVD).

BONE HEALTH:

Evidence supports the benefits of being active throughout the day, such as better maintenance of bone health with more light intensity activity spread out through the day.

AEROBIC ACTIVITY

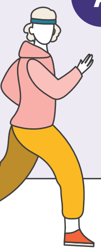

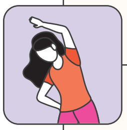

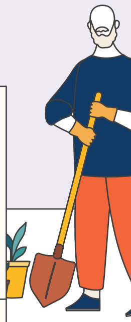

CANCER:

Evidence shows that doing more than 5 hours of moderate-intensity (or 2½ hours of vigorous-intensity) physical activity each week can help prevent and manage some cancers.

CARDIOVASCULAR DISEASE:

Physical activity causes beneficial short- and long-term autonomic and haemodynamic adaptations, resulting in a lower risk of hypertension, a key risk factor for CVD.

COGNITIVE FUNCTION:

Physical activity reduces the risk of cognitive impairment and dementia, and 30 minutes of moderate activity per day almost halves the odds of experiencing depression.

LIFE SATISFACTION:

Positive improvements in life satisfaction were observed for older adults with interventions including 50-minute classes taking place 3 times per week.

VIGOROUS-INTENSITY AND MUSCLE/BONE-STRENGTHENING ACTIVITIES

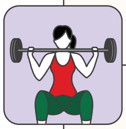

FALLS:

Exercise may reduce the rate of falls by as much as 23% in older adults, which can significantly reduce the risk of injury.

MENTAL HEALTH:

Evidence indicates that resistance exercise interventions have a beneficial effect on mental health, with reductions in depression and anxiety symptoms.

PHYSICAL FUNCTION & INDEPENDENCE:

Multi-component strength and balance activities, including flexibility, are key to improving physical function, helping to maintain functional independence.

BONE HEALTH:

Muscle-strengthening activities can be useful in managing osteoporosis and osteoarthritis.

CASE STUDIES: OLDER ADULTS

CASE STUDY #1

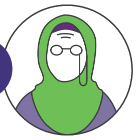

FATIMAH, 68

Recent retiree who is paying more attention to her health due to her age

Fatimah has been an avid walker for years. Recently retired, Fatimah continues to enjoy her daily 45-minute morning walks at Bishan-Ang Mo Kio Park. She also spends most of her time hanging out with her friends and playing with her grandchildren when they visit over the weekends. While Fatimah feels she is active, she is not as strong anymore. With her age, she is now more cautious when moving about as she worries about falling and injuring herself. She shared her concerns with the doctor whom she visits regularly for her blood pressure and cholesterol checks. Her doctor advised that, while frequent walking is a good start, doing strength exercises is just as important. Fatimah started trying out the various outdoor gym equipment at the park and noticed that she was gradually gaining strength to carry and play with her grandchildren. She also began feeling strong enough to hand carry her groceries when going to the market.

Fatimah has been successful in staying active by taking regular walks and playing with her grandchildren, so keep it up! If you are like Fatimah, you can build on your physical activity by increasing your activity intensity and strengthening your muscles.

- Try brisk-walking or cycling instead of walking.
- Make use of your park facilities, fitness corners and built environment.
- Hand carry your groceries.
- Improve your muscle strength and balance through physical activities; e.g. Tai Chi.

Strengthening your body can help you gain confidence and continue to play an active part in your grandchildren's life.

CASE STUDY #2

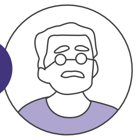

AH HUAT, 77

Have been leading a sedentary lifestyle as he does not want to put stress on his body at his age

Having retired for a while now, Ah Huat has a lot of spare time which is spent on watching television programmes, listening to radio, or taking naps when he starts feeling drowsy mid-day. As he has been mostly inactive for many years, he is starting to find it challenging with his daily activities. These include getting up from the toilet seat and climbing the stairs. Ah Huat still enjoys his old hobbies, like the occasional swim or joining his friends for fishing. On days when he heads out for an activity, he feels more energetic. However, Ah Huat has concerns about working his body frequently at his age. His arthritis condition causes him joint pains that make moving around increasingly difficult. This has further deterred him from doing the activities that he enjoys. As a result, he prefers to spend most of his time at home doing more relaxing activities that require less movements.

Going out with friends is a great way to maintain common interests and stay active together. If you are like Ah Huat, you should continue exploring different activities that you find enjoyable and suitable. Rather than avoiding exercise completely, why not try the following with friends and stay active together:

- Start slow with walking then gradually progress within your capability.
- Muscle-strengthening exercises like Tai Chi and stretch band exercises can to help alleviate some pain.

By staying active and improving your strength and balance, you not only gain better health, but confidence and the ability to perform daily tasks better. You can then continue doing the things you enjoy!

GLOSSARY OF TERMS

Term	Definition
Adiposity	A condition of being morbidly overweight or obese. Excessive fat accumulation in the body, as measured by BMI, BMI-for-age, BMI z-score, skinfold thickness, body fat mass.
Aerobic physical activity	Activity in which the body's large muscles move in a rhythmic manner for a sustained period. Aerobic activity – also called endurance activity – improves cardiorespiratory fitness. Examples include walking, running, swimming, and bicycling.
Balance training	Static and dynamic exercises that are designed to improve an individual's ability to withstand challenges from postural sway or destabilizing stimuli caused by self-motion, the environment, or other objects.
Body composition	The proportion of fat and fat-free mass in the body. This can be measured as percentage body fat.<br><br>Body mass index (see below) and waist circumference are proxies for body composition and in most situations, are good indicators of health risk associated with excess adiposity.
Body mass index (BMI)	Weight (kg) / height (m)2
Bone-strengthening activity	Physical activity primarily designed to increase the strength of specific sites in bones that make up the skeletal system. Bone-strengthening activities produce an impact or tension force on the bones that promotes bone growth and strength.<br><br>Running, jumping rope, and lifting weights are examples of bone-strengthening activities.
Cardiometabolic health	The interplay of blood pressure, blood lipids, blood glucose and insulin on health.
Cardiorespiratory fitness (endurance)	A health-related component of physical fitness. The ability of the circulatory and respiratory systems to supply oxygen during sustained physical activity. Usually expressed as measured or estimated maximal oxygen uptake (VO2 max).
Cardiovascular disease (CVD)	Cardiovascular diseases (CVDs) are a group of disorders of the heart and blood vessels. They include coronary heart disease, cerebrovascular disease, peripheral arterial disease, deep vein thrombosis and pulmonary embolism among others.
Cognitive function	Cerebral activities, i.e. reasoning, memory, attention, and language that lead to the attainment of information and knowledge. This can also include learning.

GLOSSARY OF TERMS

Term	Definition
Disability	From the International Classification of Functioning, Disability and Health (WHO), an umbrella term for impairments, activity limitations, and participation restrictions, denoting the negative aspects of the interaction between an individual (with a health condition) and that individual's contextual factors (environmental and personal factors).
Exercise	A subcategory of physical activity that is planned, structured, repetitive, and purposeful in the sense that the improvement or maintenance of one or more components of physical fitness is the objective. "Exercise" and "exercise training" frequently are used interchangeably and generally refer to physical activity performed during leisure time with the primary purpose of improving or maintaining physical fitness, physical performance, or health.
Executive function	Includes constructs such as: working memory, cognitive flexibility (also called flexible thinking) and inhibitory control (which includes self-control)
Fitness	A measure of the body's ability to function efficiently and effectively in work and leisure activities, and includes, for example, physical fitness and cardiorespiratory fitness.
Flexibility	A health- and performance-related component of physical fitness that is the range of motion possible at a joint. Flexibility is specific to each joint and depends on a number of specific variables including, but not limited to, the tightness of specific ligaments and tendons. Flexibility exercises enhance the ability of a joint to move through its full range of motion.
Frailty	Frailer older adults are those who are identified as being frail or have very low physical or cognitive function, perhaps because of chronic disease such as arthritis, dementia, or advanced old age itself.
Functional training	Exercises that can be embedded into everyday tasks to improve lower-body strength, balance, and motor performance. Examples include tandem and one-leg stands, squatting, chair stands, toe raises, and stepping over obstacles.
Interactive activities / play	Activities with a parent or caregiver that do not involve screens as a mode of entertainment. These can include reading, singing, storytelling, colouring, block building, cutting out, puzzles and games.<br><br>See "Play".<br>Interactive play is play with a parent or caregiver where the child and adult/older child interact and engage in play for both cognitive and motor learning.
Light-intensity physical activity	Light-intensity physical activities between 1.5 and 3 METS, i.e. activities with energy cost less than 3 times the energy expenditure at rest for that person.<br><br>This can include slow walking, bathing, or other incidental activities that do not result in a substantial increase in heart rate or breathing rate.

GLOSSARY OF TERMS

Term	Definition
Major muscle groups	Major muscle groups include the legs, back, abdomen, chest, shoulders and arms.
Metabolic equivalent of task (MET)	The metabolic equivalent of task, or simply metabolic equivalent, is a physiological measure expressing the energy cost (or calories) of physical activities. One MET is the energy equivalent expended by an individual while seated at rest.
Moderate-intensity physical activity	On an absolute scale, moderate-intensity refers to the physical activity that is performed between 3 and less than 6 times the intensity of rest. On a scale relative to an individual's personal capacity, moderate-intensity physical activity is usually 5 or 6 on a scale of 0-10.
Motor Development - Fundamental Movement Skills (FMS)	Development of a child's musculoskeletal system and acquisition of gross motor skills (sometimes referred to as fundamental movement skills FMS), and fine motor skills, including object control.
Muscle-strengthening activity	Physical activity and exercise that increase skeletal muscle strength, power, endurance, and mass (e.g. strength training, resistance training, or muscular strength and endurance exercises).
Multi-component physical activity	Multi-component physical activity is important to improve physical function and decrease the risk of falls or injury from a fall. These activities can be done at home or in a structured group setting. Many studied interventions combine all types of exercise (aerobic, muscle strengthening, and balance training) into a session, and this has been shown to be effective. An example of a multi-component physical activity programme could include walking) aerobic activity), lifting weights (muscle strengthening), and incorporates balance training. Examples of balance training can include walking backwards or sideways or standing on one foot while doing an upper body muscle-strengthening activity, such as bicep curls. Dancing also combines aerobic and balance components.
Non-Communicable Diseases (NCDs)	A disease that is not transmissible directly from one person to another
Physical activity	Any bodily movement produced by skeletal muscles that requires energy expenditure.
Physical inactivity	An insufficient physical activity level to meet present physical activity recommendations.

GLOSSARY OF TERMS

Term	Definition
Play	Play is defined as being for its own sake (without a specific goal), voluntary, enjoyed by participants and imaginative. It can be solitary or social, and with or without objects. Young children acquire and consolidate developmental skills through playful interactions with people and objects.
Postpartum	Postpartum refers to a period after the end of pregnancy. The postpartum period is commonly defined as up to six weeks following the end of pregnancy, with the late postpartum period from six weeks up to one year after the end of pregnancy. For these Guidelines postpartum includes up to one year post delivery.
Psychosocial health	Include mental, emotional, and social dimensions of health.
Recreational screen time	Time spent watching screens (television (TV), computer, mobile devices) for purposes other than those related to education/study or work.
Sedentary behaviour	Any waking behaviour characterized by an energy expenditure of 1.5 METS or lower while sitting, reclining, or lying. Most desk-based office work, driving a car, and watching television are examples of sedentary behaviours; these can also apply to those unable to stand, such as wheelchair users. The guidelines operationalize the definition of sedentary behaviour to include self-reported low movement sitting (leisure time, occupational, and total), television (TV viewing or screen time, and low levels of movement measured by devices that assess movement or posture).
Sport	Sport covers a range of activities performed within a set of rules and undertaken as part of leisure or competition. Sporting activities involve physical activity carried out by teams or individuals and may be supported by an institutional framework, such as a sporting agency.
Vigorous-intensity physical activity	On an absolute scale, vigorous-intensity refers to physical activity that is performed at 6.0 or more METS. On a scale relative to an individual's personal capacity, vigorous-intensity physical activity is usually 7 or 8 on a scale of 0-10. Every minute of vigorous-intensity activity can generally be considered as two minutes worth of moderate-intensity activity.
Tummy time	Time an infant spends lying on their front (in prone position) while awake with unrestricted movement of limbs.

Sources: WHO Guidelines on Physical Activity, Sedentary Behaviour and Sleep 2019; WHO Guidelines on Physical Activity and Sedentary Behaviour 2020; UK Chief Medical Officers' (CMO) Physical Activity Guidelines 2019

APPENDIX

Section	Page	Evidence Point	References
5	9	Coronary Heart Disease	Sattelmair, J., Pertman, J., Ding, E. L., Kohl III, H. W., Haskell, W., & Lee, I.-M. (2011). Dose response between physical activity and risk of coronary heart disease: A meta-analysis. *Circulation, 124*(7), 789–795
5	9	Hypertension	Diaz, K. M., & Shimbo, D. (2013). Physical activity and the prevention of hypertension. *Current Hypertension Reports, 15*(6), 659–668.
5	9	Stroke	Lee, J. (2020). Associations between Physical Activity and Liver Cancer Risks and Mortality: A Systematic Review and Meta-Analysis. *International Journal of Environmental Research and Public Health, 17*(23), 8943. https://doi.org/10.3390/ijerph17238943
5	9	Type 2 Diabetes	Smith, A. D., Crippa, A., Woodcock, J., & Brage, S. (2016). Physical activity and incident type 2 diabetes mellitus: A systematic review and dose–response meta-analysis of prospective cohort studies. *Diabetologia, 59*(12), 2527–2545.
5	9	Colon Cancer	Wolin, K. Y., Yan, Y., Colditz, G. A., & Lee, I. (2009). Physical activity and colon cancer prevention: A meta-analysis. *British Journal of Cancer, 100*(4), 611–616.
5	9	Uterine Cancer	Schmid, D., Behrens, G., Keimling, M., Jochem, C., Ricci, C., & Leitzmann, M. (2015). A systematic review and meta-analysis of physical activity and endometrial cancer risk. *European Journal of Epidemiology, 30*(5), 397–412.
5	10	Cognitive Function	Donnelly, J. E., Hillman, C. H., Castelli, D., Etnier, J. L., Lee, S., Tomporowski, P., Lambourne, K., & Szabo-Reed, A. N. (2016). Physical Activity, Fitness, Cognitive Function, and Academic Achievement in Children: A Systematic Review. *Medicine and Science in Sports and Exercise, 48*(6), 1197–1222. https://doi.org/10.1249/MSS.0000000000000901
5	10	Emotional Health	Maher, J. P., Pincus, A. L., Ram, N., & Conroy, D. E. (2015). Daily Physical Activity and Life Satisfaction Across Adulthood. *Developmental Psychology, 51*(10), 1407–1419. https://doi.org/10.1037/dev0000037
5	10	Anxiety	Chekroud, S. R., Gueorguieva, R., Zheutlin, A. B., Paulus, M., Krumholz, H. M., Krystal, J. H., & Chekroud, A. M. (2018). Association between physical exercise and mental health in 1· 2 million individuals in the USA between 2011 and 2015: A cross-sectional study. *The Lancet Psychiatry, 5*(9), 739–746.

APPENDIX

Section	Page	Evidence Point	References
5	10	Depression	Phillips, C. (2017). Brain-Derived Neurotrophic Factor, Depression, and Physical Activity: Making the Neuroplastic Connection. *Neural Plasticity, 2017,* 7260130–17. https://doi.org/10.1155/2017/7260130
5	10	Alzheimers Disease and Dementia	Jia, R.-X., Liang, J.-H., Xu, Y., & Wang, Y.-Q. (2019). Effects of physical activity and exercise on the cognitive function of patients with Alzheimer disease: A meta-analysis. *BMC Geriatrics, 19*(1), 181–181. https://doi.org/10.1186/s12877-019-1175-2
5	11	Social Skills and Interaction	Hotting, K., & Roder, B. (2013). Beneficial effects of physical exercise on neuroplasticity and cognition. *Neuroscience and Biobehavioral Reviews, 37*(9 Pt B), 2243–2257. https://doi.org/10.1016/j.neubiorev.2013.04.005
5	11	Cognitive Functioning	Biddle, S. J. H., Ciaccioni, S., Thomas, G., & Vergeer, I. (2019). Physical activity and mental health in children and adolescents: An updated review of reviews and an analysis of causality. *Psychology of Sport and Exercise, 42,* 146–155. https://doi.org/10.1016/j.psychsport.2018.08.011
5	11	Concentration	de Greeff, J. W., Bosker, R. J., Oosterlaan, J., Visscher, C., & Hartman, E. (2018). Effects of physical activity on executive functions, attention and academic performance in preadolescent children: A meta-analysis. *Journal of Science and Medicine in Sport, 21*(5), 501–507.
5	11	Memory and Executive Function	US Department of Health and Human Services. (2018). *Physical activity guidelines advisory committee. 2018 physical activity guidelines advisory committee scientific report.*
5	11	Cognitive Function	*Global Health Observatory data repository.* (2021). World Health Organization. http://www.who.int/gho/en/
7	14	Obesity	*Global Health Observatory data repository.* (2021). World Health Organization. http://www.who.int/gho/en/
7	14	Activity Levels	*Global Health Observatory data repository.* (2021). World Health Organization. http://www.who.int/gho/en/
7	15	Obesity	*National Population Health Survey.* (2020). Health Promotion Board. https://www.hpb.gov.sg/community/national-population-health-survey
7	15	Diabetes	*National Population Health Survey.* (2020). Health Promotion Board. https://www.hpb.gov.sg/community/national-population-health-survey

APPENDIX

Section	Page	Evidence Point	References
7	15	Activity Levels	*National Population Health Survey.* (2020). Health Promotion Board. https://www.hpb.gov.sg/community/national-population-health-survey
8	23	Childhood Obesity	Nishtar, S., Gluckman, P., & Armstrong, T. (2016). Ending childhood obesity: A time for action. *The Lancet (British Edition), 387*(10021), 825–827. https://doi.org/10.1016/S0140-6736(16)00140-9
5	23	Physical Activity Habits	Hallal, P. C., Dr, Andersen, L. B., Prof, Bull, F. C., Prof, Guthold, R., PhD, Haskell, W., Prof, & Ekelund, U., Prof. (2012). Global physical activity levels: Surveillance progress, pitfalls, and prospects. *The Lancet (British Edition), 380*(9838), 247–257. https://doi.org/10.1016/S0140-6736(12)60646-1
8	23	Cognitive Development	Chaput, J.-P., Gray, C. E., Poitras, V. J., Carson, V., Gruber, R., Olds, T., Weiss, S. K., Connor Gorber, S., Kho, M. E., Sampson, M., Belanger, K., Eryuzlu, S., Callender, L., & Tremblay, M. S. (2016). Systematic review of the relationships between sleep duration and health indicators in school-aged children and youth. *Applied Physiology, Nutrition, and Metabolism, 41*(6 Suppl 3), S266–S282. https://doi.org/10.1139/apnm-2015-0627
8	23	Bone Mineral Density and Muscular Development	Carson, V., Hunter, S., Kuzik, N., Gray, C. E., Poitras, V. J., Chaput, J.-P., Saunders, T. J., Katzmarzyk, P. T., Okely, A. D., Connor Gorber, S., Kho, M. E., Sampson, M., Lee, H., & Tremblay, M. S. (2016). Systematic review of sedentary behaviour and health indicators in school-aged children and youth: An update. *Applied Physiology, Nutrition, and Metabolism, 41*(6 Suppl 3), S240–S265. https://doi.org/10.1139/apnm-2015-0630
8	23	Fundamental Movement Skills	Barnett, L. M., Stodden, D., Cohen, K. E., Smith, J. J., Lubans, D. R., Lenoir, M., Iivonen, S., Miller, A. D., Laukkanen, A., & Dudley, D. (2016). Fundamental movement skills: An important focus. *Journal of Teaching in Physical Education, 35*(3), 219–225.
8	24	Sedentary Behaviour	Okely, A. D., Ghersi, D., Hesketh, K. D., Santos, R., Loughran, S. P., Cliff, D. P., Shilton, T., Grant, D., Jones, R. A., Stanley, R. M., Sherring, J., Hinkley, T., Trost, S. G., McHugh, C., Eckermann, S., Thorpe, K., Waters, K., Olds, T. S., Mackey, T., … Tremblay, M. S. (2017). A collaborative approach to adopting/adapting guidelines—The Australian 24-Hour Movement Guidelines for the early years (Birth to 5 years): An integration of physical activity, sedentary behavior, and sleep. *BMC Public Health, 17*(Suppl 5), 869–869. https://doi.org/10.1186/s12889-017-4867-6

APPENDIX

Section	Page	Evidence Point	References
8	24	Social Behaviour	Carson, V., Hunter, S., Kuzik, N., Gray, C. E., Poitras, V. J., Chaput, J.-P., Saunders, T. J., Katzmarzyk, P. T., Okely, A. D., Connor Gorber, S., Kho, M. E., Sampson, M., Lee, H., & Tremblay, M. S. (2016). Systematic review of sedentary behaviour and health indicators in school-aged children and youth: An update. *Applied Physiology, Nutrition, and Metabolism, 41*(6 Suppl 3), S240–S265. https://doi.org/10.1139/apnm-2015-0630
8	24	Obesity	Fang, K., Mu, M., Liu, K., & He, Y. (2019). Screen time and childhood overweight/obesity: A systematic review and meta-analysis. *Child : Care, Health & Development, 45*(5), 744–753. https://doi.org/10.1111/cch.12701
8	24	Mental Health	Hoare, E., Milton, K., Foster, C., & Allender, S. (2016). The associations between sedentary behaviour and mental health among adolescents: A systematic review. *The International Journal of Behavioral Nutrition and Physical Activity, 13*(1), 108–108. https://doi.org/10.1186/s12966-016-0432-4
8	24	Fitness	Poitras, V. J., Gray, C. E., Borghese, M. M., Carson, V., Chaput, J.-P., Janssen, I., Katzmarzyk, P. T., Pate, R. R., Connor Gorber, S., Kho, M. E., Sampson, M., & Tremblay, M. S. (2016). Systematic review of the relationships between objectively measured physical activity and health indicators in school-aged children and youth. *Applied Physiology, Nutrition, and Metabolism, 41*(6 Suppl 3), S197–S239. https://doi.org/10.1139/apnm-2015-0663
8	24	Mental Health	Bursnall, P. (2014). The relationship between physical activity and depressive symptoms in adolescents: A systematic review. *Worldviews on Evidence-Based Nursing, 11*(6), 376–382.
8	24	Cognitive Function	Poitras, V. J., Gray, C. E., Borghese, M. M., Carson, V., Chaput, J.-P., Janssen, I., Katzmarzyk, P. T., Pate, R. R., Connor Gorber, S., Kho, M. E., Sampson, M., & Tremblay, M. S. (2016). Systematic review of the relationships between objectively measured physical activity and health indicators in school-aged children and youth. *Applied Physiology, Nutrition, and Metabolism, 41*(6 Suppl 3), S197–S239. https://doi.org/10.1139/apnm-2015-0663
8	24	Cardiometabolic Health	US Department of Health and Human Services. (2018). *Physical activity guidelines advisory committee. 2018 physical activity guidelines advisory committee scientific report.*
8	24	Growth and Development	Faigenbaum, A. D., Kraemer, W. J., Blimkie, C. J., Jeffreys, I., Micheli, L. J., Nitka, M., & Rowland, T. W. (2009). Youth resistance training: Updated position statement paper from the national strength and conditioning association. *The Journal of Strength & Conditioning Research, 23,* S60–S79.

APPENDIX

Section	Page	Evidence Point	References
8	24	Cardiometabolic Health	US Department of Health and Human Services. (2018). *Physical activity guidelines advisory committee. 2018 physical activity guidelines advisory committee scientific report.*
8	24	Growth and Development	Faigenbaum, A. D., Kraemer, W. J., Blimkie, C. J., Jeffreys, I., Micheli, L. J., Nitka, M., & Rowland, T. W. (2009). Youth resistance training: Updated position statement paper from the national strength and conditioning association. *The Journal of Strength & Conditioning Research, 23,* S60–S79.
8	24	Bone Development	US Department of Health and Human Services. (2018). *Physical activity guidelines advisory committee. 2018 physical activity guidelines advisory committee scientific report.*
8	24	Fundamental Movement Skills	Barnett, L. M., Stodden, D., Cohen, K. E., Smith, J. J., Lubans, D. R., Lenoir, M., Iivonen, S., Miller, A. D., Laukkanen, A., & Dudley, D. (2016). Fundamental movement skills: An important focus. *Journal of Teaching in Physical Education, 35*(3), 219–225.
8	24	Cardiorespiratory Fitness	Cao, M., Quan, M., & Zhuang, J. (2019). Effect of high-intensity interval training versus moderate-intensity continuous training on cardiorespiratory fitness in children and adolescents: A meta-analysis. *International Journal of Environmental Research and Public Health, 16*(9), 1533.
8	28	Cardiovascular Disease	Bailey, D. P., Hewson, D. J., Champion, R. B., & Sayegh, S. M. (2019). Sitting Time and Risk of Cardiovascular Disease and Diabetes: A Systematic Review and Meta-Analysis. *American Journal of Preventive Medicine, 57*(3), 408–416. https://doi.org/10.1016/j.amepre.2019.04.015
8	28	Sleep	US Department of Health and Human Services. (2018). *Physical activity guidelines advisory committee. 2018 physical activity guidelines advisory committee scientific report.*
8	28	Type-2 Diabetes	Patterson, R., McNamara, E., Tainio, M., de Sá, T. H., Smith, A. D., Sharp, S. J., Edwards, P., Woodcock, J., Brage, S., & Wijndaele, K. (2018). Sedentary behaviour and risk of all-cause, cardiovascular and cancer mortality, and incident type 2 diabetes: A systematic review and dose response meta-analysis. *European Journal of Epidemiology, 33*(9), 811–829.
8	28	Mental Health	Schuch, F. B., Vancampfort, D., Rosenbaum, S., Richards, J., Ward, P. B., Veronese, N., Solmi, M., Cadore, E. L., & Stubbs, B. (2016). Exercise for depression in older adults: A meta-analysis of randomized controlled trials adjusting for publication bias. *Revista Brasileira de Psiquiatria, 38*(3), 247–254. https://doi.org/10.1590/1516-4446-2016-1915

APPENDIX

Section	Page	Evidence Point	References
8	28	Mental Health	Schuch, F. B., Vancampfort, D., Firth, J., Rosenbaum, S., Ward, P. B., Silva, E. S., Hallgren, M., Ponce De Leon, A., Dunn, A. L., & Deslandes, A. C. (2018). Physical activity and incident depression: A meta-analysis of prospective cohort studies. *American Journal of Psychiatry, 175*(7), 631–648.
8	28	Hypertension	US Department of Health and Human Services. (2018). *Physical activity guidelines advisory committee. 2018 physical activity guidelines advisory committee scientific report.*
8	28	Liver Cancer	Lee, J. (2020). Associations between Physical Activity and Liver Cancer Risks and Mortality: A Systematic Review and Meta-Analysis. *International Journal of Environmental Research and Public Health, 17*(23), 8943. https://doi.org/10.3390/ijerph17238943
8	28	Mental Health	Schuch, F. B., Vancampfort, D., Rosenbaum, S., Richards, J., Ward, P. B., Veronese, N., Solmi, M., Cadore, E. L., & Stubbs, B. (2016). Exercise for depression in older adults: A meta-analysis of randomized controlled trials adjusting for publication bias. *Revista Brasileira de Psiquiatria, 38*(3), 247–254. https://doi.org/10.1590/1516-4446-2016-1915
8	28	Weight Management	Westcott, W. L. (2012). Resistance training is medicine: Effects of strength training on health. *Current Sports Medicine Reports, 11*(4), 209–216.
8	28	Bone Health	Westcott, W. L. (2012). Resistance training is medicine: Effects of strength training on health. *Current Sports Medicine Reports, 11*(4), 209–216.
8	28	Mental Health	Gordon, B. R., McDowell, C. P., Hallgren, M., Meyer, J. D., Lyons, M., & Herring, M. P. (2018a). Association of efficacy of resistance exercise training with depressive symptoms: Meta-analysis and meta-regression analysis of randomized clinical trials. *JAMA Psychiatry, 75*(6), 566–576.
8	28	Cognitive Function	Rathore, A., & Lom, B. (2017). The effects of chronic and acute physical activity on working memory performance in healthy participants: A systematic review with meta-analysis of randomized controlled trials. *Systematic Reviews, 6*(1), 124–124. https://doi.org/10.1186/s13643-017-0514-7
8	34	Gestational Weight Gain	Du, M., Ouyang, Y., Nie, X., Huang, Y., & Redding, S. R. (2019). Effects of physical exercise during pregnancy on maternal and infant outcomes in overweight and obese pregnant women: A meta-analysis. *Birth, 46*(2), 211–221.
8	34	Gestational Diabetes	Du, M., Ouyang, Y., Nie, X., Huang, Y., & Redding, S. R. (2019). Effects of physical exercise during pregnancy on maternal and infant outcomes in overweight and obese pregnant women: A meta-analysis. *Birth, 46*(2), 211–221.

APPENDIX

Section	Page	Evidence Point	References
8	34	Premature Birth	Beetham, K. S., Giles, C., Noetel, M., Clifton, V., Jones, J. C., & Naughton, G. (2019). The effects of vigorous intensity exercise in the third trimester of pregnancy: A systematic review and meta-analysis. *BMC Pregnancy and Childbirth, 19*(1), 281–281. https://doi.org/10.1186/s12884-019-2441-1
8	34	Postpartum Depression	Davenport, M. H., McCurdy, A. P., Mottola, M. F., Skow, R. J., Meah, V. L., Poitras, V. J., Jaramillo Garcia, A., Gray, C. E., Barrowman, N., Riske, L., Sobierajski, F., James, M., Nagpal, T., Marchand, A.-A., Nuspl, M., Slater, L. G., Barakat, R., Adamo, K. B., Davies, G. A., & Ruchat, S.-M. (2018). Impact of prenatal exercise on both prenatal and postnatal anxiety and depressive symptoms: A systematic review and meta-analysis. *British Journal of Sports Medicine, 52*(21), 1376–1385. https://doi.org/10.1136/bjsports-2018-099697
8	34	Postpartum Weight Gain	Davenport, M. H., McCurdy, A. P., Mottola, M. F., Skow, R. J., Meah, V. L., Poitras, V. J., Jaramillo Garcia, A., Gray, C. E., Barrowman, N., Riske, L., Sobierajski, F., James, M., Nagpal, T., Marchand, A.-A., Nuspl, M., Slater, L. G., Barakat, R., Adamo, K. B., Davies, G. A., & Ruchat, S.-M. (2018). Impact of prenatal exercise on both prenatal and postnatal anxiety and depressive symptoms: A systematic review and meta-analysis. *British Journal of Sports Medicine, 52*(21), 1376–1385. https://doi.org/10.1136/bjsports-2018-099697
8	34	Recovery	Davenport, M. H., Ruchat, S.-M., Sobierajski, F., Poitras, V. J., Gray, C. E., Yoo, C., Skow, R. J., Jaramillo Garcia, A., Barrowman, N., Meah, V. L., Nagpal, T. S., Riske, L., James, M., Nuspl, M., Weeks, A., Marchand, A.-A., Slater, L. G., Adamo, K. B., Davies, G. A., … Mottola, M. F. (2019). Impact of prenatal exercise on maternal harms, labour and delivery outcomes: A systematic review and meta-analysis. *British Journal of Sports Medicine, 53*(2), 99–107. https://doi.org/10.1136/bjsports-2018-099821
8	38	Physical Function	US Department of Health and Human Services. (2018). *Physical activity guidelines advisory committee. 2018 physical activity guidelines advisory committee scientific report.*
9	38	Physical Function	Alphonsus, K. B., Su, Y., & D'Arcy, C. (2019). The effect of exercise, yoga and physiotherapy on the quality of life of people with multiple sclerosis: Systematic review and meta-analysis. *Complementary Therapies in Medicine, 43,* 188–195. https://doi.org/10.1016/j.ctim.2019.02.010
8	38	Mental Health	Krogh, J., Hjorthøj, C., Speyer, H., Gluud, C., & Nordentoft, M. (2017). Exercise for patients with major depression: A systematic review with meta-analysis and trial sequential analysis. *BMJ Open, 7*(9), e014820–e014820. https://doi.org/10.1136/bmjopen-2016-014820

APPENDIX

Section	Page	Evidence Point	References
8	38	Cognitive Function	Stuckenschneider, T., Askew, C. D., Meneses, A. L., Baake, R., Weber, J., & Schneider, S. (2019). The effect of different exercise modes on domain-specific cognitive function in patients suffering from Parkinson's disease: A systematic review of randomized controlled trials. *Journal of Parkinson's Disease, 9*(1), 73–95.
8	42	Joint Complications	US Department of Health and Human Services. (2018). *Physical activity guidelines advisory committee. 2018 physical activity guidelines advisory committee scientific report.*
8	42	Functional Performance	US Department of Health and Human Services. (2018). *Physical activity guidelines advisory committee. 2018 physical activity guidelines advisory committee scientific report.*
8	42	Overweight and Obesity	*The National Guidelines on Physical Activity for Ireland.* (2009). Department of Health and Children, Health Service Executive. https://www.ncn.ie/images/GuidelinesPhysicalActivity.pdf
8	42	Muscle Function, Balance, and Flexibility	Smith, B., Kirby, N., Skinner, B., Wightman, L., Lucas, R., & Foster, C. (2018). *Physical activity for general health benefits in disabled adults: Summary of a rapid evidence review for the UK Chief Medical Officers' update of the physical activity guidelines.*
8	43	All-Cause Mortality	Hupin, D., Roche, F., Gremeaux, V., Chatard, J.-C., Oriol, M., Gaspoz, J.-M., Barthélémy, J.-C., & Edouard, P. (2015). Even a low-dose of moderate-to-vigorous physical activity reduces mortality by 22% in adults aged ≥60 years: A systematic review and meta-analysis. *British Journal of Sports Medicine, 49*(19), 1262–1267. https://doi.org/10.1136/bjsports-2014-094306
9	43	Cardiovascular Disease	Ekelund, U., Prof, Steene-Johannessen, J., PhD, Brown, W. J., Prof, Fagerland, M. W., PhD, Owen, N., Prof, Powell, K. E., MD, Bauman, A., Prof, & Lee, I.-M., Prof. (2016). Does physical activity attenuate, or even eliminate, the detrimental association of sitting time with mortality? A harmonised meta-analysis of data from more than 1 million men and women. *The Lancet (British Edition), 388*(10051), 1302–1310. https://doi.org/10.1016/S0140-6736(16)30370-1
8	43	Physical Function	Keevil, V. L., Luben, R., Hayat, S., Sayer, A. A., Wareham, N. J., & Khaw, K.-T. (2018). Physical capability predicts mortality in late mid-life as well as in old age: Findings from a large British cohort study. *Archives of Gerontology and Geriatrics, 74*, 77–82. https://doi.org/10.1016/j.archger.2017.10.001

APPENDIX

Section	Page	Evidence Point	References
8	43	Physical Function	Jadczak, A. D., Makwana, N., Luscombe-Marsh, N., Visvanathan, R., & Schultz, T. J. (2018). Effectiveness of exercise interventions on physical function in community-dwelling frail older people: An umbrella review of systematic reviews. *JBI Evidence Synthesis, 16*(3), 752–775.
8	43	Bone Health	Onambele-Pearson, G., Wullems, J., Doody, C., Ryan, D., Morse, C., & Degens, H. (2019). Influence of Habitual Physical Behavior—Sleeping, Sedentarism, Physical Activity—On Bone Health in Community-Dwelling Older People. *Frontiers in Physiology, 10,* 408–408. https://doi.org/10.3389/fphys.2019.00408
8	43	Cardiovascular Disease	Blond, K., Brinkløv, C. F., Ried-Larsen, M., Crippa, A., & Grøntved, A. (2020). Association of high amounts of physical activity with mortality risk: A systematic review and meta-analysis. *British Journal of Sports Medicine, 54*(20), 1195–1201. https://doi.org/10.1136/bjsports-2018-100393
8	43	Cognitive Function	Catalan-Matamoros, D., Gomez-Conesa, A., Stubbs, B., & Vancampfort, D. (2016). Exercise improves depressive symptoms in older adults: An umbrella review of systematic reviews and meta-analyses. *Psychiatry Research, 244,* 202–209. https://doi.org/10.1016/j.psychres.2016.07.028
8	43	Life Satisfaction	Loprinzi, P. D., Edwards, M. K., Crush, E., Ikuta, T., & Del Arco, A. (2018). Dose–Response Association Between Physical Activity and Cognitive Function in a National Sample of Older Adults. *American Journal of Health Promotion, 32*(3), 554–560. https://doi.org/10.1177/0890117116689732
8	43	Falls	Sherrington, C., Fairhall, N., Wallbank, G., Tiedemann, A., Michaleff, Z. A., Howard, K., Clemson, L., Hopewell, S., & Lamb, S. (2020). Exercise for preventing falls in older people living in the community: An abridged Cochrane systematic review. *British Journal of Sports Medicine, 54*(15), 885–891. https://doi.org/10.1136/bjsports-2019-101512
8	43	Mental Health	Gordon, B. R., McDowell, C. P., Hallgren, M., Meyer, J. D., Lyons, M., & Herring, M. P. (2018b). The Effects of Resistance Exercise Training on Depressive Symptoms: A Meta-Analysis of Randomized Controlled Trials 103 Board #1 May 30 9 30 AM - 11 30 AM. *Medicine and Science in Sports and Exercise, 50*(5S), 5. https://doi.org/10.1249/01.mss.0000535100.03519.6a
8	43	Bone Health	Brown, W. J., Bauman, A. E., Bull, F., & Burton, N. W. (2013). *Development of Evidence-based Physical Activity Recommendations for Adults (18-64 years). Report prepared for the Australian Government Department of Health, August 2012.*

Get Active Questionnaire

CANADIAN SOCIETY FOR EXERCISE PHYSIOLOGY –
PHYSICAL ACTIVITY TRAINING FOR HEALTH (CSEP-PATH®)

Physical activity improves your physical and mental health. Even small amounts of physical activity are good, and more is better.

For almost everyone, the benefits of physical activity far outweigh any risks. For some individuals, specific advice from a Qualified Exercise Professional (QEP – has post-secondary education in exercise sciences and an advanced certification in the area – see csep.ca/certifications) or health care provider is advisable. **This questionnaire is intended for all ages – to help move you along the path to becoming more physically active.**

☐ **I am completing this questionnaire for myself.**

☐ **I am completing this questionnaire for my child/dependent as parent/guardian.**

PREPARE TO BECOME MORE ACTIVE

The following questions will help to ensure that you have a safe physical activity experience. Please answer YES or NO to each question before you become more physically active. If you are unsure about any question, answer YES.

YES	NO	
		**1** Have you experienced ANY of the following (A to F) **within the past six months?**
○	○	**A** A diagnosis of/treatment for heart disease or stroke, or pain/discomfort/pressure in your chest during activities of daily living or during physical activity?
○	○	**B** A diagnosis of/treatment for high blood pressure (BP), or a resting BP of 160/90 mmHg or higher?
○	○	**C** Dizziness or lightheadedness during physical activity?
○	○	**D** Shortness of breath at rest?
○	○	**E** Loss of consciousness/fainting for any reason?
○	○	**F** Concussion?
○	○	**2** Do you currently have pain or swelling in any part of your body (such as from an injury, acute flare-up of arthritis, or back pain) that affects your ability to be physically active?
○	○	**3** Has a health care provider told you that you should avoid or modify certain types of physical activity?
○	○	**4** Do you have any other medical or physical condition (such as diabetes, cancer, osteoporosis, asthma, spinal cord injury) that may affect your ability to be physically active?

NO to all questions: go to Page 2 – ASSESS YOUR CURRENT PHYSICAL ACTIVITY ➤

YES to any question: go to Reference Document – ADVICE ON WHAT TO DO IF YOU HAVE A YES RESPONSE ➤➤

© Canadian Society for Exercise Physiology, 2017. All rights reserved.

Get Active Questionnaire – Reference Document
ADVICE ON WHAT TO DO IF YOU HAVE A **YES** RESPONSE

CSEP | SCPE
THE GOLD STANDARD IN EXERCISE SCIENCE AND PERSONAL TRAINING

Use this reference document if you answered **YES** to any question and you have not consulted a health care provider or Qualified Exercise Professional (QEP) about becoming more physically active.

**1 Have you experienced ANY of the following (A to F) within the past six months?**	
**A** A diagnosis of/treatment for heart disease or stroke, or pain/ discomfort/pressure in your chest during activities of daily living or during physical activity? ☐ YES	Physical activity is likely to be beneficial. If you have been treated for heart disease but have not completed a cardiac rehabilitation program within the past 6 months, consult a doctor – a supervised cardiac rehabilitation program is strongly recommended. If you are resuming physical activity after more than 6 months of inactivity, begin slowly with light- to moderate-intensity physical activity. If you have pain/discomfort/pressure in your chest and it is new for you, talk to a doctor. Describe the symptom and what activities bring it on.
**B** A diagnosis of/treatment for high blood pressure (BP), or a resting BP of 160/90 mmHg or higher? ☐ YES	Physical activity is likely to be beneficial if you have been diagnosed and treated for high blood pressure (BP). If you are unsure of your resting BP, consult a health care provider or a Qualified Exercise Professional (QEP) to have it measured. If you are taking BP medication and your BP is under good control, regular physical activity is recommended as it may help to lower your BP. Your doctor should be aware of your physical activity level so your medication needs can be monitored. If your BP is 160/90 or higher, you should receive medical clearance and consult a QEP about safe and appropriate physical activity.
**C** Dizziness or lightheadedness during physical activity ☐ YES	There are several possible reasons for feeling this way and many are not worrisome. Before becoming more active, consult a health care provider to identify reasons and minimize risk. Until then, refrain from increasing the intensity of your physical activity.
**D** Shortness of breath at rest ☐ YES	If you have asthma and this is relieved with medication, light to moderate physical activity is safe. If your shortness of breath is not relieved with medication, consult a doctor.
**E** Loss of consciousness/ fainting for any reason ☐ YES	Before becoming more active, consult a doctor to identify reasons and minimize risk. Once you are medically cleared, consult a Qualified Exercise Professional (QEP) about types of physical activity suitable for your condition.
**F** Concussion ☐ YES	A concussion is an injury to the brain that requires time to recover. Increasing physical activity while still experiencing symptoms may worsen your symptoms, lengthen your recovery, and increase your risk for another concussion. A health care provider will let you know when you can start becoming more physically active, and a Qualified Exercise Professional (QEP) can help get you started.

After reading the ADVICE for your YES response, go to Page 2 of the *Get Active Questionnaire* – ASSESS YOUR CURRENT PHYSICAL ACTIVITY

© Canadian Society for Exercise Physiology, 2017. All rights reserved.

CSEP | SCPE
THE GOLD STANDARD IN EXERCISE SCIENCE AND PERSONAL TRAINING

Get Active Questionnaire – Reference Document

ADVICE ON WHAT TO DO IF YOU HAVE A **YES** RESPONSE

Use this reference document if you answered **YES** to any question and you have not consulted a health care provider or Qualified Exercise Professional (QEP) about becoming more physically active.

**1 Have you experienced ANY of the following (A to F) within the past six months?**	
**A** A diagnosis of/treatment for heart disease or stroke, or pain/ discomfort/pressure in your chest during activities of daily living or during physical activity? ☐ **YES**	Physical activity is likely to be beneficial. If you have been treated for heart disease but have not completed a cardiac rehabilitation program within the past 6 months, consult a doctor – a supervised cardiac rehabilitation program is strongly recommended. If you are resuming physical activity after more than 6 months of inactivity, begin slowly with light- to moderate-intensity physical activity. If you have pain/discomfort/pressure in your chest and it is new for you, talk to a doctor. Describe the symptom and what activities bring it on.
**B** A diagnosis of/treatment for high blood pressure (BP), or a resting BP of 160/90 mmHg or higher? ☐ **YES**	Physical activity is likely to be beneficial if you have been diagnosed and treated for high blood pressure (BP). If you are unsure of your resting BP, consult a health care provider or a Qualified Exercise Professional (QEP) to have it measured. If you are taking BP medication and your BP is under good control, regular physical activity is recommended as it may help to lower your BP. Your doctor should be aware of your physical activity level so your medication needs can be monitored. If your BP is 160/90 or higher, you should receive medical clearance and consult a QEP about safe and appropriate physical activity.
**C** Dizziness or lightheadedness during physical activity ☐ **YES**	There are several possible reasons for feeling this way and many are not worrisome. Before becoming more active, consult a health care provider to identify reasons and minimize risk. Until then, refrain from increasing the intensity of your physical activity.
**D** Shortness of breath at rest ☐ **YES**	If you have asthma and this is relieved with medication, light to moderate physical activity is safe. If your shortness of breath is not relieved with medication, consult a doctor.
**E** Loss of consciousness/ fainting for any reason ☐ **YES**	Before becoming more active, consult a doctor to identify reasons and minimize risk. Once you are medically cleared, consult a Qualified Exercise Professional (QEP) about types of physical activity suitable for your condition.
**F** Concussion ☐ **YES**	A concussion is an injury to the brain that requires time to recover. Increasing physical activity while still experiencing symptoms may worsen your symptoms, lengthen your recovery, and increase your risk for another concussion. A health care provider will let you know when you can start becoming more physically active, and a Qualified Exercise Professional (QEP) can help get you started.

After reading the ADVICE for your YES response, go to Page 2 of the *Get Active Questionnaire* – ASSESS YOUR CURRENT PHYSICAL ACTIVITY

© Canadian Society for Exercise Physiology, 2017. All rights reserved.

CSEP | SCPE
THE GOLD STANDARD IN EXERCISE SCIENCE AND PERSONAL TRAINING

Get Active Questionnaire – Reference Document
ADVICE ON WHAT TO DO IF YOU HAVE A **YES** RESPONSE

Use this reference document if you answered **YES** to any question and you have not consulted a health care provider or Qualified Exercise Professional (QEP) about becoming more physically active.

2 Do you currently have pain or swelling in any part of your body (such as from an injury, acute flare-up of arthritis, or back pain) that affects your ability to be physically active? ☐ **YES**

If this swelling or pain is new, consult a health care provider. Otherwise, keep joints healthy and reduce pain by moving your joints slowly and gently through the entire pain-free range of motion. If you have hip, knee or ankle pain, choose low-impact activities such as swimming or cycling. As the pain subsides, gradually resume your normal physical activities starting at a level lower than before the flare-up. Consult a Qualified Exercise Professional (QEP) in follow-up to help you become more active and prevent or minimize future pain.

3 Has a health care provider told you that you should avoid or modify certain types of physical activity? ☐ **YES**

Listen to the advice of your health care provider. A Qualified Exercise Professional (QEP) will ask you about any considerations and provide specific advice for physical activity that is safe and that takes your lifestyle and health care provider's advice into account.

4 Do you have any other medical or physical condition (such as diabetes, cancer, osteoporosis, asthma, spinal cord injury) that may affect your ability to be physically active? ☐ **YES**

Some people may worry if they have a medical or physical condition that physical activity might be unsafe. In fact, regular physical activity can help to manage and improve many conditions. Physical activity can also reduce the risk of complications. A Qualified Exercise Professional (QEP) can help with specific advice for physical activity that is safe and that takes your medical history and lifestyle into account.

After reading the ADVICE for your YES response, go to Page 2 of the *Get Active Questionnaire* – ASSESS YOUR CURRENT PHYSICAL ACTIVITY

WANT ADDITIONAL INFORMATION ON BECOMING MORE PHYSICALLY ACTIVE?

- **csep.ca/certifications**
  CSEP Certified members can help you with your physical activity goals.
- **csep.ca/guidelines**
  Canadian Physical Activity Guidelines for all ages.

© Canadian Society for Exercise Physiology, 2017. All rights reserved.

Health Promotion Board

Find out more about

My Healthy Plate

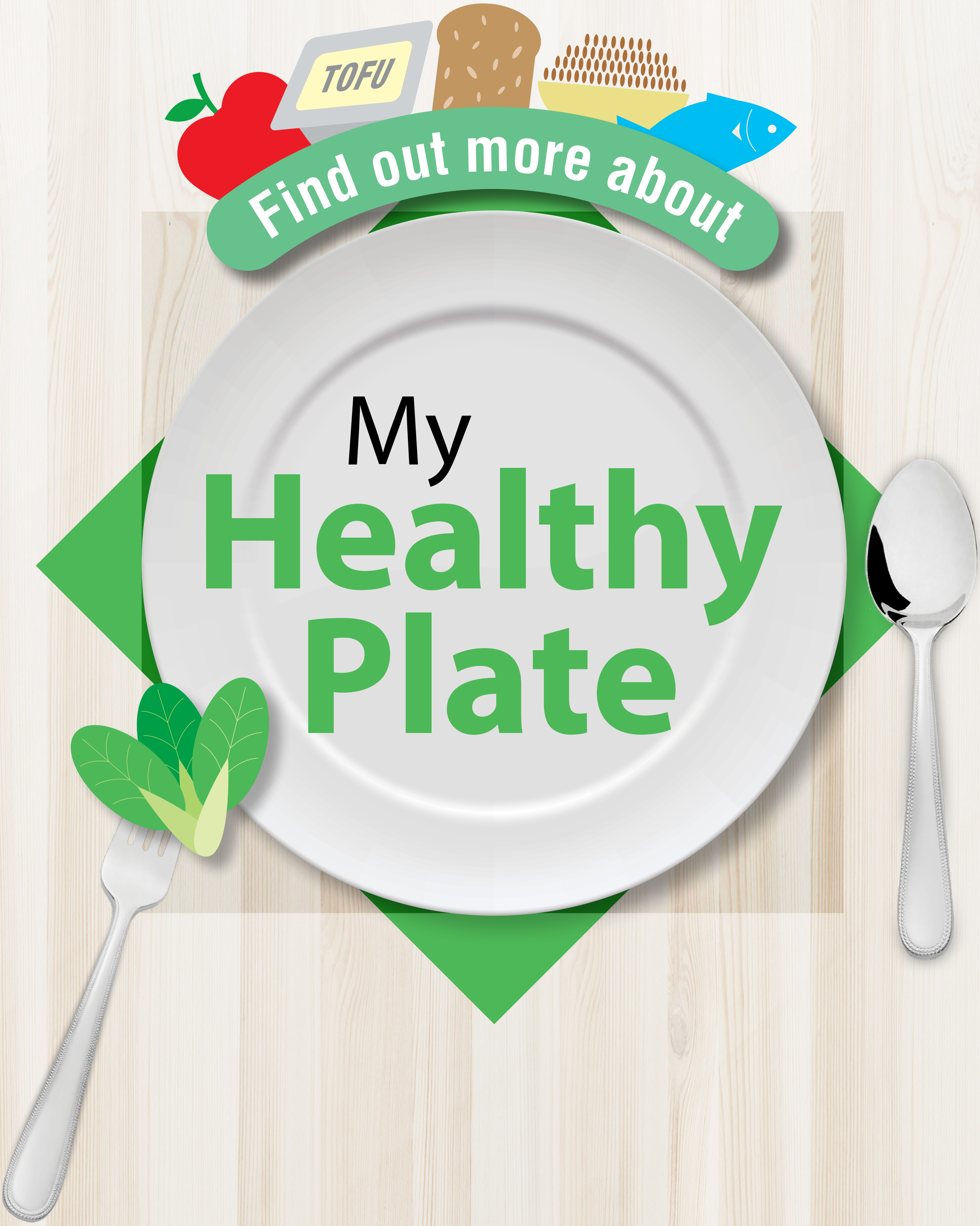

Contents

What is My Healthy Plate? 3

Why was My Healthy Plate developed? 4

Who can use My Healthy Plate?.. 5

How do I apply My Healthy Plate to my diet? 6

Q&As on My Healthy Plate 11

Healthy Diet Pyramid and My Healthy Plate 19

What is My Healthy Plate?

My Healthy Plate is a friendly, easy-to-understand visual tool designed by the Health Promotion Board (HPB) specifically for Singaporeans. You can use My Healthy Plate to help remember and practise healthy habits that can aid with weight control and protect against chronic diseases such as diabetes.

The key healthy habits communicated by My Healthy Plate are:

- Fill half your plate with fruits and vegetables
- Fill a quarter of your plate with whole-grains
- Fill a quarter of your plate with meat and others
- Use healthier oils
- Choose water
- Be active!

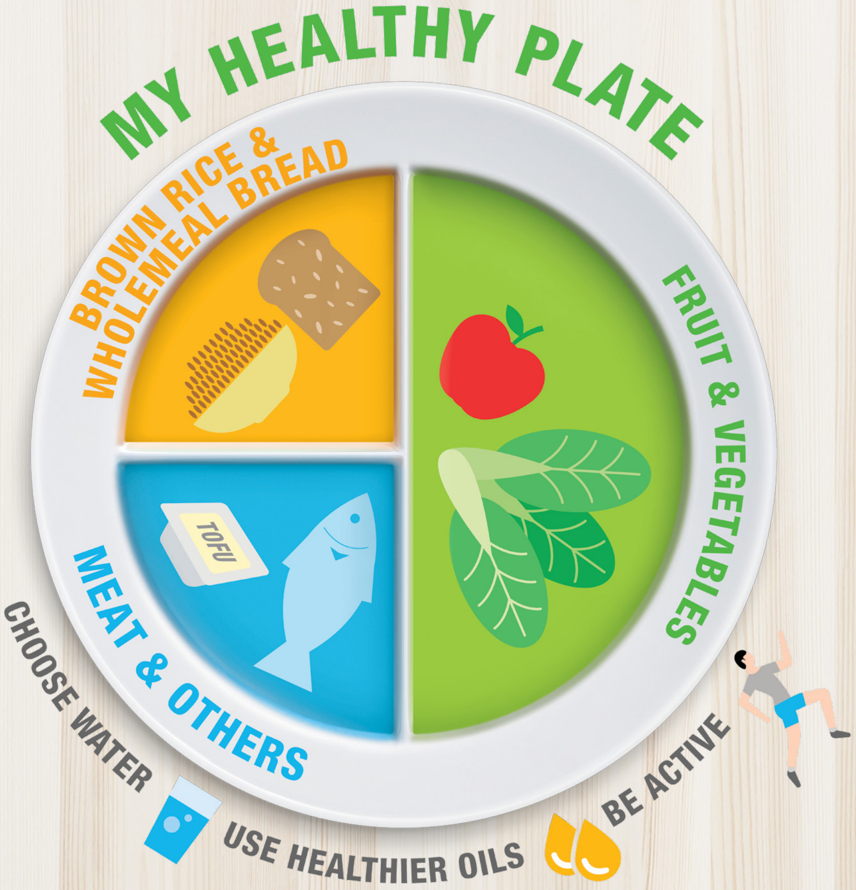

Why was My Healthy Plate developed?

The increasing prevalence of obesity and chronic diseases such as diabetes is a major health-related issue facing Singapore. The 2010 National Health Survey showed that about one in every nine Singaporeans aged 18 - 69 years is obese (Body Mass Index $\geq 30\ kg/m^2$), a 57% increase from the previous survey in 2004. As obesity increases, the likelihood of various diseases, particularly heart diseases, diabetes and certain types of cancer, also increase.

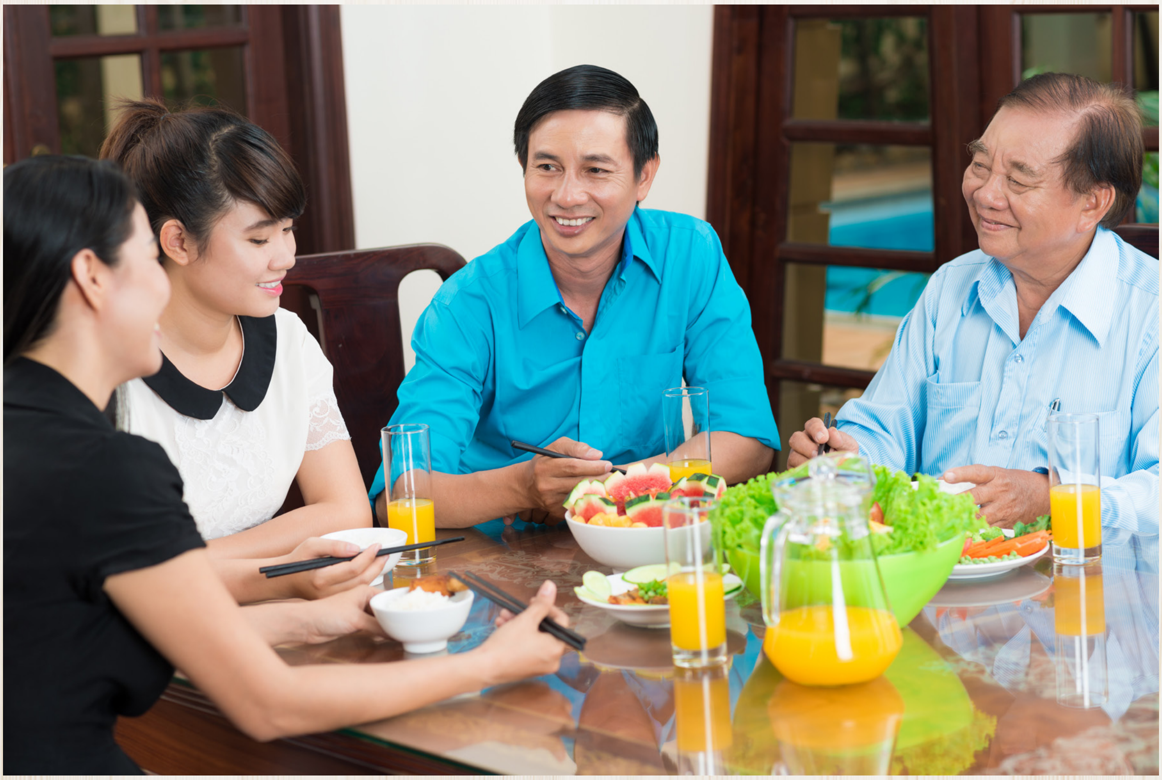

Findings from the 2010 National Nutrition Survey indicate that Singaporeans currently consume mostly refined carbohydrates and nine in ten Singaporeans do not eat enough fruit and vegetables. This survey also indicates that Singaporeans need to change to healthier types of fats and oils and reduce their overall calorie intake.

From a study carried out by HPB, we found that Singaporeans preferred a graphical message, such as a plate-based image, that could be easily associated with food and meals, provided clear and simple messages and that was easy to read and understand.

My Healthy Plate was then developed to better communicate to Singaporeans the latest science-based recommendations on healthy eating habits that can help with weight control and help protect against chronic diseases.

My Healthy Plate aims to provide an easy-to-understand visual representation of what a healthy meal may look like, with the emphasis on increasing fruit and vegetable intake and changing rice, noodles, bread and other carbohydrate-rich foods to whole-grain options.

My Healthy Plate reminds us to adopt healthy habits such as choosing water over sweetened beverages, choosing foods prepared with healthier oil and being physically active.

Who can use My Healthy Plate?

Anyone can use My Healthy Plate. Individuals with specific dietary requirements or existing medical conditions are advised to check with their health care providers on whether My Healthy Plate can meet their specific dietary requirements.

How do I apply My Healthy Plate to my diet?

My Healthy Plate shows you what to eat in the correct proportions to have a well-balanced and healthy diet. My Healthy Plate aims to simplify healthy eating advice by providing a visual representation of what a healthy meal might look like. It aims to serve as a reminder to build balanced meals and is not intended as a mandate to include all food groups at every meal, or eat in a compartmentalised manner.

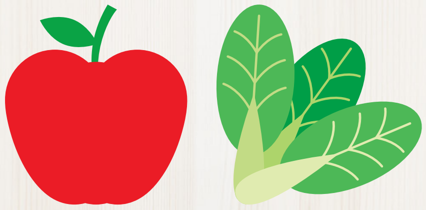

Fill half of your plate with fruit and vegetables

The apple and green leafy vegetables serve as a reminder to eat fruit and vegetables. An apple and green leafy vegetables were chosen because they are commonly eaten by Singaporeans, are easily recognisable and are associated with fruit and vegetables respectively.

Fruit and vegetables are rich in dietary fibre, vitamins, minerals, and can lower the risk of developing heart disease, stroke and certain types of cancer. These pictures can also remind us to choose brightly-coloured fruit and vegetables. We should eat a balanced variety of many different types of fruit and vegetables as this can help us get the nutrients we need.

It matters how your fruit and vegetables are prepared. Eating vegetable dishes with lots of oil or fruit with sugar syrup makes it more likely to consume too many calories and gain weight.

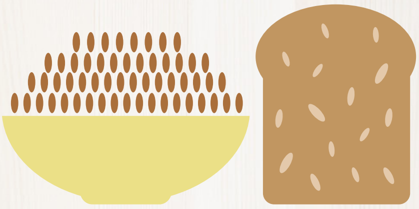

Fill a quarter of your plate with whole-grains

Brown rice and wholemeal bread are chosen as these are commonly available types of whole-grain products found in Singapore. Brown rice and wholemeal bread are also more easily understood compared to the term "whole-grains".

Whole-grain products also include whole-grain noodles, wholemeal pasta, wholemeal biscuits, wholemeal chapatti, and oats.

Whole-grain foods contain vitamins (vitamins B and E), minerals (iron, zinc and magnesium), phytochemicals (lignans, phytosterols) and inulin (a type of dietary fibre).

Consuming whole-grains over refined grains* can reduce the risk of developing heart disease and diabetes and helps you manage your weight as you get hungry less easily. Refined grain products fill the same "quarter" of your plate as brown rice or wholemeal bread, but it is better to choose whole-grains when possible.

**Refined grains such as white rice or white bread have gone through processing which removes the valuable nutrients whole-grains have to offer.*

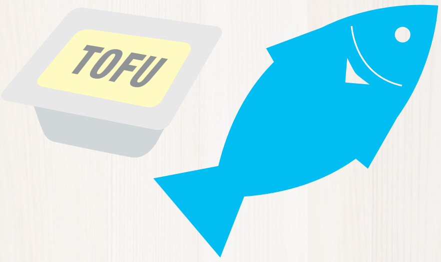

Fill a quarter of your plate with meat and others

The pictures of fish and tofu represent the category of meat and others. This category includes poultry such as chicken, duck and turkey, meat such as beef, mutton and pork, seafood, eggs and nuts as well as bean products and dairy products.

Fish and tofu were chosen as the latest scientific consensus shows that fish, especially oily fish and bean products such as tofu are healthier forms of protein. People who eat these foods in the right amounts are less likely to suffer from cardiovascular disease, one of the major causes of death in Singapore.

Lean meats such as chicken and fish give you more protein per calorie than fatty meats. Protein helps to build and repair tissues in the body. Oily fish such as tuna, mackerel, tenggiri batang and ikan tenggiri papan also contain omega-3 fatty acids, a beneficial fat that supports overall heart health. **Aim for 2 servings of fish a week.**

Ensure you also **include calcium-rich foods in your diet** each day. Milk, yoghurt, cheese, tofu, sardines and calcium-fortified soy milk are rich in calcium. Choose low-fat or non-fat dairy products more often than full-fat dairy products to help maintain a healthy weight. However, low-fat or non-fat milk and other dairy products are not suitable for children below the age of 2 years, as they have higher energy needs for rapid growth.

Use Healthier Oils

Healthy fats should be included in the diet on a daily basis to achieve optimal health. While cooking at home, use healthier oils such as canola, olive, soy, sunflower and peanut.

These contain more of the healthier monounsaturated and polyunsaturated fats that can decrease the risk of developing heart disease. When eating out, look out for hawkers and restaurants that use healthier cooking oil.

While healthier oils have heart benefits, they still contain the same amount of calories as regular oils and should be consumed in moderation.

Choose water

Make water your drink of choice. Drinking too many sugar-sweetened drinks may make you gain weight. Choosing water over sugar-sweetened drinks helps you maintain a healthy weight.

Adding white sugar, brown sugar, rock sugar, honey or other types of sugar to any drink (e.g. coffee or tea) makes it a "sugar-sweetened drink".

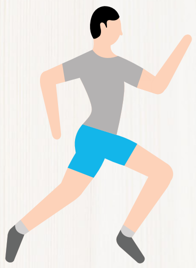

Be Active!

Being physically active is an essential part of a healthy lifestyle that prolongs good health. Activities like brisk walking, cycling and swimming, daily lifestyle activities (e.g. taking the stairs, doing household chores) and strength-training (e.g. using hand weights, doing qigong or yoga) all count as physical activity.

- For infants, physical activity should be encouraged from birth, particularly through floor-based play in safe environments.
- Children below 7 years who are able to walk on their own, should be physically active for at least 180 minutes spread throughout each day in safe environments.
- Children and youth aged 7 to 18 years should accumulate 60 minutes or more of moderate-to-vigorous-intensity physical activity every day.
- Adults should aim for 150 minutes of physical activity each week.

What if I don't follow My Healthy Plate at each meal?

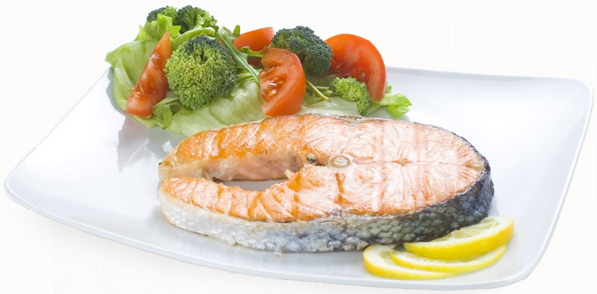

My Healthy Plate is a guide to help you meet dietary recommendations more easily. It is good to try to follow the My Healthy Plate at each meal. However, if you are unable to do so, plan your meals such that you eat food from all four food groups throughout the day.

What about snacks?

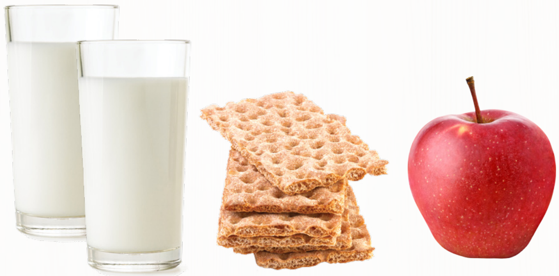

Some individuals may need snacks between their meals depending on their calorie requirements. When choosing snacks, go for healthy options such as whole-grain foods, fruit and vegetables and dairy or calcium-rich food. Try to choose food low in salt, sugar and unhealthy fats. If a previous meal didn't contain enough of a particular food group, your snack can be used to make up for this.

For example, if your meal didn't contain fruit or vegetables, the subsequent snack may be a piece of fruit or some vegetable sticks.

Why is physical acitivity included in My Healthy Plate?

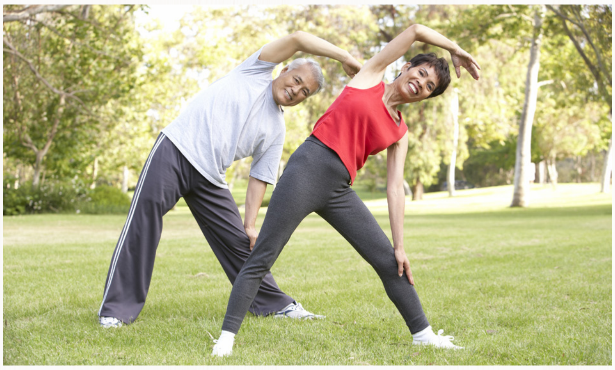

Diet and physical activity are both important in helping with weight control and protecting against chronic diseases. As My Healthy Plate is intended to go beyond a focus on diet to highlighting the importance of energy balance (i.e. calories consumed need to be balanced with calories expended for weight management), the reminder to "Be Active!" was included.

What other dietary recommendations should Singaporeans take note of?

Other dietary recommendations include limiting salt and sugar intake and moderating alcohol intake.

Limit intake of salt

Singaporeans should limit their daily intake of salt to 1 teaspoonful (about 5 grams of salt or 2000 mg of sodium). Younger children may require less than 1 teaspoon of salt (Table 1). This applies to salt from sauces, table salt, and processed foods.

Table 1: Recommended salt limits

Age	Recommended limit (grams)
6 months (181 days) - 12 months	1
1 – 6 years	2.5
7 – 18 years	5
Above 18 years	5

Limit intake of sugar

Singaporeans should also limit their daily intake of added sugar to 11 teaspoons. Younger children may require less (Table 2). Added sugar can be found in sweetened drinks, fruit juice, honey, jam, processed food such as sweets.

Table 2: Recommended sugar limits

Age	Recommended limit (teaspoons)
6 months (181 days) – 12 months	5
1 – 2 years	7
3 – 6 years	9
7 - 18 years	11
Above 18 years	11

Limit intake of alcohol

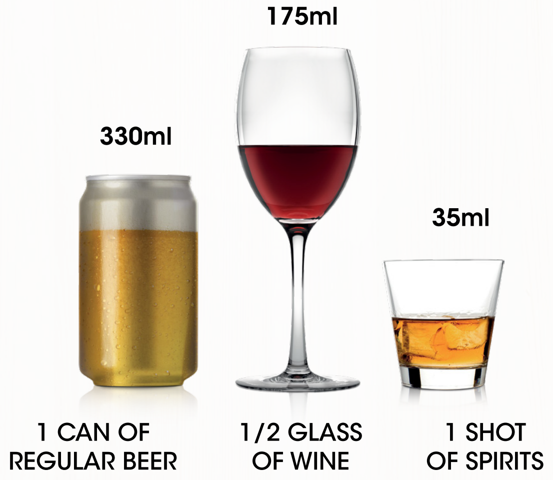

For alcohol, there isn't any level of drinking which can be guaranteed as completely safe or without risks. There are also some groups of people who should avoid drinking completely – such as children/adolescents*, those who are driving or engaged in activities which require attention, skills or coordination, pregnant mothers or those trying to conceive, or those who have medical conditions such as asthma, alcohol allergy, or liver disease.

For healthy adult Singaporeans, men should not drink more than 2 standard drinks a day and women no more than 1 standard drink a day.

A standard alcoholic drink contains 10 grams of alcohol. This roughly equates to:

- A can (330 ml) of regular beer with 5% alcohol content
- Half glass (175 ml) of wine with 15% alcohol content
- A shot (35 ml) of spirits with 40% alcohol content

It is not enough to just note the volume of alcohol consumed, but the percentage of alcohol in the drinks as well.

*Do note that in Singapore, it is illegal for anyone under the age of 18 years to purchase alcohol.

Are the relative sizes of My Healthy Plate sections based on calories or number of servings?

My Healthy Plate provides a visual representation of what a healthy meal might look like and illustrates the approximate relative proportions of each food group that should be included in a healthy meal.

My Healthy Plate does not define a certain number of calories or servings per day from each food group as the actual portion size or amount of different foods needed varies between individuals (please refer to the recommended number of servings and serving size chart to identify the right amount of food for you).

Food Groups	Recommended number of servings per day							
	6 months (181 days) - 12 months	1 – 2 years	3 – 6 years	7 – 12 years	13 – 18 years	19 – 50 years	51 years and above	Pregnant & lactating women
Brown Rice & Wholemeal Bread	1 – 2	2 – 3	3 – 4	5 – 6	6 – 7	5 – 7	4 – 6	6 – 7
Fruit	½	½ - 1	1	2	2	2	2	2
Vegetables	½	½	1	2	2	2	2	3
Meat & Others	2	2	2	3	3	3	3	3½
of which *Dairy Foods or Calcium-Rich Foods*	*1½*	*1½*	*1*	*1*	*1*	*½*	*1*	*1*

For infants aged 6 months – 12 months, their dairy foods or calcium-rich foods servings should be provided in the form of 750ml breast milk or infant formula.

What is an example of 1 serving of Brown rice and Wholemeal Bread?

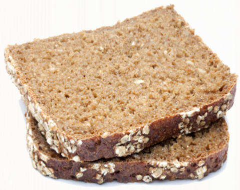

2 slices of wholemeal bread (60g)

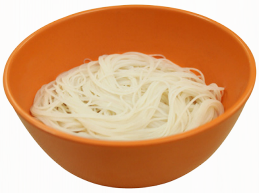

½ bowl* whole-grain noodles, beehoon or speghetti (100g)

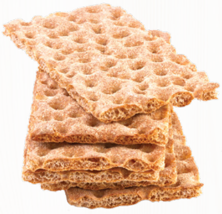

4 plain wholemeal crackers (40g)

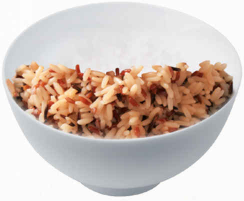

½ bowl* of rice/brown rice (100g)

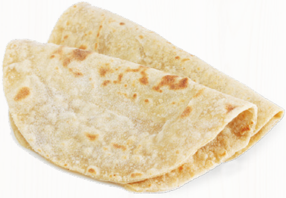

2 wholemeal chapatis (60g)

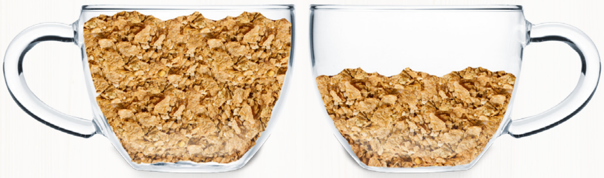

1½ cups** whole-grain breakfast cereal(40g)

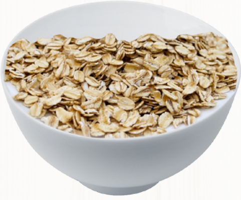

2/3 bowl* of uncooked oatmeal (50g)

All weights listed are for edible portions only.

* 1 rice bowl
** 250ml cup

What is an example of 1 serving of Fruit?

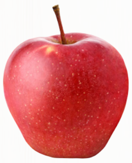

1 small apple, orange, pear or mango (130g)

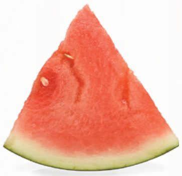

1 wedge of papaya, pineapple or watermelon (130g)

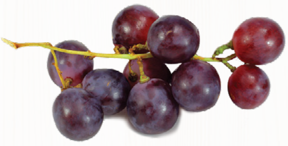

10 grapes/longans (50g)

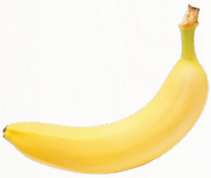

1 medium banana

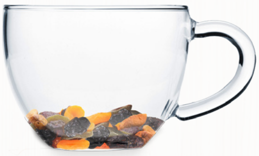

¼ cup** of dried fruit (40g)

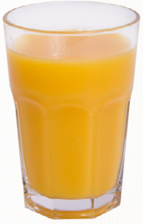

1 glass* pure fruit juice (250ml)

What is an example of 1 serving of Vegetables?

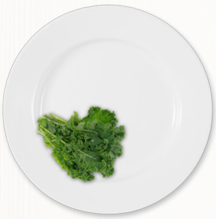

¼ round plate+ of cooked vegetables

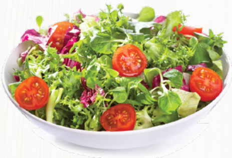

150g raw leafy vegetables

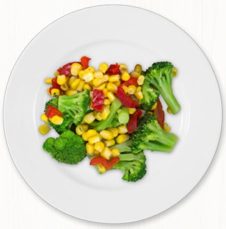

100g raw non-leafy vegetables

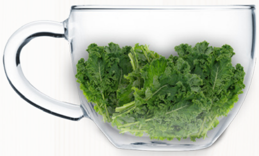

¾ mug*** of cooked leafy (100g)

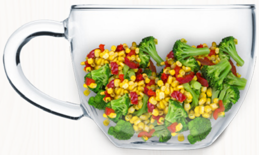

¾ mug*** non-leafy vegetables (100g)

All weights listed are for edible portions only.

- * 250ml glass
- ** 250ml cup
- *** 250ml mug
- \+ 10 inch plate

What is an example of 1 serving of Meat and Others?

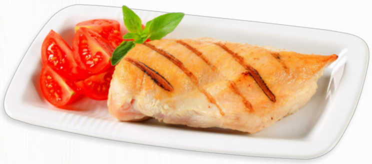

1 palm-sized piece of meat, fish or poultry (90g)

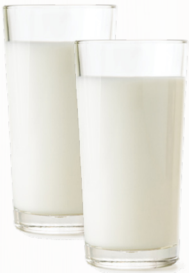

2 glasses* of reduced-fat milk (500ml)

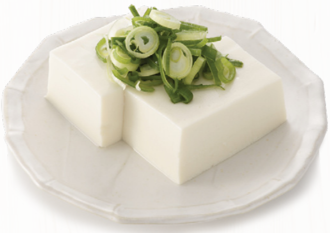

2 small blocks of soft beancurd (170g)

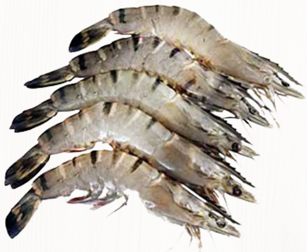

5 medium prawns (90g)

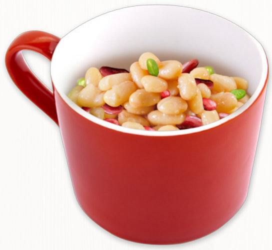

¾ cup** of cooked pulses (peas, beans, lentils) (120g)

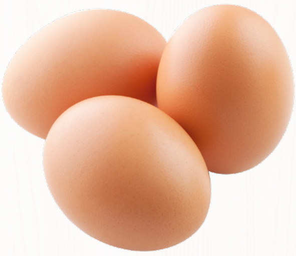

3 eggs (150g)

All weights listed are for edible portions only.

*	250ml glass
**	250ml cup

Healthy Diet Pyramid and My Healthy Plate

With My Healthy Plate, does it mean that we no longer use the Healthy Diet Pyramid?

Both my Healthy Plate and the Healthy Diet Pyramid are tools used to convey healthy eating messages to Singaporeans. The information about what and how much to eat has essentially remain unchanged. Both the Healthy Diet Pyramid and My Healthy Plate are illustrations based on the same food groups and recommendations about what and how much to eat. Moving forward, My Healthy Plate will be used as an easy-to-understand visual guide to communicate HPB's healthy eating messages and to help Singaporeans remember and practise healthy habits.

What are the differences between My Healthy Plate and the Healthy Diet Pyramid?

My Healthy Plate and the latest version of the Healthy Diet Pyramid (2009) share some commonalities; much of the scientific information used to develop both is substantially the same.

My Healthy Plate presents important information on diet in a simpler, easier-to-understand manner and places greater emphasis on important habits to help with weight control and to protect against chronic diseases.

<table>
<tr><th>Healthy Diet Pyramid</th><th>My Healthy Plate</th></tr>
<tr><td>Rice and Alternatives

The Healthy Diet Pyrmaid shows both whole-grain foods and refined grain foods</td><td>Brown Rice & Wholemeal Bread

My Healthy Plate emphasises the recommendation to choose whole-grain products where possible by showing only whole-grain versions of commonly consumed grain products rather than refined grain products.</td></tr>
<tr><td>Fruits</td><td rowspan="2">Fruit & Vegetables

Fruit and Vegetables are still two separate food groups.
Both Fruit and Vegetables are placed together in one section of My Healthy Plate to emphasise to Singaporeans the importance of eating fruit and vegetables.</td></tr>
<tr><td>Vegetables</td></tr>
</table>

Healthy Diet Pyramid	My Healthy Plate
**Meat and Alternatives**<br><br>States the need for calcium-rich foods, but not clearly. Graphics show examples of foods in this food group, but don't focus on good choices.	**Meat & Others**<br><br>My Healthy Plate graphics emphasises healthier choices within the Meat & Others food group, namely fish (especially oily fish), bean products and calcium-rich foods such as tofu.
**Fats, Oils, Sugar and Salt**<br><br>The Healthy Diet Pyramid recommends that these should be used in small amounts, but doesn't provide positive messaging on what Singaporeans should do.	**Use Healthier Oil**<br><br>My Healthy Plate encourages Singaporeans to choose foods made with healthier types of oil rather than to simply use all types of oil and fat in small amounts. Reducing oil and fat intake usually means increasing carbohydrate intake, which in Singapore, means eating proportionately more highly refined carbohydrate foods. Emerging evidence shows that a moderate amount of fat in the diet is beneficial for health, especially if fat consumed is the healthier unsaturated fat. However, 7 in 10 Singaporeans consume too much saturated fat, hence the need to emphasise the quality of fat consumed rather than to consume less fat in general.<br><br>**Choose Water**<br><br>My Healthy Plate encourages Singaporeans to Choose Water over sugar-sweetened drinks. Water, or other drinks like coffee and tea with no sugar, contains no calories.
**(Not included in the Healthy Diet Pyramid)**	**Be Active!**<br><br>My Healthy Plate reminds Singaporeans that physical activity is also an important part of weight management.

Mind Matters

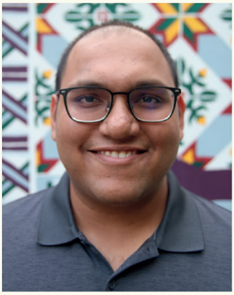
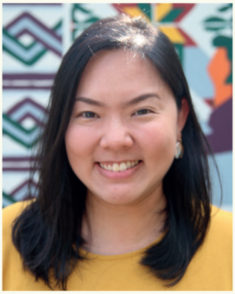
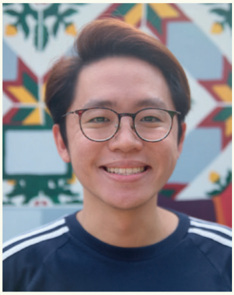
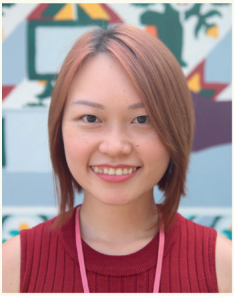

A Resource Directory on Community Mental Health

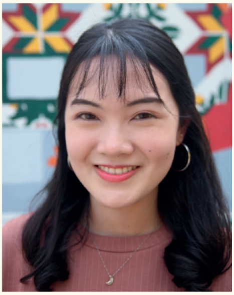
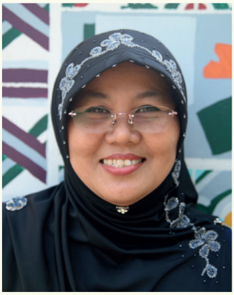
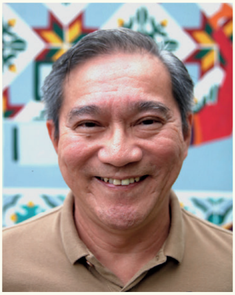
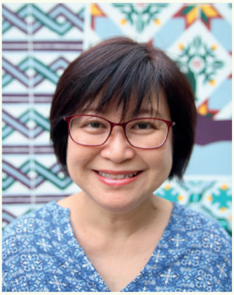

aic
agency for integrated care

MENTAL HEALTH AWARENESS SINGAPORE

RESOURCE DIRECTORY

A.	About the Resource Directory	1
B.	About the Community Mental Health Masterplan	1
C.	Network of Care and Support	2
D.	Myths & Facts	4
E.	Common Scenarios You May Come Across	6
F.	Frequently Asked Questions	10
G.	Important Contact Details	15
H.	Available Resources	16
I.	Community Partners Near You	18
J.	Hearing from Community Partners	19

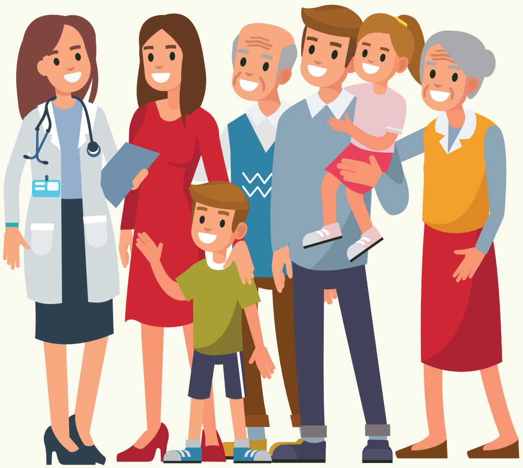

A. About the Resource Directory

This "Mind Matters" Resource Directory provides an overview of the Community Mental Health Masterplan including the available and accessible community mental health services and resources to support you and your loved one in this journey.

This directory also gives examples of common situations, myths and facts about persons with mental health conditions, answers to common questions on mental health, helplines and details of community partners located in the neighbourhood.

We hope this directory will increase your understanding of the common mental health issues and guide you on where to seek help when needed. While all information in this resource directory is accurate at the time of print, it is still recommended that you seek the advice of medical professionals for specific care needs.

B. About the Community Mental Health Masterplan

Since 2012, the Agency for Integrated Care (AIC) was appointed by the Ministry of Health to develop the Community Mental Health (CMH) Masterplan. The plan focuses on working closely with the health, social and community care partners to build an integrated mental health network to better support persons at risk/with mental health issues including dementia and their caregivers in the community.

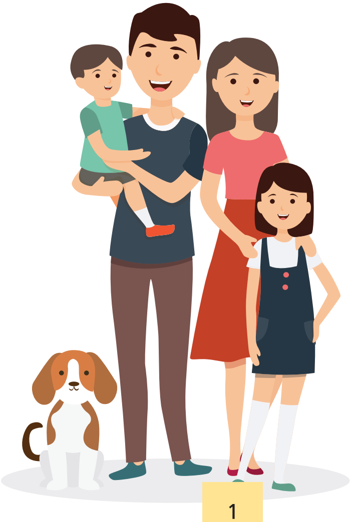

c. Network of Care and Support

You can strengthen your mental well-being through coping strategies such as finding ways to de-stress and relax. If you need help managing stress or mental health issues, do seek help early. And if you are a caregiver, there is support available for someone with a mental health condition. Here are some examples of the support you can find near home.

How can you prevent mental health issues?

How can you get care and support?

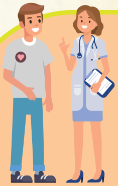

COMMUNITY INTERVENTION teams work with doctors to provide additional support.

VISIT A GP, or a doctor at the polyclinic / specialist outpatient clinic for consultation, assessment and treatment near home.

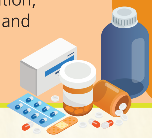

SEEK HELP from community intervention teams that provide psychotherapy, basic emotional support, caregiver education and care coordination.

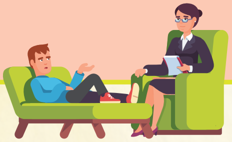

If you are a caregiver

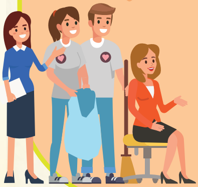

SUPPORT one another in your recovery journey by joining support groups formed by various community organisations.

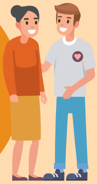

RELIEVE the stress of caring for loved ones at home by tapping on caregiver support and respite services such as the eldersit service.

CONSIDER dementia or psychiatric day care for your loved one so you can work with a peace of mind.

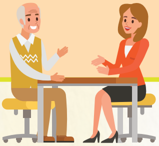

What should you do if you feel mentally unwell

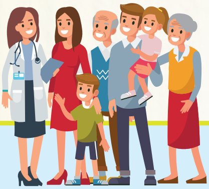

LEARN how to stay mentally "fit" through courses and talks on topics like mindfulness, stress management and family relationships building.

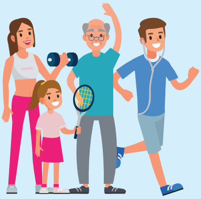

STAY ACTIVE and make new friends by taking part in activities at your nearest wellness centres.

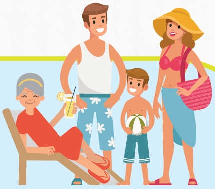

STAY IN TOUCH with your family and friends! Go out and take part in hobbies together.

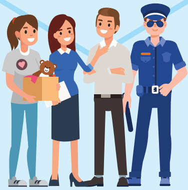

REACH OUT to the community for support, e.g. community outreach teams. Many frontline staff from the grassroots, government and corporate organisations are trained to provide basic support on mental health and dementia.

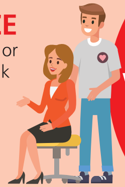

SEEK ADVICE from a social worker or counsellor if you think you or someone is mentally troubled or feels overwhelmed.

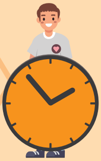

STAY-IN CARE and support is available at nursing homes and psychiatric rehab centres if you are unable to care for your loved one at home.

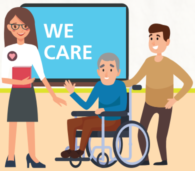

GO FOR caregiver training courses to learn how to better care for your loved one instead of worrying you are doing it poorly.

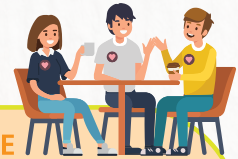

TAKE CARE of your own wellbeing. Talk to other caregivers for emotional support by joining a caregiver support group. Dementia-Friendly Communities have caregiver support networks that organise activities for caregivers that emphasise self-care.

If you need more information and resources, do write to **ccmh@aic.sg**

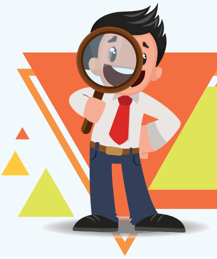

D. Myths & Facts

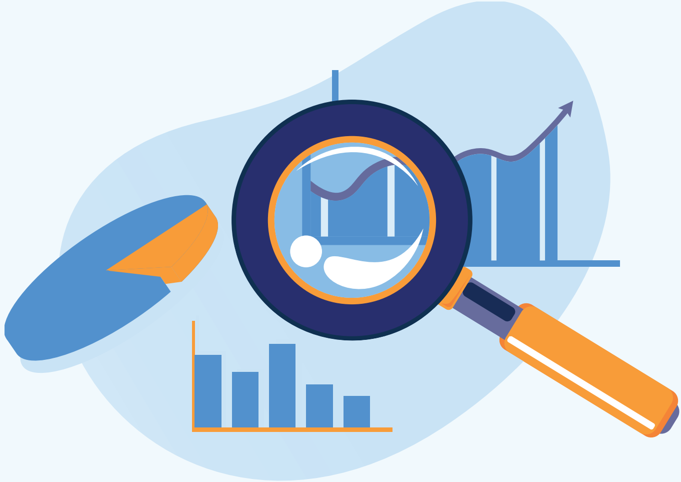

Mental health conditions are common and can affect anyone. According to the Singapore Mental Health Study (2016), 1 out of 7 people in Singapore will develop a mental health condition over the course of their life. Major Depressive Disorder, Alcohol Abuse and Obsessive Compulsive Disorder were found to be the top three most common mental health conditions in Singapore.

Here are some myths and facts about them to help you understand mental health better.

Myth **A person with a mental health condition is violent and unpredictable.**

Fact In reality, the vast majority of those who have a mental health condition are no more violent than anyone else. You may already know someone with a mental health condition but do not even realise it.

Myth Once a person develops a mental health condition, he/she will never recover.

Fact Studies have showed that most people with a mental health condition get better, with many making a full recovery. For some, recovery is the ability to live a fulfilling and productive life. For others, recovery suggests the reduction of symptoms.

Myth Persons with any mental health conditions should be kept in hospital.

Fact With appropriate treatment and support, persons with mental health conditions can still work and lead meaningful lives. Majority can live independently in the community.

Myth I can’t do anything to support someone with a mental health problem.

Fact There are many things you can do to make a difference to their lives:

- Check in frequently and listen
- Take time to learn about their condition and don't judge
- Treat them in the same way as you would to others
- Direct them to seek professional help (e.g. Community Intervention Team)

Myth Dementia is part of the normal ageing process.

Fact This is not true. Dementia is an illness that affects the brain, resulting in memory loss, difficulties with language, recognition, planning and performing complex tasks. One in 10 seniors aged 60 and above has dementia and it is not part of the normal ageing process.

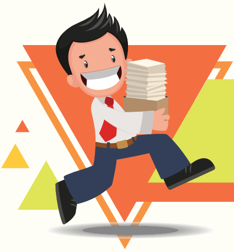

E. Common Scenarios You May Come Across

Dear Mind Matters,

I noticed that my elderly neighbour has not been herself lately. She has been getting lost in the neighbourhood and had to be accompanied back home by other neighbours or even the police who have found her wandering around.

Her only son who doesn't stay with her mentioned to me that she has been very forgetful as well, forgetting to switch off the stove and misplacing things. How can I help her?

Regards,
Concerned Neighbour (Resident)

Dear Concerned Neighbour (Resident),

From what you described, your neighbour seems to be exhibiting the signs and symptoms of dementia. Dementia is a condition that affects the brain and it is not due to normal ageing. It affects cognitive abilities and personality.

You may advise her son to take note of his mother's unusual behaviour and encourage him to bring her for an assessment. This can be done at the polyclinic or restructured hospital which she frequents for her regular check-ups.

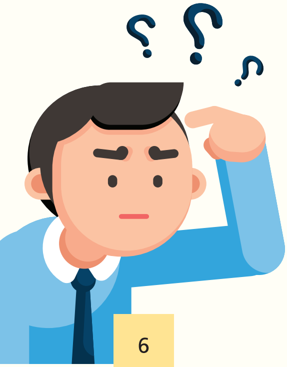

ABCD Signs and Symptoms of Dementia

Activities of Daily Living – Difficulty performing daily tasks such as dressing, bathing or eating etc

Behaviours – Changes in personality or withdrawal from social activities

Cognition and Coordination – Memory loss that affects daily life, decreased or poor judgement, planning and problem solving

Disorientation – Confusion with time and place

Dear Mind Matters,

I am 43 years old and work as a purchasing manager in a manufacturing company. There are a lot of rumours about the company planning to move its operations out of Singapore which will result in a mass retrenchment. I have been very worried about this and I have not been sleeping well for the past few months. I am the sole breadwinner of my family and have 2 children to support. If I lose my job, I won't be able to find a new job so easily. I feel lethargic and have not been eating well. My work has been affected. My colleagues are trying to cheer me up but I don't feel like talking to them.

Regards,
Troubled Office Worker

Dear Troubled Office Worker,

It's normal to feel sad or worry from time to time and the feeling of sadness usually goes away after a short period of time. However, if the feeling of sadness or low mood persists for weeks and is starting to affect your daily life and functions, you may be showing signs of possible depression. You should talk to a counsellor or a community mental health partner who can support your emotional needs. You can refer to the list of community mental health partners and polyclinics found in the insert on 'Support and Services for Mental Health Issues and Dementia' for more information.

Common Signs and Symptoms of Depression

- Persistent feelings of sadness, emptiness
- Loss of interest or pleasure in activities
- Angry outbursts, irritability or frustration over small matters
- Feeling tired
- Loss of appetite or increased appetite (weight loss or weight gain)
- Slowed thinking and not able to concentrate

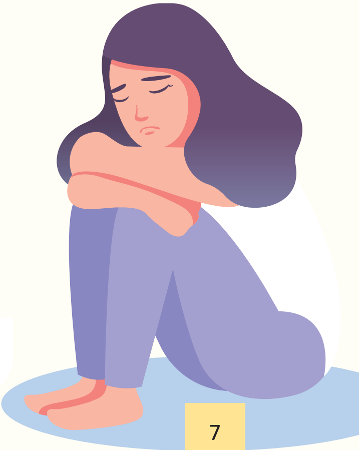

Dear Mind Matters,

My teenage daughter is normally quiet and only has a few good friends from school. She studies hard and does well in school. She doesn't like some girls in her class as they are posting unkind comments on her Facebook page. We advised her to ignore them and focus on her studies.

However, she recently has been having tummy aches in the morning and is not able to go to school. She has also complained that her heart races every morning before school. We have seen the GP and TCM doctor several times but the symptoms don't seem to go away. She has been missing a lot of school and worries about how it will affect her grades. She is feeling increasingly very lousy about herself.

Best regards,
Worried Mother

Dear Worried Mother,

A person will go through a lot of changes in their teenage years – physically and emotionally. Teenagers tend to become more self-conscious and will be affected by how others view them. She may be feeling anxious about going to school to face her classmates who have bullied her online.

Listen to your daughter attentively and show that you empathise with her situation. A supportive family environment will help her go through these changes and transitions in life. Share with her that you know that this is really hard for her and that you are here for her because you care.

If her condition persists, you may want to seek advice from a professional such as a child psychologist or the Community Health Assessment Team (CHAT) who can assess your daughter's condition and provide advice on treatment. You can find out more about them at www.chat.mentalhealth.sg

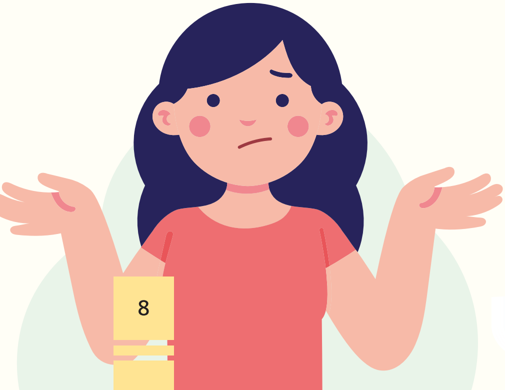

Common signs & symptoms of Anxiety

- Upset stomach
- Avoiding school
- Constant feeling of worry and stress
- Withdrawal from social activities
- Panic attacks - rapid heartbeat, cold sweats, breathlessness

Dear Mind Matters,

I have a neighbour from hell! He keeps accusing me of spying on him and trying to poison him. He even reported me to the police on several occasions and the police found no proof to support his claim. When the police visit him, he would complain that his home is not safe and that they have been sent by the government to install cameras in his house. While he doesn't harm anyone, he sometimes would shout outside my door and scare my children. Moving away is not an option for me. Can you help?

Best regards,
Distressed Resident

Dear Distressed Resident,

From your description, it sounds like your neighbour may need mental health support. In the meantime, when interacting with your neighbour, be patient and stay calm. Don't challenge or try to correct his inaccurate beliefs as it may anger or frustrate him further. Don't take his aggressive behaviour personally as his emotions and worries feel very real to him!

Common signs & symptoms of Schizophrenia

- Delusions
- Hallucinations
- Paranoid
- Hearing voices that don't exist
- Disorganised thinking and speech

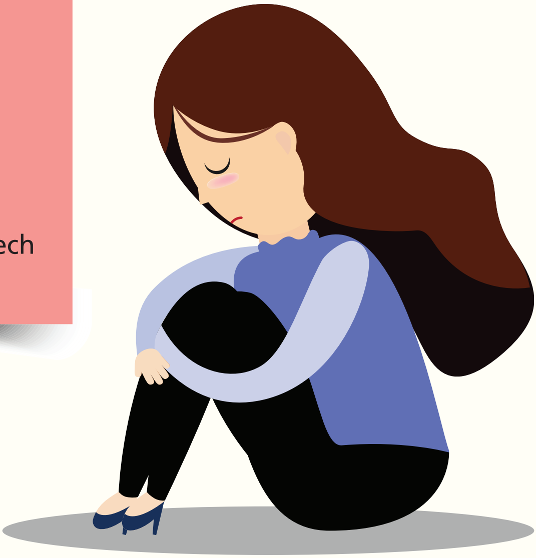

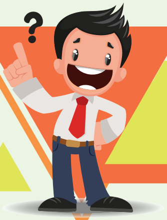

F. Frequently Asked Questions

I: General Mental Health

1. Are mental health conditions curable?

Recovery is possible. A recovered person is able to lead a regular life like anyone else - holding job(s), spending time with friends and family. The process of recovery will require support from friends and family, and may also involve help from healthcare professionals, counselling and medication. For more information on mental health conditions and their treatments, please visit www.aic.sg

2. Are there any subsidies for treating mental health conditions?

Here are some financial schemes available to clients to defray the cost of mental health services. Some insurance policies may also be able to cover the cost of mental health services. Check with your insurance company.

Subsidy Schemes	Details
**Medisave for Inpatient Psychiatric Episodes**	Up to $150 a day for daily hospital charges, subject to a maximum of $5000 a year<br>www.moh.gov.sg/healthcare-schemes-subsidies/medisave
**Medisave for Outpatient Treatments under Chronic Disease Management Programme (CDMP)**	Up to $500 per Medisave account a year. Subject to 15% co-payment by patient. Covers schizophrenia, major depression, bipolar disorder, dementia and anxiety<br>www.moh.gov.sg/healthcare-schemes-subsidies/medisave
**Medishield Life Inpatient (Psychiatric)**	Up to 60 days per policy year<br>$160 per day<br>www.moh.gov.sg/home/our-healthcare-system/medishield-life/what-is-medishield-life/what-medishield-life-benefits

3. I feel dizzy after taking the medicine and want to stop. Is this advisable?

Common side effects of psychiatric medications include gastric discomfort, drowsiness, weight gain, dry mouth, mood swings and restlessness. However, the side effects of the medications vary with individuals. You should consult your doctor before switching or stopping your medications. The doctor will be able to adjust the dosage for you.

4. Will a person with a mental health condition be charged if they are found guilty of a criminal offence?

Having a mental health condition does not automatically excuse a person from being found guilty of a criminal offence. It depends on the state of mind at the time of the offence. The person will usually be examined by a psychiatrist and the outcome is for the court to decide.

II: Employment Opportunities for Persons with Mental Health Conditions

5. Can someone with a mental health condition be employed?

There are services which help to prepare individuals to re-enter the workforce. Here are ways which persons with mental health issues can seek employment support.

Employment Support Programmes

**Care Corner Singapore**<br><br>**Tel: 6250 6813**<br><br>**Email: ccs@carecorner.org.sg**	**Employee Assistance Programme (EAP)** provides workplace support for employees with or at risk of having mental health issues through a multi-disciplinary team consisting of professionals and peer support specialists.
**Institute of Mental Health**<br><br>**Tel:**<br>**6566 7462 (OcTAVE@Bukit Batok)**<br>**6479 7353 (OcTAVE@Cantoment)**<br>**6389 2904 (OcTAVE@IMH)**<br>**6389 2678 (Job Club)**	**Occupational Therapy: Activities, Vocation and Empowerment (OcTAVE)** offer a range of outpatient rehabilitation programmes aimed at promoting community independence, providing therapeutic support, nurturing clients' potential and facilitating their recovery.<br><br>*cont. next page*

**Email: enquiries@jobclub.sg**	**Job Club** provides employment opportunities assisting individuals with psychiatric conditions in job placement.
**Singapore Anglican Community Services**<br><br>**Tel: 6812 0878**<br><br>**Email: ies@sacs.org.sg**	**Employment Support Services (ESS)** provides employment services that help persons in recovery seek sustained employment in the competitive job market. It also provides the business community with comprehensive employment services to support them in integrating these individuals in the workplace.<br><br>**Employment Internship Programme (EIP)** provides on the job training for persons in recovery from mental health issues to prepare them for open employment.<br><br>**Employee Assistance Programme Plus (EAP+)** provides workplace support for employees with or at-risk of having mental health issues.
**Singapore Association for Mental Health**<br><br>**Tel: 6665 9220**<br><br>**Email: mindsetLH@ samhealth.org.sg**	**MINDSET Learning Hub** works with persons in recovery from mental health issues for their vocational training and employment needs. Individuals are aided in the development of their skills, confidence, financial stability and independence.

III: About Depression

6. If someone is feeling sad after the death of a loved one, is he/she considered depressed?

Grieving from the loss of loved ones is a normal reaction. Usually, these feelings would subside after a period of time. However in certain cases, the person may have low mood or feel sad for extended periods of time, where the feelings are intense and the sadness can persist for weeks, months or even years, interfering with the day-to-day functions of one’s life.

Under such circumstances, further assessments by professionals may be necessary. This person may benefit from speaking to a counsellor (refer to the list of community partners) or to a doctor trained in mental health care (refer to the list of GPs who sees clients with mental health conditions).

7. What are the different ways to treat depression? Can an individual who has depression get better without medication?

It depends on the severity of the condition, coping skills of the individual, and the available support and resources. However, it is advisable to seek advice with one's medical provider to determine the need for medication and treatment options.

IV: Dementia

8. Does one need to go for routine screening for dementia?

Routine screening is only recommended if you notice signs of dementia such as decline in cognitive activities or memory problems (particularly for recent events) that are persistent or worsening.
Consult a gerontologist or visit the memory clinics at the public hospitals if you notice these symptoms.

9. How can I prevent dementia?

Keeping an active mind and healthy lifestyle is advisable, although there is no guaranteed way of prevention. If you have conditions such as high blood pressure, diabetes or high cholesterol, you should ensure they are well controlled as they are risk factors to certain types of dementia.

V: Suicide

10. What can I do if someone I know is contemplating suicide?

Take every suicidal thought of the person seriously. Stay with him/her and listen attentively to let him/her know you are concerned. By letting the person talk about their suicidal thoughts does not increase their risk of committing suicide. Direct them to professional help such as the Samaritans of Singapore (SOS) 24-hour helpline at 1-767 or WhatsApp at 9151 1767. If they are in immediate danger of harming themselves or others, you should call the police at 999 and alert the family members.

11. Are there doctors in the neigbourhood who can treat people with mental health conditions?

Yes, there are General Practitioners (GPs) trained in mental health who are able to provide psychiatric consultation for persons with mental health conditions. Polyclinics can also diagnose and manage mental health conditions. For more information about the locations of the GPs and polyclinics that can support a person with mental health conditions in the community, please refer to the insert on 'Support and Services for Mental Health Issues and Dementia'.

12. There is a resident in the neighbourhood whom I suspect has a mental health condition, how can I help this resident?

For general advice, information and resources, you can write in to ccmh@aic.sg. If it is an emergency, you may call the Police or call the 24-hour Mental Health Helpline run by the Institute of Mental Health at 6389 2222.

13. Is there somewhere in the neighbourhood I can go to if I just need someone to talk to?

There are social service agencies in the community that provide screening, assessment, counselling and therapies for persons with mental health conditions. To find a service near your, refer to the insert on 'Support and Services for Mental Health Issues and Dementia'.

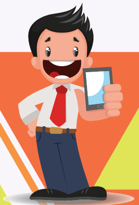

G. Important Contact Details

If you need advice on how to help and support someone you know who may have mental health-related issues, you may contact:

**Email us at**	ccmh@aic.sg
**Call us at**	AIC Hotline<br>1800–650–6060<br><br>Website: www.aic.sg
**Community Partners**	Contact details are available in the insert on 'Support and Services for Mental Health Issues and Dementia'.

For EMERGENCIES, you may contact the following:

**Mental Health Helpline (run by IMH)**	6389 2222 (24-hour)
**Samaritans of Singapore (SOS)**	Hotline: 1-767 (24-hour)<br>CareText Whatsapp: 9151 1767 (24-hour)
**Care Corner Counselling Hotline (Mandarin)**	1800–353–5800<br>Daily (exclude public holidays)<br>10am–10pm
**Police**	999 (24-hour)
**Non-Emergency Ambulance**	1777 (24-hour, fees apply)

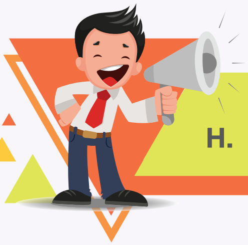

H. Available Resources

Many resources are available through the community partners, at Dementia Go-To Points and can be found online via our website and social media. Below is a list of useful resources for you.

**Mental Health Awareness Singapore Facebook #mhawarenesssg Instagram**	To learn the latest developments in the mental health landscape: www.facebook.com/MentalHealthAwarenessSG/ www.instagram.com/mhawarenesssg/
**Dementia-Friendly Singapore Facebook #heygiffy Instagram**	Stay in touch with latest happenings and developments on Dementia-Friendly Communities, find out about dementia services, resources and learn from other caregivers on providing care to your loved ones: www.facebook.com/DementiaFriendlySingapore/ www.instagram.com/heygiffy/
**Agency for Integrated Care Facebook Page**	This page is an online community for caregivers in Singapore, to share information and resources on providing care for their elderly loved ones. www.facebook.com/aicsingapore
**Agency for Integrated Care Website**	A one-stop portal on eldercare services and resources www.aic.sg
**DementiaHub.SG**	Singapore's one-stop resource portal on dementia www.dementiahub.sg

You can find these resources at www.aic.sg

Show you care with the CARA app

CARA, an initiative by Dementia Singapore, stands for '**Community, Assurance, Rewards and Acceptance**'. It provides access to an ecosystem of solutions. Download the app to:

- Access trusted resources and support
- Obtain a unique identifier and membership card for personsliving with dementia
- Receive support for the safe return of a wandering person living with dementia

Download on the App Store | GET IT ON Google Play

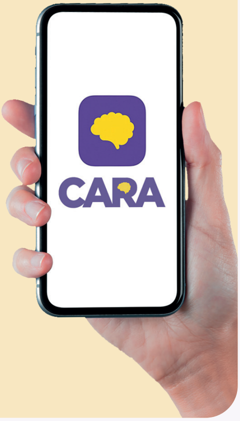

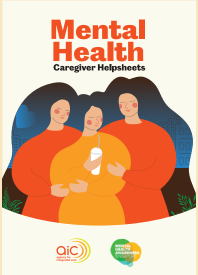

Mental Health Helpsheets for Caregivers

A set of mental health helpsheets focusing on 7 topics to support caregivers in their journey e.g. understanding loved one's mental health treatments, medications, preventing a relapse for the loved one. Available in English and Chinese.

Guide to Caring for Persons with Dementia (Parts 1 – 4)

A comprehensive guide to caregiving for persons with dementia, arranged in 4 parts to help caregivers along in their journey. Available in 4 languages.

Part 1: Knowing Dementia
Part 2: Caring for Yourself
Part 3: Planning Care
Part 4: Providing Care

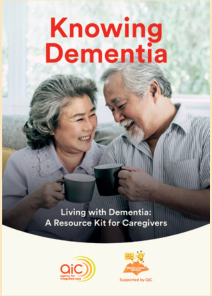

I. Community Partners Near You

If you need to seek help for persons with mental health conditions such as depression and would like to link them up with support and services, you may contact our community partners found in the insert. The support and services provided by the community partners include mental health assessment, counselling, caregiver training and advice on caring for people with mental health conditions.

Some caregivers experience high level of stress from caring for their loved ones. You may refer them to the 'Support & Services' found in the attached insert. These support and services help reduce caregiver burnout and stress.

Most people may prefer to see General Practitioners (GPs) instead of a specialist in the hospital. If you suspect someone may have a mental health condition, you may bring or refer them to their nearest mental health GP partners or polyclinics as listed for mental health assessment.

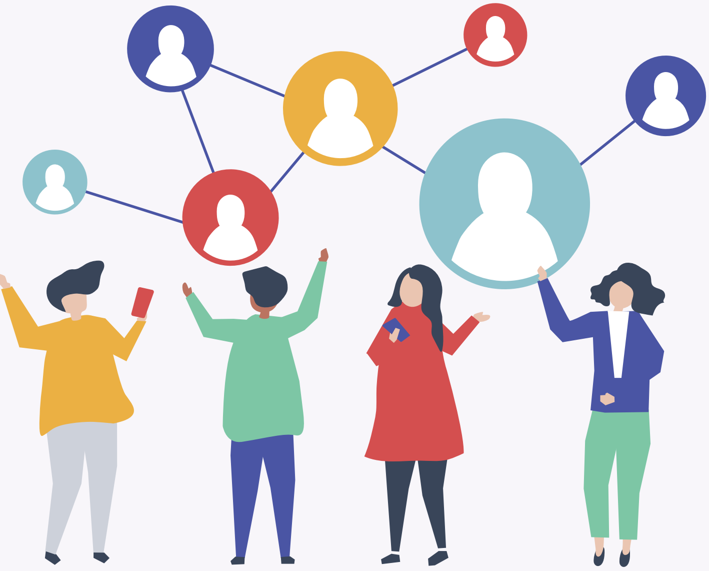

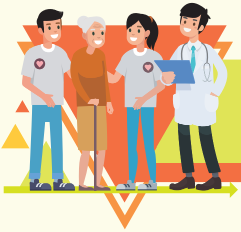

J. Hearing from Community Partners

Community intervention teams work hand in hand with community partners like General Practitioners (GPs) in the Mental Health GP Partnership Programme to provide support to persons with mental health needs. GP Dr Mark Yap and social worker Lee Soo Chin tell us more about their roles and how they work together to support their clients.

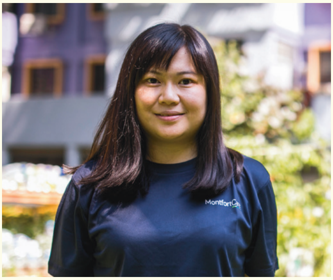

Lee Soo Chin, 32
Senior Social Worker,
COMIT Montfort Care

Q *What is the role of a community intervention team?*

Soo Chin (SC) : We provide counselling, psychotherapy, coordinate care and support caregivers of those with mental health conditions. In this way, we complement the care provided by GPs in managing clients with mild to moderate mental health needs so that they can continue to live well at home and in the community.

Q *GPs are usually the first point of contact for many of your clients. How do you work together to manage your client's condition?*

SC : While GPs are the first to identify the condition, our team provides social and emotional support for the clients and their caregivers to better manage their condition. For instance, if the client is confused about how to take their medication, we can explain it to them during our home visits. During such visits, we will also evaluate their condition and with their caregivers, develop or review their care plan.

Mark (M) : I have been working with the community intervention teams since 2014. I see about four to eight clients a month who may have anxiety condition, depression and schizophrenia. If they need more support such as counselling, I will email to the Agency for Integrated Care (AIC) for referral to the community intervention team for their follow up.

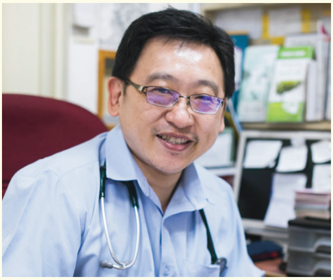

Dr Mark Yap, 48
Family Physician,
Cashew Medical & Surgery
Clinic Pte Ltd

How do you provide support for your clients?

M : People usually feel more comfortable talking to their family doctor. As a Mental Health GP Partner, I can also assess and diagnose patients with mental health conditions. When my patients tell me they feel unwell, I will also check if their symptoms may be due to an underlying mental health condition. If they are, I will refer them to the community intervention team for further support while I continue seeing them for the condition and provide medication if needed.

SC : When AIC sends us information on the clients for follow up, we will go down to their homes to evaluate their condition and develop or review their care plan with their caregivers. We also educate the clients about their conditions and work with other community partners like grassroots leaders and hospitals to provide support.

M : As for me, I often keep in touch with the community intervention team to stay up to date with my patient's condition. If I notice that someone has not returned to my clinic for follow ups or medication, I will also contact the team to see if my patient is doing okay.

The Heart of Care

Click
www.aic.sg

Call
1800 650 6060

Visit
AIC Link

The Agency for Integrated Care (AIC) aims to create a vibrant care community for people to live well and age gracefully. AIC coordinates and supports efforts in integrating care to achieve the best care outcomes for our clients.

We reach out to caregivers and seniors with information on staying active and ageing well, and connect people to services they need.

We support stakeholders in their efforts to raise the quality of care, and also work with health and social care partners to provide services for the ageing population. Our work in the community brings care services and information closer to those in need.

We hope you can take a few minutes to share your feedback with us by scanning the QR code. Thank you!

Information is as accurate as of August 2023
